# Implementing Rehabilitation Robotics in Routine Care: A Mapping Review of Service Delivery Models, Implementation Determinants, Strategies, and Real-World Outcomes

**DOI:** 10.3390/healthcare14162546

**Published:** 2026-08-14

**Authors:** Rocco Salvatore Calabrò, Andrea Calderone, Maria Felicita Crupi, Marcello Nucifora, Maurizio Lanza, Angelo Quartarone

**Affiliations:** Istituto di Ricovero e Cura a Carattere Scientifico (IRCCS) Centro Neurolesi Bonino-Pulejo, S.S. 113 Via Palermo, C.da Casazza, 98124 Messina, Italy; roccos.calabro@irccsme.it (R.S.C.); maria.crupi@irccsme.it (M.F.C.); marcello.nucifora@irccsme.it (M.N.); maurizio.lanza@irccsme.it (M.L.); angelo.quartarone@irccsme.it (A.Q.)

**Keywords:** rehabilitation robotics, routine care, implementation science, health services research, neurorehabilitation, service delivery, workflow, sustainability, implementation outcomes, real-world evidence

## Abstract

**Highlights:**

**What are the main findings?**

- Implementation evidence on rehabilitation robotics is concentrated around staffing, workflow, training, safety governance, feasibility and acceptability.
- Sustainment, penetration, documentation infrastructure, technical support, reimbursement and implementation cost remain underreported despite their importance for routine care.

**What are the implications of the main findings?**

- Robotic rehabilitation services should be evaluated as health service configurations, not only as devices or adjunctive clinical interventions.
- Future studies should report workforce, workflow, safety, documentation, cost and sustainment metrics to support reproducible and scalable routine-care implementation.

**Abstract:**

**Background/Objectives:** Rehabilitation robotics is increasingly used in neurorehabilitation, yet evidence on how robotic services are organized, maintained and scaled in routine care remains fragmented. This mapping review examined service-delivery models, implementation determinants, strategies, outcomes, workflows and resource signals for robotic rehabilitation in service-linked clinical care. **Methods:** Six search sources were queried from inception to 21 February 2026. Eligible reports were studies addressing rehabilitation robotics within clinical pathways and reporting an implementation-relevant element. Data from 55 studies were systematically charted and mapped to CFIR determinants, ERIC strategies and Proctor implementation outcomes. Design-appropriate appraisal was applied once per included report to contextualize methodological confidence. **Results:** Staffing and supervision were reported in 50 studies (90.9%), workflow and scheduling in 43 (78.2%), safety governance in 32 (58.2%) and training and competency in 29 (52.7%). Feasibility was mapped in 40 studies (72.7%), acceptability in 31 (56.4%) and adoption in 23 (41.8%), whereas sustainability, penetration, technical support, documentation infrastructure and implementation cost were less consistently reported. Two randomized trials were appraised with RoB 2 and four non-randomized comparative studies with ROBINS-I; the remaining 49 reports received design-appropriate methodological or reporting appraisal. **Conclusions:** Routine-care rehabilitation robotics is best understood as a service configuration rather than a device alone. Transferable implementation requires explicit reporting of workforce, workflow, safety, documentation, technical support, infrastructure, cost, equity and sustainment. Frequencies indicate reporting presence, not practical importance or causal influence. The proposed workflow, logic model and minimum reporting set are evidence-informed, inductively derived proposals requiring prospective validation.

## 1. Introduction

Rehabilitation robotics (RR) is increasingly present in neurorehabilitation services, where robotic gait trainers, overground exoskeletons, upper-limb end-effectors, gravity-compensation systems and multi-device robotic areas are used to increase practice dose, structure task-oriented therapy and generate objective information on training performance. Evidence from trials and systematic reviews supports selected motor benefits in stroke, spinal cord injury and other neurological conditions when robotic training is matched to the therapeutic aim, recovery phase and patient profile [1,2,3,4,5,6,7,8,9,10]. For healthcare delivery, however, clinical efficacy is only one part of the translation problem. A robotic intervention that is technically effective can still fail to become routine care if it cannot be scheduled, staffed, monitored, documented, maintained, reimbursed or sustained within ordinary rehabilitation pathways.

This health service perspective is especially important because RR is not a single intervention. Device families differ in footprint, acquisition cost, setup time, donning and doffing burden, supervision intensity, safety requirements, eligibility criteria, therapist competency requirements and capacity for semi-supervised or group delivery. Treadmill-based systems, end-effector gait robots, wearable exoskeletons, upper-limb devices, home-linked systems and robot-gym models therefore create different implementation problems. Their routine use is shaped by workflow fit, professional confidence, patient selection, documentation systems, technical support, maintenance pathways, space, procurement, reimbursement and local leadership [11,12,13,14,15,16,17,18]. These determinants may be as decisive for adoption and sustainment as the therapeutic mechanism of the device itself.

Implementation science provides a structured vocabulary for examining this gap. Determinants describe barriers and facilitators; implementation strategies describe actions used to introduce, adapt or sustain a service; and implementation outcomes indicate whether a pathway is acceptable, adopted, appropriate, feasible, delivered with fidelity, able to penetrate a setting, sustained and affordable [12,13,14,15,16,17,18]. In RR, these constructs are often embedded in clinical trials, routine-data studies, service evaluations, therapist surveys, qualitative studies and cost analyses rather than explicitly labelled as implementation research. As a result, evidence that is highly relevant to healthcare managers, clinicians and service planners remains fragmented across disciplines and difficult to compare across robot types, settings and health systems.

Economic and policy considerations add another layer of implementation risk. Robotic services require capital investment, training time, maintenance contracts, software or vendor support, space allocation, staff time and opportunity costs within fixed therapy schedules. Their value depends on utilization, slot fill, setup time, supervision ratio, throughput, substitution or supplementation of usual therapy, and reimbursement rules. Budget impact, procurement feasibility and regional access can therefore determine whether a robotic pathway is scalable, even when patients and clinicians perceive the technology as useful [19,20,21,22,23,24,25,26,27]. Because this evidence base spans qualitative, mixed-methods, quasi-experimental and observational designs, design-appropriate critical-appraisal criteria were prespecified rather than applying one undifferentiated quality scale [28,29,30].

A mapping/scoping approach is appropriate when a field is heterogeneous, terminology is inconsistent and the aim is to organize evidence rather than pool treatment effects [31,32,33,34,35]. Successful RR development also requires interdisciplinary work across clinicians, therapists, engineers, implementation scientists, health informaticians, safety specialists, decision makers and patients: therapeutic intent, device design, workflow, data governance, regulation and lived experience must be aligned before scale-up. This review therefore contributes an implementation-focused evidence map that differs from device-effectiveness reviews. It identifies scientific and clinical uncertainty alongside organizational, workforce, economic, digital-infrastructure and regulatory gaps, and organizes reported service models, determinants, strategies, outcomes and resource signals while distinguishing author-reported constructs from review-team-inferred constructs.

## 2. Materials and Methods

### 2.1. Design and Reporting Framework

This mapping review used Joanna Briggs Institute (JBI) guidance relevant to evidence mapping and scoping and an evidence-map logic to organize heterogeneous implementation-relevant evidence. Reporting followed the Preferred Reporting Items for Systematic Reviews and Meta-Analyses extension for Scoping Reviews (PRISMA-ScR), and selection was displayed in a PRISMA-ScR flow diagram [31,32,35]. The OSF project, initial protocol and three prespecified cross-database core search strings were registered prospectively on 14 February 2026 (OSF; 10.17605/OSF.IO/96G83); protocol version 1.1 was uploaded on 19 February 2026, before search execution. No meta-analysis was planned because the unit of interest was implementation content rather than clinical effect size. Critical appraisal was retained as an optional contextual component of the map. The completed PRISMA-ScR checklist is provided in Supplementary Table S1, and the methods-transparency register is provided in Supplementary Table S2A.

### 2.2. Eligibility Criteria, PCC Framework and Definition of Routine-Care Rehabilitation Robotics

Eligibility criteria were structured according to the Population, Concept and Context (PCC) framework. For Population, eligible studies included people receiving rehabilitation of any age and implementation stakeholders involved in the adoption, organization or delivery of robotic rehabilitation, including physiotherapists, occupational therapists, physicians, nurses, assistants, managers, biomedical engineers, technical staff, patients and caregivers. For Concept, the review focused on the integration of powered rehabilitation robots into clinical or health service care pathways, operationalized through service delivery models, workflows, implementation determinants, implementation strategies, implementation outcomes and resource-use signals. For Context, eligible settings included routine or service-linked rehabilitation care across inpatient, outpatient, community, home, mixed and system-level settings.

For the purposes of this review, routine-care RR was defined as the delivery, planning, rollout, evaluation, sustainment or service integration of powered robotic rehabilitation within clinical or health service pathways, outside purely laboratory-only proof-of-concept contexts. Routine care was not treated as a single setting, but as a service configuration. Accordingly, settings and delivery models were stratified as inpatient rehabilitation, outpatient or day-hospital services, clinic-to-home/community pathways, dedicated robotic areas, robot-gym/circuit models, multi-technology programmes and hub-and-spoke or networked models.

#### 2.2.1. Inclusion Criteria

Studies were eligible if they met all PCC elements and provided extractable implementation-relevant content. For Population, eligible studies included people receiving rehabilitation of any age and/or stakeholders involved in adopting, organizing, delivering, monitoring or evaluating robotic rehabilitation, including rehabilitation professionals, physicians, nurses, managers, technical staff, patients and caregivers. For Concept, studies had to address powered rehabilitation robots integrated into, planned for or explicitly linked to a rehabilitation care pathway. For Context, eligible settings included routine or service-linked inpatient, outpatient, day-hospital, community, home-linked, mixed or system-level rehabilitation services.

Eligible sources were full-text, database-indexed empirical reports, including qualitative, quantitative, mixed-methods, survey, pragmatic or clinical trial, service-evaluation, quality-improvement, routine-data, hybrid effectiveness–implementation, economic or resource-use studies. Eligible robotic technologies included robot-assisted gait trainers, end-effector systems, wearable or overground exoskeletons, upper-limb robotic devices, robotic therapy stations, robot-gym models and multi-technology pathways in which robotics was identifiable. Studies had to report at least one implementation-relevant element beyond generic clinical outcomes, such as service delivery, workflow, staffing, training, screening, safety governance, determinants, strategies, implementation outcomes, adoption, sustainment, resource use, technical support, maintenance, documentation, utilization, reimbursement or economic information. Clinical trials were eligible only when such information was extractable. When overlapping reports described the same programme, the most complete report was retained, and companion articles were used to clarify implementation details transparently.

#### 2.2.2. Exclusion Criteria

Reviews, systematic reviews, scoping reviews, narrative reviews, protocols without results, editorials, commentaries, opinion papers, letters without original data, abstract-only records and conference abstracts without full-text reports were excluded because they did not provide primary implementation data suitable for framework mapping. Studies were also excluded when the full text could not be retrieved after reasonable attempts.

Studies focused exclusively on laboratory proof-of-concept testing, engineering development, device design, control algorithms, technical validation, biomechanical performance, non-clinical feasibility or technology demonstrations without a link to rehabilitation service delivery were excluded. Assessment-only robotic systems, robotic measurement tools, diagnostic devices and non-therapeutic robotic applications were excluded unless embedded within a therapeutic rehabilitation pathway and accompanied by implementation-relevant service information. Non-rehabilitation robotics studies, including surgical robotics, industrial exoskeletons, telepresence robots, social robots and general assistive robots, were excluded. Digital interventions without a powered robotic rehabilitation component were excluded unless robotics was part of the intervention or service model. Efficacy-only reports were excluded when they reported only impairment, activity, gait, kinematic, neurophysiological or other clinical outcomes without information on workflow, staffing, safety, adoption, sustainment, cost, resource use, documentation or utilization. Borderline cases were assessed conservatively against the routine-care RR definition, and exclusion reasons were recorded during full-text screening to support transparent and reproducible study selection.

### 2.3. Information Sources, Search Strategy and Study Selection

The original searches were conducted in PubMed/MEDLINE, Embase, Web of Science Core Collection, Scopus, the Cochrane Library and CINAHL Complete through EBSCOhost from database inception to 21 February 2026. PubMed/MEDLINE was selected for biomedical and clinical coverage; Embase for complementary biomedical, device and European indexing; Web of Science and Scopus for multidisciplinary coverage spanning engineering, rehabilitation and health services; the Cochrane Library for CENTRAL and structured trial/evidence records; and CINAHL Complete for allied-health and rehabilitation literature. The searches were based on the three prespecified cross-database core strings deposited with the OSF protocol. The protocol preserved these strings without field tags; the native database-interface search histories were not retained. Search design and documentation were informed by PRESS [36]. Searches were limited to English and Italian full texts, with no publication-year restriction; language eligibility was applied during screening where an equivalent platform filter was unavailable. No otherwise eligible record was excluded solely on language grounds. Controlled vocabulary and free-text terms covered rehabilitation robotics, named devices, routine care, clinical practice, implementation, adoption, integration, workflow, service delivery, feasibility, acceptability, sustainability and cost. Supplementary Table S2B reports databases, interfaces, the historical search date, query-level yields and source totals; Supplementary Table S2C reproduces the OSF-deposited core strings verbatim alongside the preserved historical query-level yields; and Supplementary Table S2D documents strategy development, validation procedures and the limits of the retained search record. No retrospective rerun or updated search was performed.

After de-duplication using a structured duplicate-removal workflow [37], title/abstract and full-text screening were performed independently in Rayyan [38] by Andrea Calderone (A.C.) and Rocco Salvatore Calabrò (R.S.C.). Candidate records were evaluated against the predefined population, device, context and implementation-content criteria. Inter-reviewer agreement was substantial: Cohen’s kappa was 0.71 across 4546 title/abstract records and 0.75 across 107 full-text reports. Disagreements were resolved through discussion, with Angelo Quartarone (A.Q.) available for adjudication, and full-text exclusion reasons were recorded. Six included reports (6/55, 10.9%) and six of the 108 cited references (6/108, 5.6%) involved at least one member of the manuscript author team; these records were judged against the same prespecified criteria, and authorship did not alter eligibility decisions. The study-selection workflow, conflict-resolution procedures, agreement estimates and PRISMA-ScR reporting details are summarized in Supplementary Table S2E.

### 2.4. Data Charting and Framework Mapping

Data were charted in Microsoft Excel using a prespecified form piloted before full extraction. Extracted domains included bibliographic data, country, setting, population, robot category and model, clinical pathway, service-delivery workflow, staffing and supervision, training and competency, scheduling, safety governance, data capture, maintenance and technical support, infrastructure, implementation determinants, implementation strategies, implementation outcomes and economic/resource information. The study-level extraction map is reported in Supplementary Table S3, and study-level translational readiness signals are summarized in Supplementary Table S4.

Implementation determinants were mapped to Consolidated Framework for Implementation Research (CFIR) domains and constructs [39]. Strategies were mapped to Expert Recommendations for Implementing Change (ERIC) labels and, where possible, specified by actor, action, target, timing and operational detail [40,41]. Outcomes were mapped to Proctor’s taxonomy [42]. Coding retained author terminology when explicit. Otherwise, a construct was inferred only when a source passage contained an operationally matching barrier, facilitator, action or outcome; the verbatim source description remained linked to the mapped code. Distinct constructs could be assigned to one passage only when separate meanings were present, whereas ambiguous overlap was resolved with the narrower code or left unmapped. Explicit and inferred status was recorded for every assignment. One reviewer (R.S.C.) charted the data and a second (A.C.) verified all fields against the source text; coding differences identified during verification were discussed against the predefined rules. Because this was second-reviewer verification rather than independent duplicate charting, no formal charting-agreement coefficient was calculated. Supplementary Table S5 reports the rules, examples and explicit/inferred distributions. Data-charting safeguards and the operational rules used for borderline eligibility and framework-coding decisions are reported in Supplementary Table S2F,G.

### 2.5. Methodological Appraisal and Risk-of-Bias Assessment

Methodological appraisal was used as an optional component of this mapping review to contextualize design-specific credibility and reporting completeness. Four economic evaluations were assessed for reporting completeness with CHEERS 2022 and relevant ISPOR good-practice principles [20,21,22]. JBI design-specific checklists were applied to 11 quasi-experimental studies, eight case series, six analytical cross-sectional studies and two cohort studies [28]; CASP was applied to 11 qualitative studies [29], and MMAT to four mixed-methods studies [30]. RoB 2 was restricted to the two randomized trials [43], ROBINS-I to the four non-randomized comparative intervention studies [44], and QI-MQCS to three quality-improvement or service-model reports [45]. Each of the 55 included reports was classified by its actual design and assigned once to one primary instrument; the included report, rather than a parent trial or companion article, was the appraisal unit. CHEERS was not treated as a risk-of-bias instrument. Appraisal was not used for exclusion or quantitative weighting. The report-level design-to-instrument crosswalk, instrument criteria and study-level rationales are reported in Supplementary Tables S6 and S7.

The prespecified allocation rule distinguished the estimand and comparison structure. ROBINS-I was used for non-randomized reports that estimated an intervention or service-change effect through an explicit comparison between intervention groups or between defined pre- and post-implementation service periods. The JBI quasi-experimental checklist was used for non-randomized intervention reports without that comparative intervention-effect structure, including single-group pre–post or feasibility evaluations.

Methodological appraisal was completed by Rocco Salvatore Calabrò (R.S.C.) and verified by Andrea Calderone (A.C.). Disagreements were resolved by consensus, with Angelo Quartarone (A.Q.) acting as adjudicator when required. The six included reports involving members of the author team were handled as follows: each report was appraised exclusively by authors who were not involved in the corresponding source article.

The design-sensitive approach preserved the contribution of heterogeneous evidence without treating all reports as if they answered the same causal question. Service evaluations with limited comparative strength could still inform workflow, staffing, dose or technical barriers, while trials with stronger internal validity for clinical outcomes could remain sparse on adoption, sustainment or cost.

### 2.6. Synthesis and Subgroup Mapping

Synthesis was descriptive and matrix-based. Study characteristics were summarized as counts and percentages using 55 as the denominator unless otherwise stated. Instrument-specific appraisal results used the number assessed with that instrument as their denominator. Findings were stratified by publication period, country, setting, population, robot type, study design and implementation phase.

Subgroup mapping explored whether reporting patterns differed by robot type, diagnosis, setting, country/region and implementation phase. Because most studies were observational, qualitative, feasibility-oriented or service evaluations, subgroup analyses were descriptive and not inferential. A determinant–strategy–outcome matrix displayed co-reported patterns and plausible implementation pathways without implying causality. Subgroup mapping and the determinant–strategy–outcome matrix are provided in Supplementary Tables S8 and S9.

## 3. Results

### 3.1. Study Selection and Evidence Base

**Figure 1:**
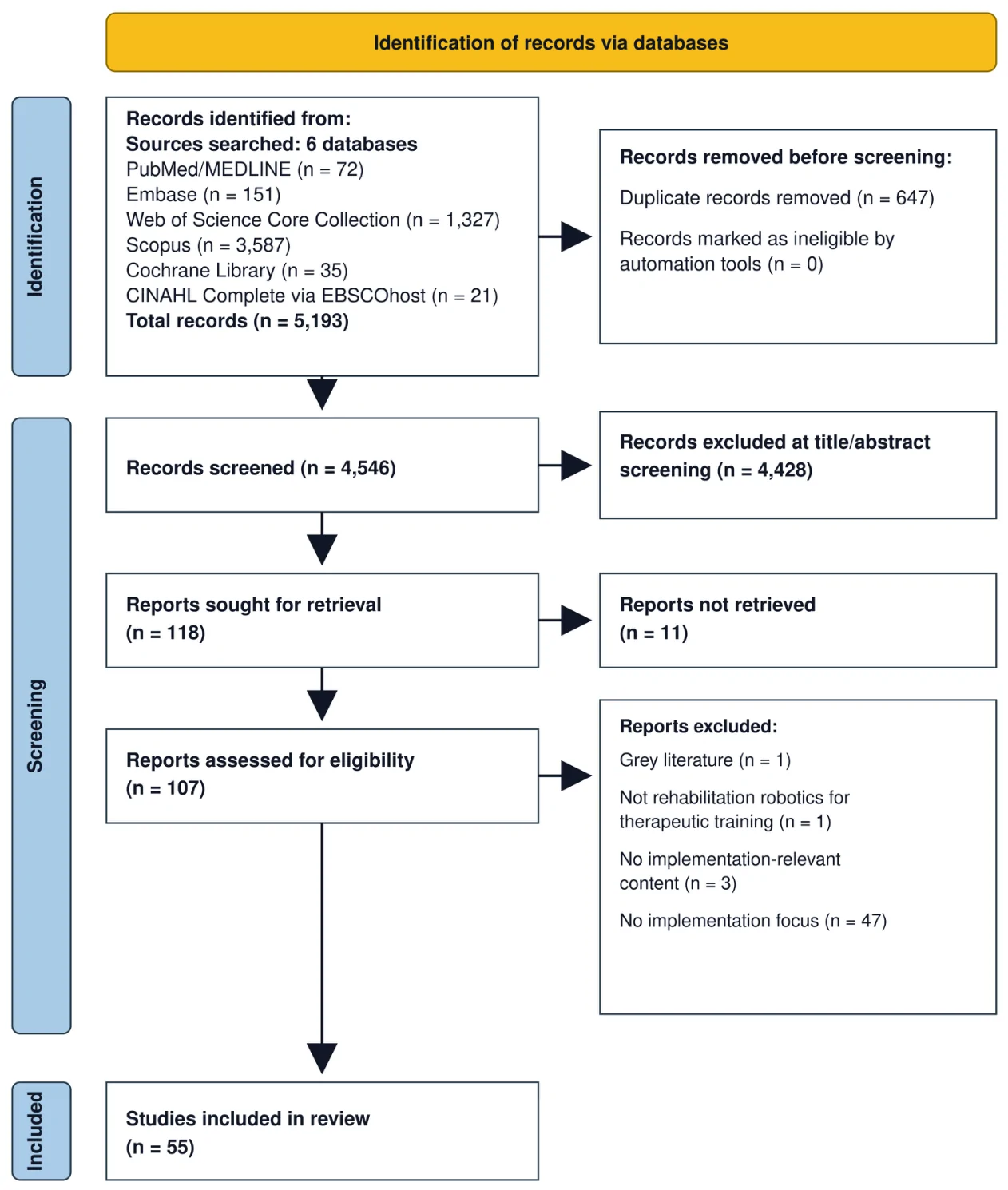
PRISMA-ScR flow diagram of study selection for the mapping review. The diagram reports database records, de-duplication, title/abstract screening, full-text assessment, full-text exclusion reasons and final inclusion of 55 studies.

The search identified 5193 records across six databases: PubMed/MEDLINE (*n* = 72), Embase (*n* = 151), Web of Science Core Collection (*n* = 1327), Scopus (*n* = 3587), the Cochrane Library (*n* = 35) and CINAHL Complete via EBSCOhost (*n* = 21). After removal of 647 duplicates, 4546 records were screened. Title/abstract screening excluded 4428 records, leaving 118 reports sought for retrieval. Eleven reports were not retrieved, and 107 full texts were assessed. Fifty-two reports were excluded at full text, leaving 55 included studies (Figure 1). Full-text exclusions were due to grey literature (*n* = 1), non-therapeutic RR (*n* = 1), no implementation-relevant content (*n* = 3) and no implementation focus (*n* = 47). No otherwise eligible record was excluded solely because of language.

The flow diagram highlights the effect of the implementation-relevance threshold. A large number of otherwise relevant RR studies were excluded because they focused only on impairment, activity, gait, kinematic or neurophysiological outcomes and did not describe service delivery. This was not a judgement that those studies lacked clinical value; rather, it reflected the review question. Clinical efficacy evidence and implementation evidence are complementary but answer different translational questions.

### 3.2. Study Characteristics, Populations, Robot Types and Implementation Phase

The included studies were published between 2010 and 2026 [46,47,48,49,50,51,52,53,54,55,56,57,58,59,60,61,62,63,64,65,66,67,68,69,70,71,72,73,74,75,76,77,78,79,80,81,82,83,84,85,86,87,88,89,90,91,92,93,94,95,96,97,98,99,100]. Most were published from 2020 onward (38/55, 69.1%). Italy was the most represented country (17/55, 30.9%), followed by the United States (7/55, 12.7%), multi-country studies (6/55, 10.9%), Australia (4/55, 7.3%), Canada (4/55, 7.3%), Germany (3/55, 5.5%) and Norway (3/55, 5.5%). Six reports (10.9%) included at least one member of the manuscript author team. These distributions describe the assembled evidence base and are relevant to transferability because reimbursement, workforce, procurement and research-network conditions differ across health systems (Table 1).

Stroke was the dominant clinical population (30/55, 54.5%), followed by spinal cord injury (7/55, 12.7%), other neurological or mixed populations (9/55, 16.4%), pediatric neurorehabilitation (2/55, 3.6%), Parkinson’s disease (1/55, 1.8%) and multiple sclerosis (1/55, 1.8%). Five studies (9.1%) were not tied to a single specified clinical population and instead addressed professional perceptions, service configuration or system-level implementation. Robot types varied: upper-limb robotics accounted for 18 studies (32.7%), gait robotics for 13 (23.6%), overground exoskeletons for 11 (20.0%), multi-technology programmes for eight (14.5%) and other or mixed technology configurations for five (9.1%).

Implementation phase was unevenly distributed. Planning or pre-implementation evidence accounted for nine studies (16.4%), early rollout/introduction for 13 (23.6%), routine delivery for 20 (36.4%), sustainment/optimization for six (10.9%) and unclear or unspecified phase for seven (12.7%). Long-term sustainment, penetration across sites and post-adoption competency maintenance were therefore documented less often than early feasibility or acceptability. The primary-design distribution comprised two randomized trials, four non-randomized comparative intervention studies, 11 quasi-experimental studies, eight case series, six analytical cross-sectional studies, two cohort studies, 11 qualitative studies, four mixed-methods studies, four economic evaluations or modelling studies and three quality-improvement or service-evaluation reports.

### 3.3. Service Delivery Components and Operational Workflow

Seven recurrent service-delivery components were identified (Table 2). These frequencies describe reporting presence and must not be interpreted as rankings of practical importance or causal influence. Staffing and supervision were reported in 50 studies (90.9%), workflow and scheduling in 43 (78.2%), safety governance in 32 (58.2%), training and competency in 29 (52.7%), infrastructure and space in 12 (21.8%), data capture and documentation in 14 (25.5%), and technical support/maintenance in seven (12.7%). Human delivery and session organization were reported more consistently than maintenance, documentation and audit infrastructure.

Data capture emerged as a cross-cutting weakness. Several devices automatically recorded repetitions, steps, walking time, assistance level or active training time, yet fewer studies described how these data were integrated into clinical documentation, audit or decision-making. Electronic health record fields, device logs and standardized session templates were rarely specified. This gap matters because sustainment depends on knowing whether a robotic service is being used, for whom, at what dose, with what adverse events and with what resource burden.

Routine delivery depended on linked operational decisions: screening shaped access, setup affected usable treatment time and documentation determined whether use could be audited. The extracted components were therefore treated as service building blocks that distinguish device availability from a pathway embedded in scheduling, governance and performance monitoring.

**Figure 2:**
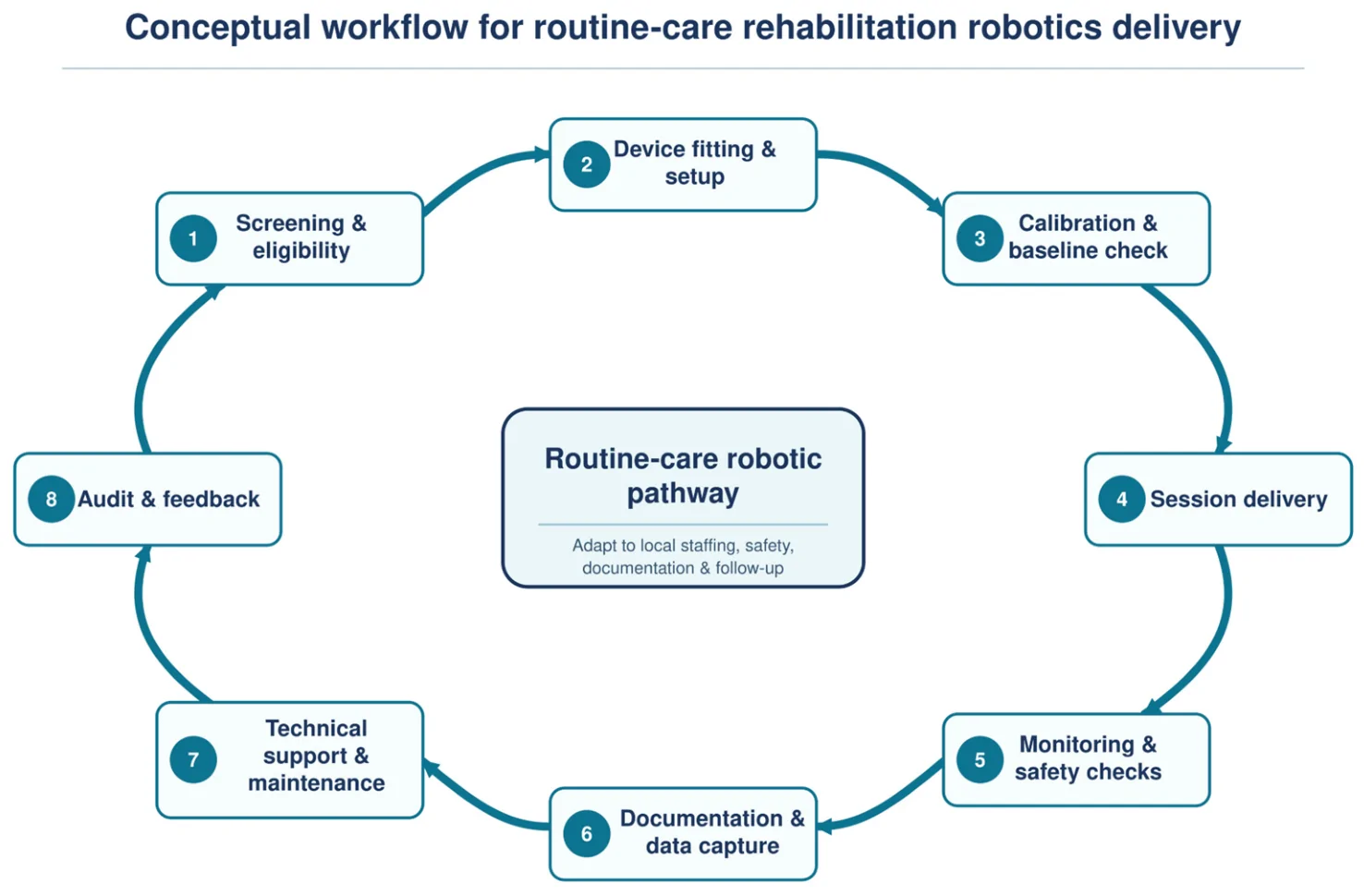
Conceptual workflow for routine-care rehabilitation robotics delivery. Eight recurrent operational phases were derived inductively from the included literature. This evidence-informed proposal is not a validated or consensus workflow.

Figure 2 presents an evidence-informed conceptual workflow derived inductively from recurrent operational phases: screening and eligibility, fitting and setup, calibration, session delivery, safety monitoring, documentation, technical support/maintenance and audit/feedback. The proposal requires local adaptation and prospective validation.

### 3.4. Implementation Determinants and Explicit Versus Inferred Coding

Implementation determinants were most frequently mapped to CFIR Inner Setting and Intervention Characteristics, with additional signals from Characteristics of Individuals, Process and Outer Setting. Common Inner Setting signals included available resources, access to knowledge and information, readiness for implementation, workflow compatibility and staffing capacity. Intervention Characteristics commonly included complexity, adaptability, design quality, cost and relative advantage. Process constructs included planning, engaging champions or super-users, executing local protocols and reflecting/evaluating through service or device metrics.

Intervention complexity was not limited to mechanical or software complexity. It also included fitting burden, parameter selection, safety monitoring, therapist–patient–robot interaction and the extent to which a device required changes in professional roles. Characteristics of individuals were reported unevenly but included clinician confidence, perceived usefulness, perceived ease of use, technology anxiety, professional identity, patient motivation, comfort, fatigue, fear, expectations, eligibility, tolerance and caregiver availability.

Outer Setting determinants were less frequently reported but highly consequential when present. Funding, reimbursement, procurement, patient access, regional service configuration and policy constraints shaped adoption and sustainability in economic, hub-and-spoke and exoskeleton studies. These determinants were not always frequent in counts, but their practical weight was substantial because they influenced whether services could purchase devices, maintain them, train staff and offer access beyond selected specialist centres. Explicit use of CFIR, ERIC and Proctor terminology in primary studies was uncommon, and most framework assignments were inferred by the review team from operational descriptions (Supplementary Table S5).

Primary studies often described limited space, staff confidence, setup burden, maintenance delays or patient tolerance without framework terminology. Mapping supported comparability but required interpretive judgments by the review team; explicit and inferred codes are therefore separated in Supplementary Table S5.

### 3.5. Implementation Strategies and Outcomes

**Figure 3:**
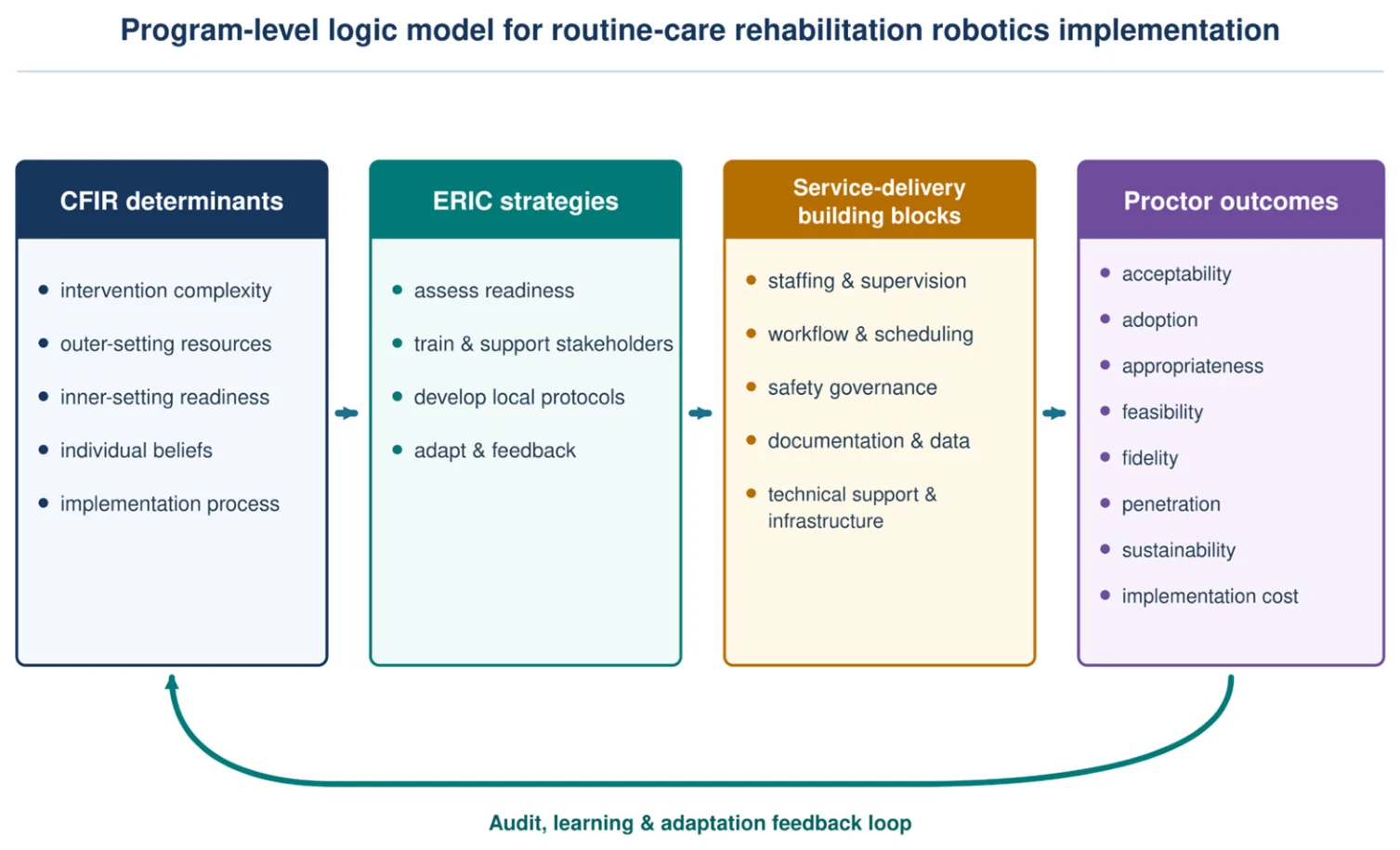
Programme-level logic model for routine-care rehabilitation robotics implementation. The model links CFIR determinants, ERIC strategies, service-delivery building blocks and Proctor outcomes with audit, learning and adaptation. It was derived inductively from the included literature and has not been formally validated.

ERIC-mapped strategies were predominantly pragmatic and service-facing, including training, educational materials, champions, local protocols, workflow redesign, contextual adaptation, audit and feedback, patient/caregiver engagement, technical assistance and service reorganization. Figure 3 presents an evidence-informed programme-level logic model linking determinants, strategies, service building blocks and implementation outcomes, with a learning feedback loop.

Implementation outcomes were dominated by feasibility and acceptability (Table 3). Feasibility was reported in 40 studies (72.7%), acceptability in 31 (56.4%), adoption in 23 (41.8%), appropriateness in 18 (32.7%), fidelity in eight (14.5%), penetration in eight (14.5%), sustainability in seven (12.7%) and implementation cost in nine (16.4%). Measures were heterogeneous and included System Usability Scale scores, bespoke satisfaction surveys, interviews, session logs, device utilization metrics, completion rates, adverse-event reports, cost models and administrative service indicators.

Implementation cost was less frequently reported than feasibility or acceptability, but it provided some of the most actionable information for service decision-making. Cost-related outcomes were strongest when studies specified staff minutes, supervision ratios, device amortization, maintenance assumptions, utilization, slot fill or budget impact. Studies reporting only device price or general affordability concerns were useful as signals but did not provide enough information for local planning.

Strategies were most transferable when paired with operational measurement: competency development with training hours or certified users, and workflow redesign with setup time, slot use or delivered dose. Reporting the actor, action, target, timing and intended outcome distinguishes reproducible implementation strategies from informal local workarounds.

### 3.6. Economic, Policy and Resource-Use Signals

Economic and resource-use evidence remained limited but was more decision-relevant than its frequency alone suggests. Cost analyses highlighted recurring drivers: device acquisition, maintenance, training, supervision ratio, therapist time, setup time, utilization, slot fill, substitution of conventional therapy and patient throughput. Several studies showed that the same robotic technology could have different economic implications depending on the service model. Multi-patient supervision, robot-gym circuits, co-located devices, portable bedside systems and higher utilization improved cost profiles in some models, whereas one-to-one delivery, low slot fill, extensive setup and limited reimbursement increased resource burden.

Policy and reimbursement factors were often reported as barriers rather than formally evaluated. Procurement pathways, funding availability, device maintenance and reimbursement rules were rarely integrated into prospective implementation designs. As a result, sustainability evidence remains thin, especially for services beyond single centres or pilot phases. Economic interpretation was also limited by local assumptions: cost per session or per pathway depends on depreciation period, maintenance contract, staff salary, supervision model, number of devices, expected utilization and whether robotic sessions replace or supplement usual therapy.

Cost could not be separated from delivery model. Acquisition expense might be offset by higher utilization, group supervision or reduced idle time, whereas intermittent one-to-one use increased resource burden. Decision makers therefore need staff time, maintenance, depreciation, training, throughput and substitution assumptions rather than device price alone.

### 3.7. Subgroup Patterns, Workflow Archetypes and Translational Readiness

Subgroup mapping suggested different implementation emphases across robot types. Upper-limb robotics and robot-gym models most often emphasized workflow efficiency, group supervision, usability, dose quantification and staffing ratios. Gait robotics and overground exoskeletons more often emphasized screening, safety governance, patient fit, setup burden, physiological tolerance and certified training. Multi-technology programmes and regional networks emphasized infrastructure, pathway coordination and sustainability (Supplementary Table S8).

Setting also mattered. Inpatient studies focused on fixed therapy schedules and medically complex patients; outpatient and community studies emphasized travel, adherence and continuity; system-level reports addressed access and referral pathways. The determinant–strategy–outcome matrix organized these co-reported patterns into propositions for future prospective testing (Supplementary Table S9).

Workflow archetypes were treated as translational readiness signals because they describe how services are configured. They were derived inductively from the included literature, are not mutually exclusive and have not been validated as a taxonomy or endorsed as consensus recommendations (Table 4).

**Figure 4:**
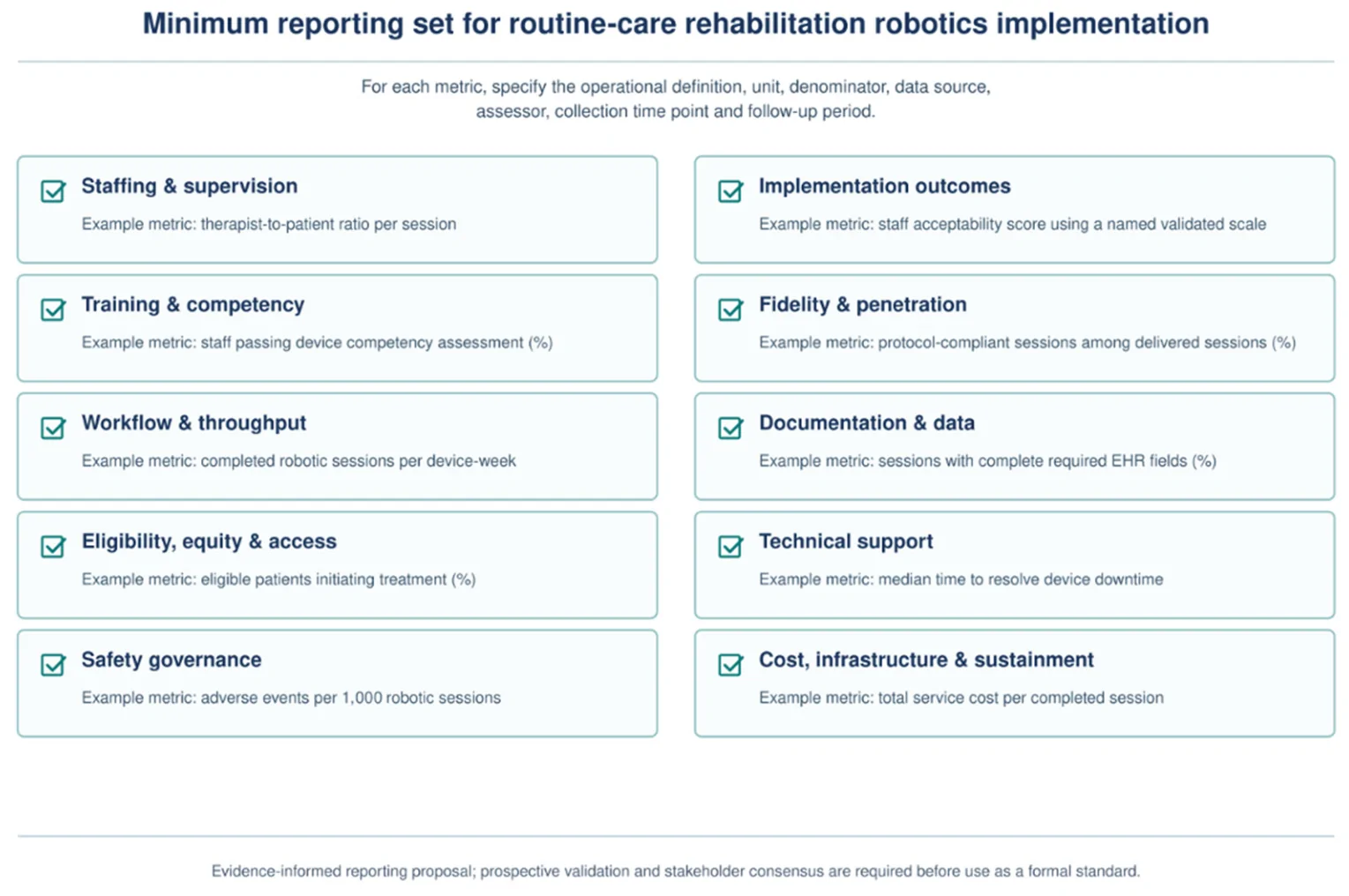
Minimum reporting set for routine-care rehabilitation robotics implementation. The checklist specifies prospective service and implementation metrics and the reporting attributes needed for reproducibility. It was derived inductively and requires validation and stakeholder consensus before use as a formal standard.

To improve transferability, Figure 4 and Supplementary Table S10 propose a minimum reporting set. Future studies should measure acceptability, adoption, appropriateness, feasibility, fidelity, penetration, sustainability and implementation cost alongside workforce, training, throughput, documentation completeness, downtime, maintenance, adverse events, equity, infrastructure and energy/resource requirements. Each metric should specify its unit, denominator, data source, assessor, collection time point and follow-up period.

The archetypes and reporting set should be viewed as evidence-informed proposals for prospective validation. Their purpose is to make the operational architecture of a service visible and testable across settings, not to prescribe a single model of care.

### 3.8. Methodological Confidence and Risk-of-Bias

Methodological appraisal included each of the 55 reports once and used the included report, not an associated parent trial or companion article, as the unit of appraisal. Each appraisal row corresponds to the same report in Supplementary Table S3, and no report was assigned to more than one instrument. The design distribution by appraisal instrument comprised two randomized trials, four non-randomized comparative intervention studies, 11 quasi-experimental studies, eight case series, six analytical cross-sectional studies, two cohort studies, 11 qualitative studies, four mixed-methods studies, four economic evaluations or modelling studies and three quality-improvement or service-evaluation reports. Instrument-specific results were not pooled because the tools assess different methodological properties (Supplementary Tables S6 and S7).

**Figure 5:**
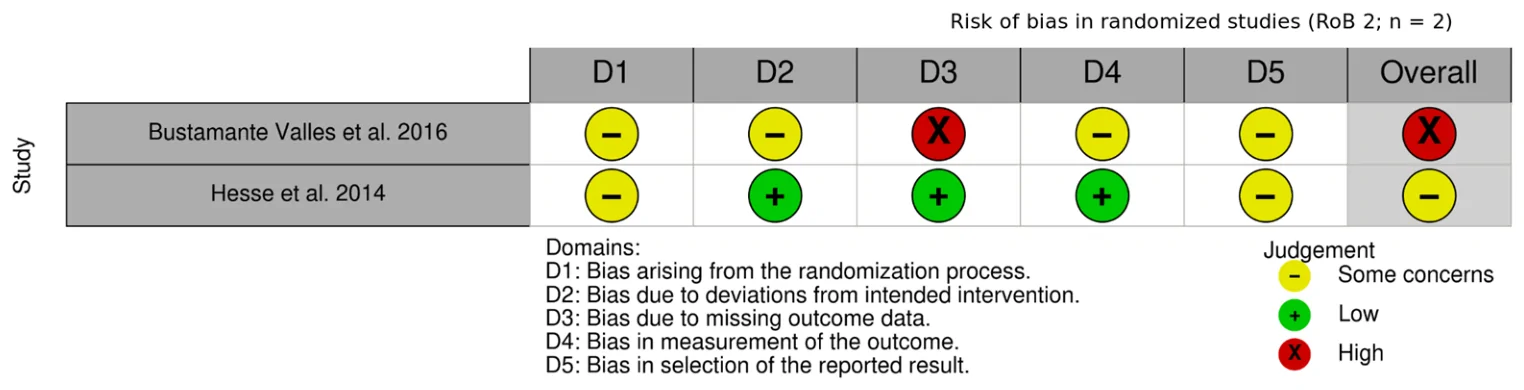
Risk-of-bias in randomized studies (RoB 2; *n* = 2). Domain-level and overall judgments are shown for the two randomized trials included in Supplementary Table S3. Appraisal contextualized the evidence map and was not used for exclusion or weighting.

Among the two randomized trials, Bustamante Valles et al.’s [63] was judged high-risk overall because post-randomization attrition, unclear concealment, baseline imbalance and unblinded outcome assessment reduced confidence. Hesse et al.’s [94] was judged to have some concerns: restricted randomization and blinded primary-outcome assessment were reported, but allocation concealment and a prespecified analysis plan were insufficiently detailed (Figure 5; *n* = 2).

**Figure 6:**
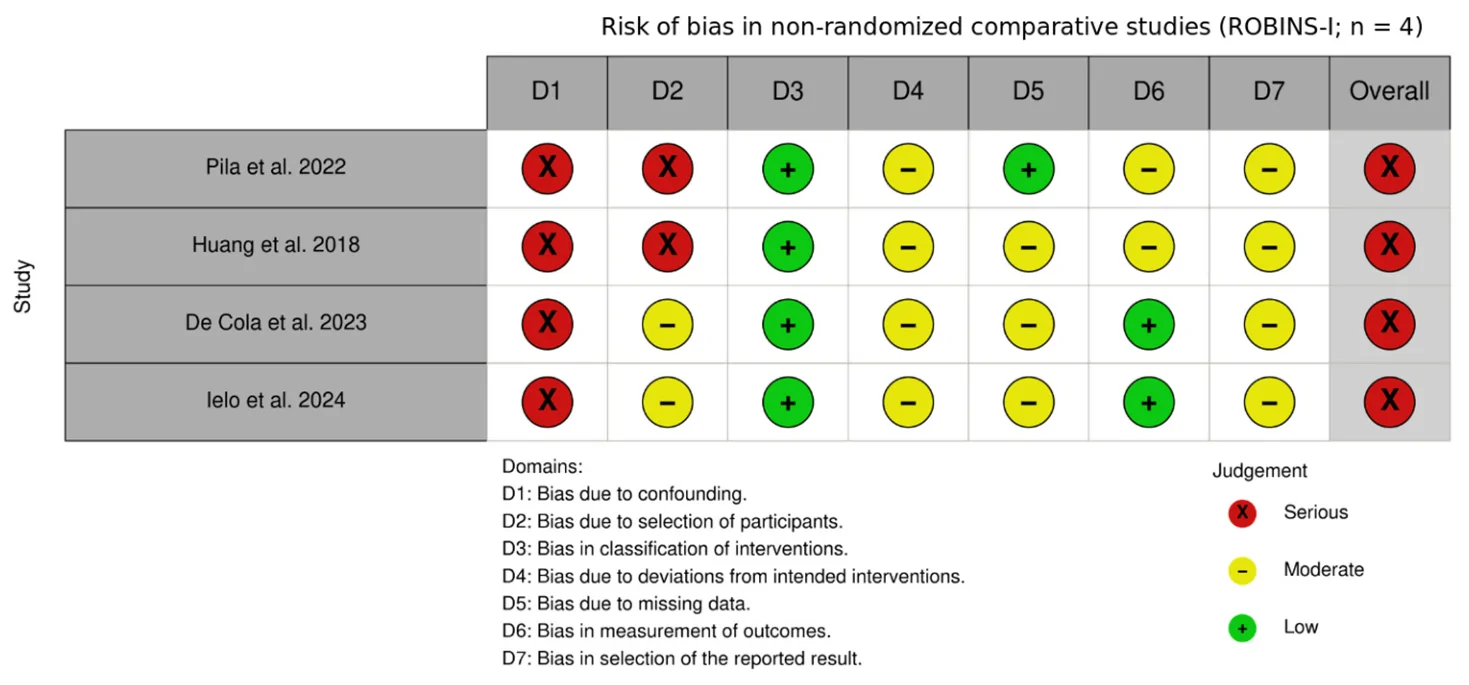
Risk-of-bias in non-randomized comparative studies (ROBINS-I; *n* = 4). Domain-level and overall judgments are shown only for the four eligible comparative studies.

All four non-randomized comparative studies [50,88,90,91] were judged at serious overall risk-of-bias, principally because group allocation or pre/post service periods were confounded by clinical selection, setting, calendar time or concurrent system change. Intervention classification and administrative outcome measurement were generally clearer than control of confounding (Figure 6; *n* = 4).

The remaining 49 reports received design-appropriate appraisal: JBI checklists for 11 quasi-experimental studies, eight case series, six analytical cross-sectional studies and two cohort studies; CASP for 11 qualitative studies; MMAT for four mixed-methods studies; QI-MQCS for three quality-improvement or service-evaluation reports; and CHEERS 2022 plus relevant ISPOR principles for four economic evaluations. Common limitations were selected or small samples, uncontrolled designs, incomplete non-response or reflexivity information, and context-dependent costing assumptions. These tools and their labels were interpreted separately rather than converted to a common risk score. De Cola et al. [90] and Ielo et al. [91] were classified as comparative intervention studies because each compared administrative service outcomes across defined periods before and after activation of the regional network; this analytic design was distinguished from a descriptive service-model report.

The appraisal supports cautious, design-specific use of the map. Methodologically limited studies could still reveal workflow, staffing, acceptability or resource barriers, but no appraisal category established the superiority of an implementation strategy. Economic reporting was most decision-relevant when perspective, resource inputs, utilization, uncertainty and transferability were explicit.

## 4. Discussion

### 4.1. Principal Findings and Health Service Delivery Implications

This mapping review synthesizes implementation-relevant evidence on RR as a health service delivery problem rather than only a device or clinical-effectiveness problem. Across 55 included studies, the most frequently quantified signals concerned staffing, workflow, training, safety governance and acceptability. Screening and utilization were recurrent qualitative or operational themes but were not assigned standalone frequencies in Table 2 and Table 3. Conversely, long-term sustainability, penetration, technical support, documentation infrastructure and implementation cost were less consistently reported. The central implication for healthcare readers is that RR becomes scalable routine care only when robotic capabilities are translated into service configurations that can be staffed, scheduled, monitored, documented, paid for and sustained.

The findings suggest that routine care is not a single setting but a service configuration. A robot embedded within an ordinary inpatient therapy session differs substantially from an exoskeleton programme requiring certified staff, a robot-gym circuit, a clinic-to-home pathway or a regional hub-and-spoke network. Treating these configurations as equivalent obscures the operational requirements that determine adoption and sustainability. For service leaders, the practical question is therefore not only whether a robot is clinically promising, but which pathway can deliver the intended therapeutic dose safely and sustainably for defined patient groups.

Workflow and workforce emerged as central implementation levers. Setup time, donning and doffing, calibration, patient transfers, therapist–patient–robot interaction, scheduling conflicts and documentation demands can absorb a meaningful proportion of fixed therapy time. These constraints may affect adoption, delivered dose, fidelity and staff acceptance. Strategies such as competency-based training, local protocols, co-location of devices, protected slots, group supervision, circuit models and audit of utilization were frequently reported, but their effect on adoption or sustainment has rarely been tested prospectively. Future studies should measure intermediate mechanisms such as staff confidence, setup time, slot fill, documentation completeness and supervision intensity.

Safety governance is inseparable from workflow, especially for gait robotics and overground exoskeletons. Services need reproducible screening, contraindication criteria, physiological monitoring, adverse-event definitions, stop rules and escalation pathways. Several studies described such elements, but safety processes were inconsistently operationalized as implementation outcomes. This matters because safety requirements shape staffing ratios, eligibility, throughput and scalability. In routine care, the implementation question is not whether a device can be used safely in principle, but whether a service can deliver it safely at scale under ordinary staffing and documentation conditions.

Economic and policy findings were underreported relative to their importance. High acquisition costs, maintenance, reimbursement uncertainty and procurement constraints appeared repeatedly, particularly in exoskeleton and network-level studies, but few studies quantified how these barriers affected adoption, utilization or long-term viability. Economic value depends strongly on service configuration. The same technology may be financially burdensome in a one-to-one model but more sustainable when integrated into multi-patient supervision, high-utilization slots or mixed protocols that substitute for part of conventional therapy. Decision makers need transparent information on capital cost, depreciation, maintenance, training, staff time, patient throughput, downtime, opportunity cost and reimbursement assumptions.

The review also highlights an equity and access dimension. Robotic rehabilitation may increase access when it supports community or home pathways, regional networks or efficient group delivery. It may widen inequities if access remains limited to specialist centres, privately funded services or patients who can travel repeatedly. Implementation studies should therefore report reach, eligibility exclusions, reasons for non-use, geographic distribution, patient burden and penetration across sites. These data are essential for judging whether a robotic pathway contributes to scalable care or remains a locally available innovation.

### 4.2. Framework-Based Interpretation and Future Implementation Research

CFIR, ERIC and Proctor’s implementation-outcomes taxonomy provided a coherent structure for organizing an otherwise heterogeneous evidence base spanning clinical, organizational, technical and economic dimensions. Nevertheless, explicit use of implementation-science terminology was uncommon in the primary studies. Many determinants, strategies and outcomes therefore had to be mapped retrospectively from operational descriptions of staffing, workflow, training, safety, technical support, resource constraints and service adoption. The use of source-linked decision rules, second-reviewer verification and separate classification of explicit and inferred constructs strengthened transparency and consistency, but could not eliminate interpretive subjectivity. Future implementation studies should therefore embed theory prospectively by specifying relevant determinants, implementation strategies, hypothesized mechanisms and measurable outcomes before data collection. Authors should also report how constructs are operationalized, who assesses them, the data sources and denominators used, and the time points at which adoption, fidelity, penetration and sustainment are evaluated.

No single framework is likely to capture the full complexity of rehabilitation robotics implementation. CFIR can characterize multilevel contextual determinants, ERIC can support the selection and specification of implementation strategies, and Proctor’s taxonomy can define implementation outcomes. These approaches may be complemented by RE-AIM to examine reach, effectiveness, adoption, implementation and maintenance; EPIS to distinguish exploration, preparation, implementation and sustainment phases; and Normalization Process Theory to investigate how robotic pathways become embedded in everyday professional practice [16,101,102]. Their combined and prospectively specified use could move the field beyond descriptive reporting toward testable determinant–strategy–mechanism–outcome pathways, while supporting comparison across devices, clinical populations, service configurations and healthcare systems.

Hybrid effectiveness–implementation designs, pragmatic trials, stepped-wedge evaluations, cluster designs, embedded service audits and prospective budget-impact analyses would strengthen the field [103,104]. Such designs could test whether screening protocols improve appropriate adoption, whether standardized documentation improves fidelity, whether multi-patient supervision improves throughput without reducing safety, and whether maintenance and technical support reduce downtime. These designs would convert the co-reported pathways identified in this review into testable implementation hypotheses.

Clinical uncertainty can itself impede adoption. Even where capital, technical support and specialist services are available, clinicians may delay integration when expected benefits, eligible populations or added value over well-delivered conventional therapy remain uncertain. This barrier is distinct from affordability: it concerns confidence in the clinical rationale and the cognitive work of patient selection. A Japanese umbrella review found condition- and outcome-specific benefits but also emphasized variable evidence quality and the need for stronger syntheses [105]. It should be read as effectiveness context, not as an implementation study or an additional included record. The comparison underscores why high-resource systems, including Japan, may possess technology without uniform routine adoption.

Transferability also depends on system architecture. Workforce availability, reimbursement, procurement cycles, clinical engineering support, device regulation, digital infrastructure and continuity between hospitals and community services may alter the feasibility of the same model. Highly resourced technology centres may absorb training, maintenance and downtime that smaller territorial or low- and middle-income services cannot. Future comparative implementation studies should therefore report these contextual variables and test adaptation rather than assuming universal portability.

Demographic change strengthens the need for such prospective evaluation. Global rehabilitation need is expected to rise with population ageing and chronic disability, while health systems face persistent workforce constraints [106]. Artificial intelligence may support multimodal monitoring, adaptive progression and programme planning, but current rehabilitation evidence remains heterogeneous and requires clinical validation, interoperable data and accountable human oversight [107]. Generative and agentic systems should therefore be evaluated as bounded decision-support and workflow components, with predefined authority, verification, privacy, bias and failure-management rules. Patient digital twins are similarly an emerging modelling concept, not an established RR implementation tool; definitions and clinical maturity remain variable [108]. Prospective studies could test whether these approaches improve selection, personalization, documentation or follow-up without increasing inequity or verification burden. Connections with smart-city or smart-territory infrastructure may eventually support accessible transport, home monitoring and regional data exchange, but require interoperability, consent and governance across organizations. Scale-up should also quantify electricity use, computing demand, device lifecycle, space, network resilience and maintenance rather than treating energy and infrastructure as unlimited. Economic, organizational and environmental resource evaluations should be specified before deployment and repeated during sustainment.

For clinical leaders, the findings imply that implementation planning should begin before device purchase. Procurement decisions should be linked to a clear service question: which patients will be offered robotics, who will screen them, where sessions will occur, how staff will be trained, how adverse events will be recorded, how utilization will be audited and how maintenance will be managed. These questions are often treated as local logistics, but they are central determinants of adoption and sustainment. A device introduced without a service model may produce isolated enthusiasm, whereas a device introduced with defined roles, scheduling rules and audit metrics is more likely to become part of routine practice.

Implementation success should be judged through standardized prospective measures rather than feasibility and acceptability alone. Core outcomes should include acceptability, adoption, appropriateness, feasibility, fidelity, penetration, sustainability and implementation cost, supplemented by staff minutes, supervision ratio, training completion, setup time, throughput, downtime and repair response, documentation completeness, adverse events and stop-rule activations, equity of access, infrastructure and energy/resource use. Each measure should state its operational definition, unit, denominator, source, assessor, timing and follow-up.

Finally, future studies should move from descriptive reporting toward prospective learning systems. Routine device logs, electronic health record fields, adverse event forms, staffing records and patient selection data could be combined into implementation dashboards. Such dashboards would allow services to monitor dose, safety, utilization, missed sessions, reasons for non-use and resource burden. They would also make it possible to compare pathways across centres without assuming that all robotic services are equivalent. This approach is particularly relevant for multicenter rehabilitation networks, where local flexibility must be balanced with shared definitions, common metrics and transparent governance. In this sense, implementation research should be embedded into routine rehabilitation governance rather than added retrospectively after clinical adoption has already occurred.

Strengths include prospective protocol registration, broad multidisciplinary searching, duplicate screening, explicit eligibility criteria, source-linked framework mapping and design-appropriate report-level appraisal of all 55 reports. Limitations remain. The database-only strategy omitted grey literature, citation chasing, PsycINFO, conference proceedings and languages other than English or Italian. The OSF protocol deposited the three cross-database core strings before search execution, and CINAHL Complete was searched through EBSCOhost; preserved export fields and query-level yields are reported in Supplementary Table S2B,C. However, the native database-interface search histories and exact executed interface syntax were not retained. Supplementary Table S2C therefore reproduces the deposited core strings verbatim alongside the historical query-level yields and does not present reconstructed native syntax; no retrospective rerun or updated search was performed. Italy contributed 17/55 reports (30.9%); 6/55 included reports (10.9%) and 6/108 cited references (5.6%) involved at least one manuscript author. Although prespecified criteria and duplicate screening were applied, language restriction, geographic concentration and familiarity with particular research networks may have increased the visibility of Italian evidence. Most reports were single-centre and heterogeneous, coding based on review-team inference remained interpretive, and appraisal contextualized the map rather than certainty of effectiveness. Transfer and determinant–strategy–outcome hypotheses therefore require prospective validation.

## 5. Conclusions

This mapping review identified 55 studies reporting implementation-relevant evidence for RR in routine or service-linked care. The most frequently quantified signals concerned staffing, workflow, training, safety governance, acceptability and feasibility. Screening and utilization recurred as qualitative or operational themes and were not assigned standalone frequencies; procurement, reimbursement, maintenance, documentation, penetration, sustainability and implementation cost were less consistently quantified. The evidence does not establish the comparative effectiveness of implementation strategies.

For healthcare systems, the translational value of RR depends on implementation evidence with rigour comparable to clinical efficacy evidence. RR can move from local innovation to scalable routine care only when service models, workforce requirements, workflow, documentation, safety governance, cost assumptions and sustainment are reported and evaluated transparently. Future work should use standardized reporting, explicit framework-based design, prospective hybrid effectiveness–implementation studies, long-term sustainment measures and economic evaluation to determine which robotic pathways can be reproduced safely, equitably and sustainably across real-world rehabilitation services.

The field now requires pathway-centred evaluation with visible staffing, workflow, data, safety, maintenance, economic and sustainment assumptions. The proposed archetypes, logic model and minimum reporting set are pragmatic, evidence-informed starting points for validation, not universal recommendations. Consistent prospective measurement would allow clinicians, managers and policymakers to judge whether a robotic programme can be adapted and sustained across differently resourced services.

## Figures and Tables

**Table 1:** Characteristics of included studies (*N* = 55).

Domain	Category	*n* (%)
Publication year	2010–2014	3 (5.5%)
	2015–2019	14 (25.5%)
	2020–2026	38 (69.1%)
Country	Italy	17 (30.9%)
	USA	7 (12.7%)
	Multi-country	6 (10.9%)
	Australia	4 (7.3%)
	Canada	4 (7.3%)
	Germany	3 (5.5%)
	Norway	3 (5.5%)
	France	2 (3.6%)
	Switzerland	2 (3.6%)
	Austria	1 (1.8%)
	Mexico	1 (1.8%)
	Singapore	1 (1.8%)
	Republic of Korea	1 (1.8%)
	Hong Kong	1 (1.8%)
	Sweden	1 (1.8%)
	UK	1 (1.8%)
Setting	Inpatient	25 (45.5%)
	Mixed inpatient/outpatient	5 (9.1%)
	Rehabilitation service (unspecified)	12 (21.8%)
	Clinic-to-home/community	5 (9.1%)
	System-level/multi-setting	6 (10.9%)
	Other/unclear	2 (3.6%)
Population	Stroke	30 (54.5%)
	Spinal cord injury	7 (12.7%)
	Other neurological/mixed	9 (16.4%)
	Parkinson’s disease	1 (1.8%)
	Multiple sclerosis	1 (1.8%)
	Pediatric neuro	2 (3.6%)
	Not specified	5 (9.1%)
Robot type	Gait robotics	13 (23.6%)
	Overground exoskeleton	11 (20.0%)
	Upper-limb robotics	18 (32.7%)
	Multi-technology programme	8 (14.5%)
	Other/mixed technology	5 (9.1%)
Study design	Randomized trial	2 (3.6%)
	Non-randomized comparative intervention	4 (7.3%)
	Quasi-experimental	11 (20.0%)
	Case series	8 (14.5%)
	Analytical cross-sectional	6 (10.9%)
	Cohort	2 (3.6%)
	Qualitative	11 (20.0%)
	Mixed methods	4 (7.3%)
	Economic evaluation/modelling	4 (7.3%)
	Quality improvement/service evaluation	3 (5.5%)
Implementation phase	Planning/pre-implementation	9 (16.4%)
	Early rollout/introduction	13 (23.6%)
	Routine delivery	20 (36.4%)
	Sustainment/optimization	6 (10.9%)
	Not specified	7 (12.7%)

Note: *n* (%) values were calculated over the 55 included studies using the study-level extraction map; each study was counted once per domain.

**Table 2:** Routine-care robotics service-delivery components.

Operational Component	Reported in *n* (%)	What to Report	Example Operational Metrics
Staffing & supervision	50 (90.9%)	Staff roles, supervision level, skill-mix, and any group-delivery or assistant support.	Staff-to-patient ratio; supervision model (physiotherapist-led vs. shared); minutes of staff time/session.
Training & competency	29 (52.7%)	Initial onboarding, competency checks, refresher training, and champion/super-user structure.	Training hours per staff; competency checklist completion; refresher frequency.
Workflow & scheduling	43 (78.2%)	Session structure, setup/donning–doffing, scheduling rules, circuit models, and throughput constraints.	Setup time; sessions/week; slot fill rate; repetitions/session; circuit station time.
Safety governance	32 (58.2%)	Screening, contraindications, monitoring, adverse event definitions, and escalation/stop rules.	Adverse-event rate per 1000 sessions; screening failure rate; vital sign monitoring protocol.
Data capture & documentation	14 (25.5%)	How sessions are documented and monitored (logs, device data, electronic health record fields, audit trail).	Device utilization logs; adherence logs; data fields captured per session; completeness %.
Tech support & maintenance	7 (12.7%)	Maintenance plan, troubleshooting pathway, and vendor/biomedical engineering support.	Downtime hours/month; mean time to repair; planned maintenance frequency.
Infrastructure & space	12 (21.8%)	Physical location, space requirements, storage, and device availability within the therapy environment.	Dedicated area (yes/no); floor space; number of devices; transport time to therapy area.

Note: *n* (%) indicates studies with explicit reporting of the component in extracted service delivery/workflow descriptions; multiple components could be reported per study.

**Table 3:** Implementation outcomes and measures across included studies.

Outcome (Proctor)	Studies Reporting *n* (%)	Common Measures/Instruments	Common Operational Metrics	Predominant Evidence Basis
Acceptability	31 (56.4%)	System Usability Scale (SUS; *n* = 17), bespoke satisfaction surveys, interviews/focus groups.	System Usability Scale score; perceived usefulness/ease; qualitative themes on experience.	Quantitative (System Usability Scale/bespoke surveys) + qualitative (interviews/focus groups).
Feasibility	40 (72.7%)	Service logs, session completion rates, setup time tracking, clinician feedback.	Sessions delivered; completion rate; setup/donning time; adverse events during delivery.	Administrative/service logs + qualitative clinician feedback.
Adoption	23 (41.8%)	Utilization metrics (device/session logs), uptake by clinicians/sites.	Number of sessions; % eligible receiving robotics; device utilization rate.	Administrative/device utilization logs and uptake metrics.
Appropriateness	18 (32.7%)	Technology Acceptance Model (*n* = 2), Unified Theory of Acceptance and Use of Technology (*n* = 2), perceived fit/usefulness surveys and interviews.	Fit with workflow; perceived usefulness; suitability for pathway/patient selection.	Quantitative (technology acceptance/fit surveys) + qualitative.
Fidelity	8 (14.5%)	Protocol adherence checklists; minimum-dose thresholds; completion of planned elements.	% sessions meeting protocol; delivered dose vs. intended; deviation rates.	Checklist/protocol adherence metrics (administrative).
Penetration	8 (14.5%)	Service-level reach metrics; uptake across units; proportion eligible treated.	Proportion of eligible patients receiving sessions; spread across sites/teams.	Service reach metrics; often descriptive/administrative.
Sustainability	7 (12.7%)	Follow-up service audits; utilization over time; continuation after implementation period.	Utilization maintained at follow-up; continuation of pathway; staff retention of competencies.	Follow-up service audits/utilization over time; often descriptive.
Implementation cost	9 (16.4%)	Costing forms, time-driven staff costing, equipment/maintenance costs, budget impact.	Staff minutes/cost per session; equipment and maintenance cost; incremental cost per pathway.	Economic costing/time-driven costing/budget impact; often partial/descriptive.

Note: *n* (%) indicates studies in which the outcome was explicitly reported or mapped from an operationally matching source passage in the study-level extraction; explicit and inferred assignments are distinguished in Supplementary Table S5. SUS = System Usability Scale; TAM = Technology Acceptance Model; UTAUT = Unified Theory of Acceptance and Use of Technology.

**Table 4:** Workflow archetypes and minimum implementation metrics for routine-care rehabilitation robotics.

Workflow Archetype	Defining Service Configuration	Minimum Operational Metrics to Report
Embedded within standard PT/OT sessions	Robotics delivered within existing therapy slots; setup and calibration are absorbed into fixed session time.	Setup time; staff minutes/session; screening or progression criteria; throughput; documentation method.
Dedicated robotics slot or area	Robotics delivered as a scheduled, bookable service with defined roles for setup, supervision and referral.	Slot fill rate; utilization logs; role allocation; maintenance pathway; adverse-event reporting.
Circuit or multi-station robot gym	Multiple technologies are co-located and patients rotate between stations under coordinated or group supervision.	Staff-to-participant ratio; station time; repetitions or dose per station; scheduling rules; space requirements.
High-staffing gait/exoskeleton programme	Gait robotics or exoskeletons delivered through structured screening, monitoring, stop rules and higher supervision intensity.	Screening failure rate; adverse events per 1000 sessions; training hours; mean setup or donning time; escalation rules.
Clinic-to-home or community pathway	Robotics introduced in clinic with transition planning, follow-up, safety monitoring and technical support beyond the clinic.	Eligibility/transition criteria; adherence monitoring; documentation across settings; technical support response time; follow-up sustainment metrics.
Hub-and-spoke or networked scale-up	Centralized expertise supports multiple sites through shared protocols, training, governance and spread planning.	Number of adopting sites; protocol standardization; training coverage; penetration across units; maintenance and procurement governance.
Multi-technology programme with robotics as a core component	Robotics is integrated within a broader technology-assisted rehabilitation programme with programme-level governance.	Attribution rules; programme-level scheduling; cross-technology data capture; resource and time drivers; implementation cost elements.

Note: Archetypes were derived inductively from the included studies and are not mutually exclusive. They are evidence-informed, unvalidated service profiles rather than a clinical guideline or consensus recommendation.

## Data Availability

The review protocol is publicly available on the Open Science Framework (OSF): 10.17605/OSF.IO/96G83. Data extracted and analyzed in this review are included in the article and Supplementary Materials.

## Supplementary Materials

The following supporting information can be downloaded at: https://www.mdpi.com/article/10.3390/healthcare14162546/s1, Table S1. PRISMA-ScR checklist; Table S2. Methods, transparency and eligibility decision rules; Table S3. Study-level extraction and framework mapping; Table S4. Translational readiness signals; Table S5. Explicit versus inferred coding; Table S6. Report-level design-to-appraisal crosswalk and instrument allocation; Table S7. Study-level design-appropriate appraisal results; Table S8. Subgroup mapping; Table S9. Determinant–strategy–outcome matrix; Table S10. Prospective minimum reporting set. Table S2 is presented as seven linked methodological sub-tables (Table S2A–G).

## Artificial Intelligence Use Statement

During manuscript preparation, the authors used ChatGPT (version GPT-5.6, OpenAI, San Francisco, CA, USA) to support English-language refinement, grammar correction, stylistic polishing, formatting consistency, and graphical layout optimization. The tool was not used to generate the research question, define eligibility criteria, design the methodology, perform literature searches, screen records, extract data, code studies, conduct analyses, interpret findings, or draw conclusions. No generative tool contributed scientific content to the appraisal figures or Supplementary Materials; all appraisal judgments were made by the authors from the source articles. All scientific content, methodological decisions, data extraction, framework mapping, synthesis, interpretation, and final wording were independently reviewed, edited, and verified by the authors. The authors take full responsibility for the accuracy, originality, integrity, and final content of the manuscript.
