# Supplementary material for: Implementing Rehabilitation Robotics in Routine Care: A Mapping Review of Service Delivery Models, Implementation Determinants, Strategies, and Real-World Outcomes
Source: Healthcare (Basel). 2026 Aug 14;14(16):2546. doi: 10.3390/healthcare14162546 (PMC13512581; doi:10.3390/healthcare14162546)
Supplement: Supplementary file 1 [file healthcare-14-02546-s001.zip › healthcare-4454764-supplementary.pdf]

Supplementary Material

This supplementary file comprises ten numbered supplementary tables.

Supplementary Table S1. PRISMA-ScR checklist

SECTION	ITEM	PRISMA-ScR CHECKLIST ITEM	REPORTED ON PAGE #
TITLE	TITLE	TITLE	TITLE
Title	1	Identify the report as a scoping review.	p. 1
ABSTRACT	ABSTRACT	ABSTRACT	ABSTRACT
Structured summary	2	Provide a structured summary that includes (as applicable): background, objectives, eligibility criteria, sources of evidence, charting methods, results, and conclusions that relate to the review questions and objectives.	p. 1
INTRODUCTION	INTRODUCTION	INTRODUCTION	INTRODUCTION
Rationale	3	Describe the rationale for the review in the context of what is already known. Explain why the review questions/objectives lend themselves to a scoping review approach.	pp. 2–3
Objectives	4	Provide an explicit statement of the questions and objectives being addressed with reference to their key elements (e.g., population or participants, concepts, and context) or other relevant key elements used to conceptualize the review questions and/or objectives.	p. 3
METHODS	METHODS	METHODS	METHODS
Protocol and registration	5	Indicate whether a review protocol exists; state if and where it can be accessed (e.g., a Web address); and if available, provide registration information, including the registration number.	p. 3
Eligibility criteria	6	Specify characteristics of the sources of evidence used as eligibility criteria (e.g., years considered, language, and publication status), and provide a rationale.	pp. 3–5; Supplementary Tables S2A and S2G
Information sources*	7	Describe all information sources in the search (e.g., databases with dates of coverage and contact with authors to identify additional sources), as well as the date the most recent search was executed.	p. 5
Search	8	Present the full electronic search strategy for at least 1 database, including any limits used, such that it could be repeated.	p. 5; Supplementary Tables S2B–S2D
Selection of sources of evidence†	9	State the process for selecting sources of evidence (i.e., screening and eligibility) included in the scoping review.	p. 5; Supplementary Tables S2E and S2G
Data charting process‡	10	Describe the methods of charting data from the included sources of evidence (e.g., calibrated forms or forms that have been tested by the team before their use, and whether data charting was done independently or in duplicate) and any processes for obtaining and confirming data from investigators.	pp. 5–6; Supplementary Table S2F
Data items	11	List and define all variables for which data were sought and any assumptions and simplifications made.	pp. 5–6; Supplementary Table S3
Critical appraisal of individual sources of evidence§	12	If done, provide a rationale for conducting a critical appraisal of included sources of evidence; describe the methods used and how this information was used in any data synthesis (if appropriate).	p. 6; Supplementary Tables S6–S7
Synthesis of results	13	Describe the methods of handling and summarizing the data that were charted.	p. 6
RESULTS	RESULTS	RESULTS	RESULTS
Selection of sources of evidence	14	Give numbers of sources of evidence screened, assessed for eligibility, and included in the review, with reasons for exclusions at each stage, ideally using a flow diagram.	p. 7; Figure 1
Characteristics of sources of evidence	15	For each source of evidence, present characteristics for which data were charted and provide the citations.	pp. 8–9; Table 1; Supplementary Table S3
Critical appraisal within sources of evidence	16	If done, present data on critical appraisal of included sources of evidence (see item 12).	pp. 17–18; Figures 5–6; Supplementary Tables S6–S7
Results of individual sources of evidence	17	For each included source of evidence, present the relevant data that were charted that relate to the review questions and objectives.	pp. 7–18; Tables 1–4; Supplementary Tables S3–S10
Synthesis of results	18	Summarize and/or present the charting results as they relate to the review questions and objectives.	pp. 7–18
DISCUSSION	DISCUSSION	DISCUSSION	DISCUSSION
Summary of evidence	19	Summarize the main results (including an overview of concepts, themes, and types of evidence available), link to the review questions and objectives, and consider the relevance to key groups.	pp. 19–22
Limitations	20	Discuss the limitations of the scoping review process.	p. 22
Conclusions	21	Provide a general interpretation of the results with respect to the review questions and objectives, as well as potential implications and/or next steps.	p. 22
FUNDING	FUNDING	FUNDING	FUNDING
Funding	22	Describe sources of funding for the included sources of evidence, as well as sources of funding for the scoping review. Describe the role of the funders of the scoping review.	p. 23

Note. The checklist documents reporting against PRISMA-ScR; page numbers refer to the manuscript.
Abbreviations: JBI, Joanna Briggs Institute; PRISMA-ScR, Preferred Reporting Items for Systematic reviews and Meta-Analyses extension for Scoping Reviews.

Supplementary Table S2A. Methods transparency and eligibility decision rules

Item	Operational detail	Methodological rationale
Review type and purpose	Mapping review of implementation determinants, strategies, outcomes, service-delivery models, workflows and resource signals for RR in routine or service-linked care.	Appropriate for heterogeneous evidence mapping without pooled effect-size synthesis.
Protocol and registration	OSF-registered protocol: 10.17605/OSF.IO/96G83; project and initial protocol registered 14 February 2026; version 1.1 uploaded 19 February 2026 before database search execution on 21 February 2026. The deposited protocol contained three cross-database core search strings without field tags; native database-interface search histories were not retained.	Documents prospective registration and distinguishes deposited core strings from native database-interface histories, which were not retained.
Reporting standards	JBI guidance relevant to mapping/scoping reviews; reporting according to PRISMA-ScR; PRISMA-ScR flow diagram.	Uses one consistent review description and reporting standard.
Information sources	PubMed/MEDLINE (biomedical/clinical); Embase (complementary biomedical, device and European indexing); Web of Science Core Collection and Scopus (multidisciplinary/citation coverage); Cochrane Library (structured trial/evidence records); and CINAHL Complete searched through EBSCOhost (allied-health and rehabilitation coverage). Database roles, interfaces, original search date and preserved source-level yields are detailed in Supplementary Table S2B.	Explains source complementarity and identifies the database and interface needed to reproduce the historical search.
Search concepts	Rehabilitation robotics; routine care/clinical practice; implementation/integration; workflow/service delivery; determinants, strategies, feasibility, acceptability, and usability.	Maximizes implementation-relevant retrieval rather than purely clinical efficacy retrieval.
Language and source limits	English and Italian full texts; no publication-year restriction; no stand-alone grey literature search; no citation chasing. No otherwise eligible record was excluded solely on language grounds.	Maintains a reproducible database-indexed sampling frame while acknowledging possible omissions and explaining why no language-exclusion count appears.
PCC population	People receiving rehabilitation of any age and implementation stakeholders, including clinicians, managers, technical staff, patients, and caregivers.	Captures patient-, clinician-, service-, and system-level implementation evidence.
PCC concept	Integration of powered rehabilitation robots into care pathways, including workflows, determinants, strategies, outcomes, and resource-use signals.	Keeps the focus on implementation content rather than device efficacy alone.
PCC context	Inpatient, outpatient, community, home, mixed, and system-level routine or service-linked care.	Recognizes routine care as a service configuration rather than a single setting.
Inclusion rule	Full-text empirical, service, quality-improvement, pragmatic/hybrid, mixed-methods, qualitative, survey, and economic studies with extractable implementation-relevant content.	Ensures evidence is auditable and extractable.
Exclusion rule	Reviews, protocols without results, editorials, abstract-only records, laboratory-only studies, assessment-only robotics, non-rehabilitation robotics, and efficacy-only reports without implementation content.	Prevents misattribution of clinical efficacy evidence as implementation evidence.
Screening and calibration	Independent screening by A.C. and R.S.C.; 4,546 title/abstract records and 107 full-text reports were assessed in duplicate; disagreements were resolved by discussion with A.Q. available for adjudication; κ = 0.71 and κ = 0.75, respectively.	Documents duplicate-screening denominators, consistency and decision safeguards.
Data charting	Piloted extraction in Microsoft Excel; R.S.C. charted and A.C. verified all fields for all 55 included reports. Because this was verification rather than independent duplicate charting, no formal agreement coefficient was calculated.	Provides a source-linked verification step and states the boundary of the charting-agreement assessment.
Framework coding	CFIR, ERIC and Proctor mapping used prespecified construct definitions. Verbatim source descriptions were linked to codes; explicit and inferred assignments were flagged; overlapping constructs required distinct meanings; ambiguous passages were narrowed or left unmapped; differences identified during verification were resolved against the decision rules.	Reduces subjectivity and makes interpretive framework mapping transparent and auditable.
Synthesis logic	Descriptive counts, matrices, subgroup mapping, and determinant-strategy-outcome co-reporting; no meta-analysis.	Avoids overinterpreting frequency as causal evidence.
Risk-of-bias/appraisal use	Each of 55 reports was classified and appraised once: RoB 2 (n=2), ROBINS-I (n=4), JBI quasi-experimental (n=11), JBI case series (n=8), JBI analytical cross-sectional (n=6), JBI cohort (n=2), CASP qualitative (n=11), MMAT (n=4), QI-MQCS (n=3), CHEERS 2022 + ISPOR (n=4). Appraisal was completed by Rocco Salvatore Calabrò (R.S.C.) and verified by Andrea Calderone (A.C.); disagreements were resolved by consensus, with Angelo Quartarone (A.Q.) acting as adjudicator when required. The six author-team reports were handled as follows: each report was appraised exclusively by authors who were not involved in the corresponding source article.	Optional contextual appraisal; no exclusion, quantitative weighting or causal inference. CHEERS assesses reporting completeness.

Note. Eligibility, screening, coding, appraisal, and synthesis procedures are stated as applied in the review; documented protocol clarifications are identified explicitly.

Abbreviations: A.C., Andrea Calderone; A.Q., Angelo Quartarone; CASP, Critical Appraisal Skills Programme; CFIR, Consolidated Framework for Implementation Research; CHEERS, Consolidated Health Economic Evaluation Reporting Standards; ERIC, Expert Recommendations for Implementing Change; ISPOR, International Society for Pharmacoeconomics and Outcomes Research; JBI, Joanna Briggs Institute; MMAT, Mixed Methods Appraisal Tool; PCC, Population, Concept, Context; PRISMA-ScR, Preferred Reporting Items for Systematic reviews and Meta-Analyses extension for Scoping Reviews; QI-MQCS, Quality Improvement Minimum Quality Criteria Set; R.S.C., Rocco Salvatore Calabrò; RoB 2, Risk of Bias 2; ROBINS-I, Risk Of Bias In Non-randomized Studies of Interventions.

Supplementary Table S2B. Databases, interfaces, search coverage and historical source-level yields

Database	Interface	Coverage role	Original search date	String 1, n	String 2, n	String 3, n	Source total, n
PubMed/MEDLINE	PubMed	Biomedical and clinical literature	21 February 2026	42	6	24	72
Embase	Embase	Biomedical, device and European indexing	21 February 2026	58	9	84	151
Web of Science Core Collection	Web of Science	Multidisciplinary and citation-indexed literature	21 February 2026	35	17	1,275	1,327
Scopus	Scopus	Multidisciplinary, engineering and health-services coverage	21 February 2026	1,421	1,135	1,031	3,587
Cochrane Library	Cochrane Library	CENTRAL and structured trial/evidence records	21 February 2026	10	4	21	35
CINAHL Complete	EBSCOhost	Allied-health and rehabilitation literature	21 February 2026	13	Not run	8	21
**All sources**	—	**Records before cross-source de-duplication**	**21 February 2026**	**1,579**	**1,171**	**2,443**	**5,193**

Note. Counts are the records contained in the preserved historical exports before cross-source de-duplication. The Web of Science String 3 set was exported in two batches containing 1,000 and 275 records; the second file contains 276 spreadsheet rows because its header occupies the first row. CINAHL Complete was searched through EBSCOhost; String 2 was not run on this interface. No updated search was undertaken after the prespecified search date.

Abbreviations: CINAHL, Cumulative Index to Nursing and Allied Health Literature; CENTRAL, Cochrane Central Register of Controlled Trials.

Supplementary Table S2C. OSF-deposited cross-database core strings and historical query-level yields

Database/interface	Search string	OSF-deposited cross-database core string (verbatim)	Records, n
PubMed/MEDLINE	String 1	("robot assisted rehabilitation" OR "rehabilitation robot" OR exoskeleton OR Lokomat OR Ekso OR InMotion) AND (implementation OR integration OR adoption) AND ("routine care" OR "clinical practice")	42
PubMed/MEDLINE	String 2	(Lokomat OR Ekso OR ReWalk OR Indego OR HAL OR Andago OR InMotion OR "MIT-Manus" OR "G-EO" OR REX OR Myosuit) AND ("spinal cord injury" OR SCI OR "neurodegenerative disease" OR "neurodegenerative disorder" OR "Parkinson disease" OR "multiple sclerosis" OR "amyotrophic lateral sclerosis" OR "motor neuron disease") AND (implementation OR integration OR adoption OR "routine care" OR "clinical practice") AND (usability OR acceptability OR "System Usability Scale" OR SUS)	6
PubMed/MEDLINE	String 3	("robot assisted rehabilitation" OR "rehabilitation robot" OR exoskeleton OR Lokomat OR Ekso OR InMotion) AND (rehabilitation OR neurorehabilitation OR stroke) AND (barriers OR facilitators OR feasibility OR usability OR acceptability)	24
Embase	String 1	("robot assisted rehabilitation" OR "rehabilitation robot" OR exoskeleton OR Lokomat OR Ekso OR InMotion) AND (implementation OR integration OR adoption) AND ("routine care" OR "clinical practice")	58
Embase	String 2	(Lokomat OR Ekso OR ReWalk OR Indego OR HAL OR Andago OR InMotion OR "MIT-Manus" OR "G-EO" OR REX OR Myosuit) AND ("spinal cord injury" OR SCI OR "neurodegenerative disease" OR "neurodegenerative disorder" OR "Parkinson disease" OR "multiple sclerosis" OR "amyotrophic lateral sclerosis" OR "motor neuron disease") AND (implementation OR integration OR adoption OR "routine care" OR "clinical practice") AND (usability OR acceptability OR "System Usability Scale" OR SUS)	9
Embase	String 3	("robot assisted rehabilitation" OR "rehabilitation robot" OR exoskeleton OR Lokomat OR Ekso OR InMotion) AND (rehabilitation OR neurorehabilitation OR stroke) AND (barriers OR facilitators OR feasibility OR usability OR acceptability)	84
Web of Science Core Collection	String 1	("robot assisted rehabilitation" OR "rehabilitation robot" OR exoskeleton OR Lokomat OR Ekso OR InMotion) AND (implementation OR integration OR adoption) AND ("routine care" OR "clinical practice")	35
Web of Science Core Collection	String 2	(Lokomat OR Ekso OR ReWalk OR Indego OR HAL OR Andago OR InMotion OR "MIT-Manus" OR "G-EO" OR REX OR Myosuit) AND ("spinal cord injury" OR SCI OR "neurodegenerative disease" OR "neurodegenerative disorder" OR "Parkinson disease" OR "multiple sclerosis" OR "amyotrophic lateral sclerosis" OR "motor neuron disease") AND (implementation OR integration OR adoption OR "routine care" OR "clinical practice") AND (usability OR acceptability OR "System Usability Scale" OR SUS)	17
Web of Science Core Collection	String 3	("robot assisted rehabilitation" OR "rehabilitation robot" OR exoskeleton OR Lokomat OR Ekso OR InMotion) AND (rehabilitation OR neurorehabilitation OR stroke) AND (barriers OR facilitators OR feasibility OR usability OR acceptability)	1,275
Scopus	String 1	("robot assisted rehabilitation" OR "rehabilitation robot" OR exoskeleton OR Lokomat OR Ekso OR InMotion) AND (implementation OR integration OR adoption) AND ("routine care" OR "clinical practice")	1,421
Scopus	String 2	(Lokomat OR Ekso OR ReWalk OR Indego OR HAL OR Andago OR InMotion OR "MIT-Manus" OR "G-EO" OR REX OR Myosuit) AND ("spinal cord injury" OR SCI OR "neurodegenerative disease" OR "neurodegenerative disorder" OR "Parkinson disease" OR "multiple sclerosis" OR "amyotrophic lateral sclerosis" OR "motor neuron disease") AND (implementation OR integration OR adoption OR "routine care" OR "clinical practice") AND (usability OR acceptability OR "System Usability Scale" OR SUS)	1,135
Scopus	String 3	("robot assisted rehabilitation" OR "rehabilitation robot" OR exoskeleton OR Lokomat OR Ekso OR InMotion) AND (rehabilitation OR neurorehabilitation OR stroke) AND (barriers OR facilitators OR feasibility OR usability OR acceptability)	1,031
Cochrane Library	String 1	("robot assisted rehabilitation" OR "rehabilitation robot" OR exoskeleton OR Lokomat OR Ekso OR InMotion) AND (implementation OR integration OR adoption) AND ("routine care" OR "clinical practice")	10
Cochrane Library	String 2	(Lokomat OR Ekso OR ReWalk OR Indego OR HAL OR Andago OR InMotion OR "MIT-Manus" OR "G-EO" OR REX OR Myosuit) AND ("spinal cord injury" OR SCI OR "neurodegenerative disease" OR "neurodegenerative disorder" OR "Parkinson disease" OR "multiple sclerosis" OR "amyotrophic lateral sclerosis" OR "motor neuron disease") AND (implementation OR integration OR adoption OR "routine care" OR "clinical practice") AND (usability OR acceptability OR "System Usability Scale" OR SUS)	4
Cochrane Library	String 3	("robot assisted rehabilitation" OR "rehabilitation robot" OR exoskeleton OR Lokomat OR Ekso OR InMotion) AND (rehabilitation OR neurorehabilitation OR stroke) AND (barriers OR facilitators OR feasibility OR usability OR acceptability)	21
CINAHL Complete via EBSCOhost	String 1	("robot assisted rehabilitation" OR "rehabilitation robot" OR exoskeleton OR Lokomat OR Ekso OR InMotion) AND (implementation OR integration OR adoption) AND ("routine care" OR "clinical practice")	13
CINAHL Complete via EBSCOhost	String 2	(Lokomat OR Ekso OR ReWalk OR Indego OR HAL OR Andago OR InMotion OR "MIT-Manus" OR "G-EO" OR REX OR Myosuit) AND ("spinal cord injury" OR SCI OR "neurodegenerative disease" OR "neurodegenerative disorder" OR "Parkinson disease" OR "multiple sclerosis" OR "amyotrophic lateral sclerosis" OR "motor neuron disease") AND (implementation OR integration OR adoption OR "routine care" OR "clinical practice") AND (usability OR acceptability OR "System Usability Scale" OR SUS)	0
CINAHL Complete via EBSCOhost	String 3	("robot assisted rehabilitation" OR "rehabilitation robot" OR exoskeleton OR Lokomat OR Ekso OR InMotion) AND (rehabilitation OR neurorehabilitation OR stroke) AND (barriers OR facilitators OR feasibility OR usability OR acceptability)	8

Note. The table reproduces verbatim the three cross-database core strings without field tags that were deposited with the OSF protocol registered on 14 February 2026 and updated as version 1.1 on 19 February 2026. The accompanying counts are the preserved historical query-level yields for the searches completed on 21 February 2026. Native database-interface search histories and exact executed interface syntax were not retained; this table therefore does not purport to reproduce database-specific syntax. Searches covered database inception to the search date, with no publication-year or study-design filter. English- and Italian-language eligibility was applied during screening when an equivalent platform filter was unavailable; no otherwise eligible record was excluded solely on language grounds. Counts reflect query yields before cross-source de-duplication and therefore include overlap between strings and databases. No retrospective rerun or updated search was performed.

Abbreviations: CINAHL, Cumulative Index to Nursing and Allied Health Literature; EBSCOhost, database access platform used for CINAHL Complete; HAL, Hybrid Assistive Limb; SCI, spinal cord injury; SUS, System Usability Scale.

Supplementary Table S2D. Search-strategy development and PRESS-informed internal validation checks

Component	Reproducibility criterion	Applied procedure and retained record	Transparency boundary
Search scope	Identify all information sources, interfaces, coverage dates and limits.	PubMed/MEDLINE, Embase, Web of Science Core Collection, Scopus, Cochrane Library and CINAHL Complete through EBSCOhost were searched from inception to 21 February 2026. No publication-year or study-design filter was applied.	English- and Italian-language eligibility was applied in the platform where available and otherwise during screening.
Concept structure	State the conceptual blocks and how they were combined.	Three prespecified strings combined rehabilitation robotics and named devices with routine-care, implementation, integration, workflow, determinant, feasibility, acceptability, usability and related service concepts.	The three strings were complementary retrieval routes and were combined before cross-source de-duplication.
Platform translation	Adapt controlled vocabulary, free text and field syntax to each database.	The protocol specified database adaptation of controlled vocabulary, free text and field syntax. The retained record comprises the three no-field-tag core strings, historical query-level yields and source totals. String 2 was not run in CINAHL Complete.	Native database-interface strings and search histories were not retained; Supplementary Table S2C therefore reproduces the deposited core strings rather than reconstructed platform syntax.
Boolean and syntax check	Check parentheses, phrase marks, operators, field tags and concept-block balance.	A.C. and R.S.C. completed an internal PRESS-informed review before execution and resolved minor translation, grouping and field-tag inconsistencies.	This was an internal methodological check rather than an independent external information-specialist review.
Limits and filters	Apply limits consistently and document where platform behavior differs.	No date or design limits were used. Language limits were applied when supported by the platform; otherwise language eligibility was assessed during screening.	No stand-alone grey-literature search or backward/forward citation chasing was performed.
Run documentation	Retain the historical execution date, query-level yield and source-level total.	The original execution date and available query-level yields were checked against source totals of 72, 151, 1,327, 3,587, 35 and 21 records, respectively.	The six source totals sum to the 5,193 records reported in the Results and PRISMA-ScR flow diagram.
Archive limitation	Distinguish preserved evidence from reconstructed documentation.	The OSF protocol retained the three cross-database core strings, and historical exports and yields were preserved; the native database-interface search histories and exact executed syntax were not retained.	No updated search or retrospective rerun was undertaken after the prespecified search date.

Note. Internal checks were completed before the historical search execution. This table distinguishes the retained records from native database-interface syntax that was not preserved and does not imply an independent external PRESS review or a subsequent search update.

Abbreviations: A.C., Andrea Calderone; CINAHL, Cumulative Index to Nursing and Allied Health Literature; EBSCOhost, database access platform; MeSH, Medical Subject Headings; PRESS, Peer Review of Electronic Search Strategies; R.S.C., Rocco Salvatore Calabrò.

Supplementary Table S2E. Study-selection workflow and quality controls

Selection component	Applied procedure	Quality-control record
Record aggregation	Records from the six sources and three retrieval strings were collated before cross-source de-duplication.	The preserved source totals matched the 5,193 identified records.
De-duplication	A structured duplicate-removal workflow was applied before screening.	A total of 647 duplicate records were removed, leaving 4,546 unique records for title/abstract screening.
Calibration	Eligibility definitions and borderline examples were reviewed before formal screening to harmonize interpretation of robotics centrality, routine-care linkage and implementation-content thresholds.	The same operational rules were used at title/abstract and full-text stages; borderline rules are documented in Supplementary Table S2G.
Screening platform and stages	Title/abstract screening and full-text assessment were conducted in Rayyan as two sequential stages.	The software and stage structure match the methods reported in Section 2.3 of the manuscript.
Independent assessment	A.C. and R.S.C. screened records independently against the predefined population, technology, context and implementation-content criteria.	Candidate records were not retained or excluded on the judgment of one reviewer alone.
Conflict resolution	Disagreements were resolved by discussion, with A.Q. available for adjudication when required.	Full-text exclusion reasons were retained to support auditability.
Agreement	Inter-reviewer agreement was quantified with Cohen's kappa.	$\kappa = 0.71$ across 4,546 title/abstract records and $\kappa = 0.75$ across 107 full-text reports.
PRISMA-ScR flow consistency check	Stage totals were checked arithmetically against the final flow diagram and Results.	4,546 screened − 4,428 excluded = 118 sought; 118 − 11 not retrieved = 107 assessed; 107 − 52 excluded = 55 included. No otherwise eligible record was excluded solely on language grounds.

Note. Counts are identical to the manuscript Results and Figure 1. Arithmetic is displayed to make the transition between identification, screening, eligibility and inclusion independently checkable.

Abbreviations: A.C., Andrea Calderone; A.Q., Angelo Quartarone; PRISMA-ScR, Preferred Reporting Items for Systematic reviews and Meta-Analyses extension for Scoping Reviews; R.S.C., Rocco Salvatore Calabrò.

Supplementary Table S2F. Data-charting and framework-coding safeguards

Methodological component	Applied procedure	Safeguard against error or subjectivity	Linked output
Charting-form development	A prespecified Microsoft Excel form was piloted before full extraction.	The pilot was used to clarify field definitions and the level of detail required for heterogeneous study designs.	Supplementary Tables S3-S4
Charting responsibilities	R.S.C. charted the study-level data and A.C. verified all fields against the source report.	Second-reviewer verification was applied to every included report rather than a sample. Because charting was verified rather than independently duplicated, no formal agreement coefficient was calculated.	Supplementary Tables S3-S4
Core data domains	Bibliographic data, country, setting, population, device, service pathway, staffing, training, workflow, safety, documentation, maintenance, infrastructure, determinants, strategies, outcomes and resource information were charted when available.	Operational fields were retained at study level so aggregate counts could be traced to individual reports.	Supplementary Tables S3-S5
Framework assignment	Determinants were mapped to CFIR, strategies to ERIC and implementation outcomes to Proctor's taxonomy.	Prespecified construct definitions were applied consistently across designs and settings.	Supplementary Table S5
Explicit versus inferred coding	Author terminology was retained when explicit; otherwise a code was assigned only when a source passage operationally matched the construct.	Every assignment retained an explicit/inferred flag and remained linked to the source description.	Supplementary Table S5
Overlapping constructs	More than one construct was assigned to a passage only when the passage expressed distinct implementation meanings.	Ambiguous overlap was resolved with the narrower supported construct or left unmapped.	Supplementary Table S5
Coding disagreements	Differences identified during verification were discussed against the predefined decision rules.	Unresolved ambiguity was not converted into a broader or more favorable code.	Supplementary Tables S2G and S5
Missing or non-extractable information	Absent information was retained as not reported, unclear or not extractable rather than imputed.	Reporting presence was separated from practical importance and from evidence of causal influence.	Supplementary Tables S3-S10
Appraisal linkage	Each included report was classified by actual design and assigned once to one primary appraisal instrument. Appraisal was completed by Rocco Salvatore Calabrò (R.S.C.) and verified by Andrea Calderone (A.C.); disagreements were resolved by consensus, with Angelo Quartarone (A.Q.) acting as adjudicator when required. The six author-team reports were handled as follows: each report was appraised exclusively by authors who were not involved in the corresponding source article.	Appraisal judgments were linked to report-specific rationales, used the included report rather than a parent or companion article as the unit, and were not pooled across tools.	Supplementary Tables S6-S7

Note. The table records the procedures used to limit extraction error and interpretive subjectivity. It does not convert framework mapping or appraisal into causal evidence or a common certainty score.

Abbreviations: A.C., Andrea Calderone; A.Q., Angelo Quartarone; CFIR, Consolidated Framework for Implementation Research; ERIC, Expert Recommendations for Implementing Change; R.S.C., Rocco Salvatore Calabrò.

Supplementary Table S2G. Borderline eligibility and coding decision-rule register

Decision point	Operational rule	Applied stage	Rationale
Robotics centrality	Include powered robotic devices used to deliver, assist or augment therapeutic movement training; exclude non-rehabilitation, diagnostic and assessment-only robotics without a therapeutic pathway.	Screening	Keeps the technology of interest central to the evidence source.
Routine-care linkage	Include delivery in clinical services or an explicit link to procurement, rollout, scale-up, sustainment or a service pathway; exclude laboratory-only work without such linkage.	Screening	Aligns inclusion with the health-service question.
Implementation-content threshold	Require at least one extractable determinant, strategy, implementation outcome, service configuration, workflow or resource-use signal beyond generic statements.	Screening	Prevents efficacy-only evidence from being treated as implementation evidence.
Therapeutic versus daily-mobility use	Include wearable or assistive devices when embedded in structured therapeutic training; exclude daily-mobility use without a rehabilitation program.	Full text	Maintains a consistent therapeutic rehabilitation scope.
Mixed interventions	Include a multi-component pathway only when the robotic component is core and implementation findings can be attributed to it.	Full text/charting	Avoids attributing system-level findings to robotics when the contribution is inseparable.
Overlapping reports	Retain the most complete report as the included unit and use companion publications only to clarify implementation details.	Screening/charti ng	Avoids double counting while preserving relevant context.
Language and availability	Include retrievable English or Italian full texts; record non-retrieval and language exclusions at the appropriate selection stage.	Full text	Makes the accessible sampling frame explicit.
Grey literature	Do not undertake a stand-alone grey-literature search; exclude grey literature encountered in the database set and record the full-text reason.	Search/full text	Maintains the stated database-indexed sampling frame.
Framework inference	Infer a CFIR, ERIC or Proctor code only when a source passage provides an operationally matching barrier, facilitator, action or outcome.	Charting	Limits reviewer-derived coding to source-supported content.
Ambiguous or missing evidence	Use the narrowest supported code or leave the passage unmapped; classify absent items as not reported, unclear or not extractable.	Charting	Avoids overcoding and undocumented imputation.
Frequency interpretation	Count reporting presence using 55 included reports as the denominator unless otherwise stated; do not interpret frequency as practical importance, comparative effect or causal influence.	Synthesis	Preserves the descriptive purpose of the mapping review.

Note. These operational rules were applied to maintain consistent boundaries across screening, charting and synthesis. Decisions were source-linked and were not used to favor studies from a particular country, author group or device program.

Abbreviations: CFIR, Consolidated Framework for Implementation Research; ERIC, Expert Recommendations for Implementing Change; RR, rehabilitation robotics.

Supplementary Table S3. Study-level extraction and framework mapping

Study	Setting, design, and implementation stage	Robotic technology and service configuration	Implementation evidence extracted
Cao et al., 2021 [46]	Setting: Acute inpatient stroke rehabilitation (IRF; 26-bed unit). Design: Quality-improvement initiative. Implementation stage: Early rollout; evidence unit: Device pathway.	Technology: Gait robotics. Service configuration: device pathway.	Documented domains: staffing and supervision, training and competency, workflow and scheduling, safety governance, utilization and monitoring, and cost and resource use. Principal bottleneck: setup/workflow burden and staff confidence. Enabling conditions: structured training, written procedures, and designated clinical support.
Flynn et al., 2025 [47]	Setting: Inpatient rehabilitation facility (mixed rehab; stroke), Queensland. Design: Qualitative focus-group study with embedded usability survey. Implementation stage: Routine use; evidence unit: Device pathway.	Technology: Upper-limb robotics. Service configuration: device pathway.	Documented domains: staffing and supervision, training and competency, workflow and scheduling, and utilization and monitoring. Principal bottleneck: setup/workflow burden and staff confidence. Enabling conditions: structured training, written procedures, and designated clinical support.
Gillespie et al., 2023 [48]	Setting: Inpatient rehabilitation after neurological injury (stroke, SCI, TBI). Design: Retrospective descriptive case series with therapist survey. Implementation stage: Routine use; evidence unit: Device pathway.	Technology: Overground wearable robotic exoskeletons for gait. Service configuration: device pathway.	Documented domains: staffing and supervision, training and competency, workflow and scheduling, eligibility and patient selection, safety governance, and utilization and monitoring. Principal bottleneck: staffing and staff confidence. Enabling conditions: standardized eligibility screening, written protocols, and progression criteria.
Heinemann et al., 2018 [49]	Setting: Four SCI Model Systems centers. Design: Multicentre qualitative focus-group study. Implementation stage: Early rollout; evidence unit: Cross-centre programme.	Technology: Overground robotic exoskeletons for assisted walking. Service configuration: cross-centre programme.	Documented domains: staffing and supervision, training and competency, workflow and scheduling, eligibility and patient selection, safety governance, utilization and monitoring, cost and resource use, and network or system configuration. Principal bottleneck: cost/funding constraints and staff confidence. Enabling conditions: structured training, written procedures, and designated clinical support.
Pila et al., 2022 [50]	Setting: Inpatient neurorehabilitation unit (subacute stroke). Design: Retrospective non-randomized comparative intervention study. Implementation stage: Routine use; evidence unit: Device pathway.	Technology: Upper-limb end-effector robotic therapy. Service configuration: device pathway.	Documented domains: staffing and supervision, training and competency, workflow and scheduling, utilization and monitoring, and cost and resource use. Principal bottleneck: staff confidence and setup/workflow burden. Enabling conditions: routine utilization metrics, feedback, and service-level monitoring.
Read et al., 2020 [51]	Setting: Neurological rehabilitation hospital (inpatient and outpatient). Design: Exploratory qualitative interview study. Implementation stage: Early rollout; evidence unit: Continuum pathway.	Technology: Overground wearable robotic exoskeleton for gait. Service configuration: continuum pathway.	Documented domains: staffing and supervision, training and competency, workflow and scheduling, eligibility and patient selection, safety governance, and network or system configuration. Principal bottleneck: eligibility/tolerability and staff confidence. Enabling conditions: structured training, written procedures, and designated clinical support.
Warmbein et al., 2023 [52]	Setting: Inpatient care contexts (e.g., acute care and early rehabilitation). Design: Exploratory qualitative interview study. Implementation stage: Mixed phase; evidence unit: Design insights.	Technology: Robotic systems for assisted mobilization/movement. Service configuration: design insights.	Documented domains: staffing and supervision, training and competency, workflow and scheduling, safety governance, and cost and resource use. Principal bottleneck: staff confidence and cost/funding constraints. Enabling conditions: workflow redesign, protected device time, and written operating procedures.
Buschfort et al., 2010 [53]	Setting: Inpatient stroke rehabilitation (subacute and chronic). Design: Open single-group pre-post study. Implementation stage: Early rollout; evidence unit: Dedicated robotic area.	Technology: Device-assisted upper limb rehabilitation. Service configuration: dedicated robotic area.	Documented domains: staffing and supervision, training and competency, workflow and scheduling, eligibility and patient selection, safety governance, and utilization and monitoring. Principal bottleneck: staffing intensity and cost/funding constraints. Enabling conditions: group or semi-supervised delivery with explicit staff responsibilities.
Aprile et al., 2019 [54]	Setting: Two Italian rehabilitation centres. Design: Single-group feasibility study. Implementation stage: Early rollout; evidence unit: Multidevice pathway.	Technology: Upper-limb robotics and sensor-based technologies. Service configuration: multidevice pathway.	Documented domains: staffing and supervision, training and competency, workflow and scheduling, eligibility and patient selection, safety governance, and network or system configuration. Principal bottleneck: staffing and infrastructure. Enabling conditions: workflow redesign, protected device time, and written operating procedures.
Aprile et al., 2019 [55]	Setting: Dedicated robot-assisted rehabilitation area within an Italian rehabilitation hospital. Design: Observational single-group feasibility study. Implementation stage: Routine use; evidence unit: Dedicated robotic area.	Technology: Upper-limb robotics and sensor-based technologies. Service configuration: dedicated robotic area.	Documented domains: staffing and supervision, training and competency, workflow and scheduling, and utilization and monitoring. Principal bottleneck: staffing and staff confidence. Enabling conditions: workflow redesign, protected device time, and written operating procedures.
Gagnon et al., 2018 [56]	Setting: Community-based programme for manual wheelchair users with chronic spinal cord injury. Design: Prospective single-group feasibility case series. Implementation stage: Routine use; evidence unit: Device pathway.	Technology: Overground wearable robotic exoskeleton for locomotor. Service configuration: device pathway.	Documented domains: staffing and supervision, training and competency, workflow and scheduling, eligibility and patient selection, safety governance, and utilization and monitoring. Principal bottleneck: eligibility and community logistics. Enabling conditions: structured training, written procedures, and designated clinical support.
Gower et al., 2024 [57]	Setting: Italian rehabilitation hospital real-world data case study. Design: Real-world cost-minimization analysis. Implementation stage: Routine use; evidence unit: Org. cost model.	Technology: Upper-limb robotics and sensor-based technologies. Service configuration: org. cost model.	Documented domains: staffing and supervision, workflow and scheduling, utilization and monitoring, and cost and resource use. Principal bottleneck: staffing intensity and setup/workflow burden. Enabling conditions: group or semi-supervised delivery with explicit staff responsibilities.
Gower et al., 2025 [58]	Setting: Economic evaluation comparing alternative organisational models for upper-limb rehabilitation robotics. Design: Comparative economic evaluation across service models. Implementation stage: Scale-out; evidence unit: Org. cost model.	Technology: Upper-limb rehabilitation robotics (varied platforms). Service configuration: org. cost model.	Documented domains: staffing and supervision, training and competency, workflow and scheduling, utilization and monitoring, and cost and resource use. Principal bottleneck: staffing intensity and cost/funding constraints. Enabling conditions: structured training, competency checks, and clinical champions.
Vaughan-Graham et al., 2020 [59]	Setting: Community-based stroke rehabilitation (single-use laboratory session). Design: Qualitative interview study. Implementation stage: Planning; evidence unit: Device pathway.	Technology: Overground wearable powered exoskeleton for gait. Service configuration: device pathway.	Documented domains: staffing and supervision, training and competency, workflow and scheduling, safety governance, and utilization and monitoring. Principal bottleneck: usability and reliability and staff confidence. Enabling conditions: structured training, written procedures, and designated clinical support.
Hotz et al., 2024 [60]	Setting: Inpatient rehabilitation centre (neurological rehabilitation). Design: Mixed-methods feasibility study. Implementation stage: Embedded pilot; evidence unit: Device pathway.	Technology: End-effector robot-assisted gait training. Service configuration: device pathway.	Documented domains: staffing and supervision, training and competency, workflow and scheduling, eligibility and patient selection, safety governance, and utilization and monitoring. Principal bottleneck: staffing intensity and setup/workflow burden. Enabling conditions: workflow redesign, protected device time, and written operating procedures.
Pinto et al., 2020 [61]	Setting: Four SCI Model Systems rehabilitation hospitals. Design: Budget-impact analysis. Implementation stage: Planning; evidence unit: Org. cost model.	Technology: Overground robotic exoskeletons for locomotor training. Service configuration: org. cost model.	Documented domains: staffing and supervision, training and competency, workflow and scheduling, utilization and monitoring, and cost and resource use. Principal bottleneck: training needs, staff confidence, and supervision intensity. Enabling conditions: structured training, written procedures, and designated clinical support.
Thomassen et al., 2019 [62]	Setting: Inpatient spinal cord injury rehabilitation hospital implementing Ekso. Design: Qualitative interview study. Implementation stage: Early rollout; evidence unit: Device pathway.	Technology: Overground powered exoskeleton for gait training. Service configuration: device pathway.	Documented domains: staffing and supervision, training and competency, workflow and scheduling, eligibility and patient selection, safety governance, and utilization and monitoring. Principal bottleneck: staffing and staff confidence. Enabling conditions: structured device-specific training and refresher support.
Bustamante Valles et al., 2016 [63]	Setting: CREE, Chihuahua (robot/technology-assisted therapy gym). Design: Pilot randomized controlled trial. Implementation stage: Embedded pilot; evidence unit: Dedicated robotic area.	Technology: Robot/technology-assisted rehabilitation suite. Service configuration: dedicated robotic area.	Documented domains: staffing and supervision, training and competency, workflow and scheduling, utilization and monitoring, and cost and resource use. Principal bottleneck: cost/funding constraints and staff confidence. Enabling conditions: structured training, written procedures, and designated clinical support.
Masiero et al., 2014 [64]	Setting: Acute and subacute stroke inpatient rehabilitation. Design: Trial-based treatment-cost evaluation. Implementation stage: Routine use; evidence unit: Device pathway.	Technology: Upper-limb rehabilitation robot. Service configuration: device pathway.	Documented domains: staffing and supervision, training and competency, workflow and scheduling, utilization and monitoring, and cost and resource use. Principal bottleneck: cost/funding constraints and supervision intensity. Enabling conditions: structured training, written procedures, and designated clinical support.
Aguirre-Ollinger et al., 2024 [65]	Setting: Ambulatory rehabilitation clinic with a clinic-to-home extension. Design: Single-group clinic-to-home feasibility study. Implementation stage: Embedded pilot; evidence unit: Clinic-to-home.	Technology: Upper-limb end-effector robot with a web-enabled home-training interface. Service configuration: clinic-to-home.	Documented domains: staffing and supervision, training and competency, workflow and scheduling, eligibility and patient selection, safety governance, utilization and monitoring, cost and resource use, and home or hybrid delivery. Principal bottleneck: community/home logistics and space/device availability. Enabling conditions: structured training, written procedures, and designated clinical support.
Maggio et al., 2024 [66]	Setting: IRCCS neurorehabilitation service. Design: Retrospective pathway case series. Implementation stage: Routine use; evidence unit: Cross-setting pathway.	Technology: Technology-enabled neurorehabilitation pathway. Service configuration: cross-setting pathway.	Documented domains: staffing and supervision, training and competency, workflow and scheduling, eligibility and patient selection, safety governance, utilization and monitoring, and home or hybrid delivery. Principal bottleneck: staffing and staff confidence. Enabling conditions: structured training, written procedures, and designated clinical support.
Olimb Hillkirk et al., 2025 [67]	Setting: Specialized neurological rehabilitation hospital. Design: Qualitative exploratory study. Implementation stage: Routine use; evidence unit: Device pathway.	Technology: Overground lower-limb rehabilitation robot/exoskeleton. Service configuration: device pathway.	Documented domains: staffing and supervision, training and competency, workflow and scheduling, and eligibility and patient selection. Principal bottleneck: staffing and usability. Enabling conditions: structured training, competency checks, and clinical champions.
Klaic et al., 2024 [68]	Setting: Two rehabilitation services. Design: Cross-sectional clinician survey. Implementation stage: Embedded pilot; evidence unit: Device pathway.	Technology: Upper-limb end-effector rehabilitation robot. Service configuration: device pathway.	Documented domains: staffing and supervision, training and competency, and workflow and scheduling. Principal bottleneck: training needs, staff confidence, and technical support. Enabling conditions: structured training, written procedures, and designated clinical support.
Li et al., 2021 [69]	Setting: Survey of stroke rehabilitation professionals across clinical and home-based settings. Design: Mixed-methods survey. Implementation stage: Planning; evidence unit: Planning snapshot.	Technology: Rehabilitation robots used in clinical practice. Service configuration: planning snapshot.	Documented domains: staffing and supervision, training and competency, workflow and scheduling, safety governance, and cost and resource use. Principal bottleneck: cost/funding constraints and staff confidence. Enabling conditions: structured training, written procedures, and designated clinical support.
Luciani et al., 2023 [70]	Setting: Rehabilitation professionals across hospital settings (survey). Design: Cross-sectional therapist survey. Implementation stage: Planning; evidence unit: Planning snapshot.	Technology: Upper-limb rehabilitation exoskeletons. Service configuration: planning snapshot.	Documented domains: staffing and supervision, training and competency, workflow and scheduling, eligibility and patient selection, safety governance, and utilization and monitoring. Principal bottleneck: technical reliability/usability and supervision intensity. Enabling conditions: structured training, written procedures, and designated clinical support.
Nicora et al., 2025 [71]	Setting: Nationwide survey of rehabilitation healthcare practitioners. Design: National cross-sectional practitioner survey. Implementation stage: Planning; evidence unit: Planning snapshot.	Technology: Robots and Allied Digital Technologies (RADTs). Service configuration: planning snapshot.	Documented domains: training and competency, workflow and scheduling, utilization and monitoring, and cost and resource use. Principal bottleneck: training needs, staff confidence, and space/device availability. Enabling conditions: structured training, written procedures, and designated clinical support.
Flynn et al., 2019 [72]	Setting: Public health rehabilitation facility. Design: Qualitative interview study. Implementation stage: Planning; evidence unit: Device pathway.	Technology: Upper-limb rehabilitation robotics. Service configuration: device pathway.	Documented domains: staffing and supervision, training and competency, workflow and scheduling, and utilization and monitoring. Principal bottleneck: training needs, staff confidence, and technical support. Enabling conditions: structured training, written procedures, and designated clinical support.
Flynn et al., 2022 [73]	Setting: Inpatient rehabilitation unit (subacute stroke). Design: Pre-post observational implementation study. Implementation stage: Planning; evidence unit: Device pathway.	Technology: Upper-limb end-effector robot-assisted therapy. Service configuration: device pathway.	Documented domains: staffing and supervision, training and competency, workflow and scheduling, and utilization and monitoring. Principal bottleneck: setup/workflow burden and staff confidence. Enabling conditions: structured training, written procedures, and designated clinical support.
Høyer et al., 2022 [74]	Setting: Specialized rehabilitation hospital stroke unit. Design: Exploratory single-group feasibility study. Implementation stage: Early rollout; evidence unit: Device pathway.	Technology: Overground powered exoskeleton for gait rehabilitation. Service configuration: device pathway.	Documented domains: staffing and supervision, training and competency, workflow and scheduling, eligibility and patient selection, safety governance, and utilization and monitoring. Principal bottleneck: eligibility/tolerability and staff confidence. Enabling conditions: structured training, written procedures, and designated clinical support.
Pavan et al., 2024 [75]	Setting: Inpatient rehabilitation facility (subacute stroke). Design: Retrospective clinical case series. Implementation stage: Routine use; evidence unit: Multidevice pathway.	Technology: Upper-limb robotics and technology-assisted devices. Service configuration: multidevice pathway.	Documented domains: staffing and supervision, training and competency, workflow and scheduling, eligibility and patient selection, and utilization and monitoring. Principal bottleneck: guidance gap and staff confidence. Enabling conditions: workflow redesign, protected device time, and written operating procedures.

Study	Setting, design, and implementation stage	Robotic technology and service configuration	Implementation evidence extracted
Swank et al., 2020 [76]	Setting: Inpatient rehabilitation hospital. Design: Prospective feasibility case series. Implementation stage: Early rollout; evidence unit: Device pathway.	Technology: Overground robotic exoskeleton for gait training. Service configuration: device pathway.	Documented domains: staffing and supervision, training and competency, workflow and scheduling, eligibility and patient selection, safety governance, and utilization and monitoring. Principal bottleneck: staffing intensity and setup/workflow burden. Enabling conditions: structured training, competency checks, and clinical champions.
Swank et al., 2020 [77]	Setting: Single inpatient rehabilitation hospital. Design: Single-centre retrospective case series. Implementation stage: Routine use; evidence unit: Device pathway.	Technology: Overground robotic exoskeleton for gait training. Service configuration: device pathway.	Documented domains: staffing and supervision, training and competency, workflow and scheduling, eligibility and patient selection, safety governance, and utilization and monitoring. Principal bottleneck: training needs, staff confidence, and supervision intensity. Enabling conditions: structured training, written procedures, and designated clinical support.
Schuster-Amft et al., 2022 [78]	Setting: Post-acute stroke rehabilitation clinics (multicentre). Design: Multicentre single-group feasibility study. Implementation stage: Routine use; evidence unit: Device pathway.	Technology: Multi-technology, technology-assisted rehabilitation. Service configuration: device pathway.	Documented domains: staffing and supervision, training and competency, workflow and scheduling, safety governance, utilization and monitoring, and network or system configuration. Principal bottleneck: resource intensity and minor adverse events. Enabling conditions: structured training, written procedures, and designated clinical support.
Devittori et al., 2024 [79]	Setting: Clinical neurorehabilitation setting for subacute stroke patients. Design: Single-group interventional feasibility study. Implementation stage: Early rollout; evidence unit: Device pathway.	Technology: Actuated upper limb rehabilitation robot (ReHapticKnob). Service configuration: device pathway.	Documented domains: staffing and supervision, training and competency, workflow and scheduling, safety governance, and utilization and monitoring. Principal bottleneck: training before unsupervised use. Enabling conditions: competency-based training followed by staged clinical autonomy.
Johnson et al., 2017 [80]	Setting: Low-resource technology-assisted stroke rehabilitation programmes across multiple service settings. Design: Multi-site service-development report. Implementation stage: Scale-out; evidence unit: Low-resource model.	Technology: Low-cost robot/mechatronic devices and game interfaces. Service configuration: low-resource model.	Documented domains: staffing and supervision, training and competency, workflow and scheduling, utilization and monitoring, and cost and resource use. Principal bottleneck: training needs, staff confidence, and space/device availability. Enabling conditions: structured training, written procedures, and designated clinical support.
Arnold et al., 2024 [81]	Setting: Inpatient and outpatient rehabilitation (SCI continuum of care). Design: Retrospective case series. Implementation stage: Routine use; evidence unit: Continuum pathway.	Technology: Overground exoskeleton (Ekso). Service configuration: continuum pathway.	Documented domains: staffing and supervision, workflow and scheduling, eligibility and patient selection, safety governance, utilization and monitoring, and network or system configuration. Principal bottleneck: cross-setting coordination. Enabling conditions: routine utilization metrics, feedback, and service-level monitoring.
van Dellen et al., 2025 [82]	Setting: Routine robot-assisted gait therapy (Lokomat). Design: Mixed-methods development and feasibility study. Implementation stage: Early rollout; evidence unit: Measurement tool.	Technology: Treadmill-based robot-assisted gait therapy (Lokomat). Service configuration: measurement tool.	Documented domains: workflow and scheduling, eligibility and patient selection, and measurement tools. Principal bottleneck: lack of standardised guidance. Enabling conditions: structured measurement, repeated feedback, and documented decision criteria.
Fundarò et al., 2023 [83]	Setting: Neurological rehabilitation. Design: Multicentre observational pre-post study. Implementation stage: Early rollout; evidence unit: Measurement tool.	Technology: TAR and exergaming (device type varied; NR). Service configuration: measurement tool.	Documented domains: workflow and scheduling, utilization and monitoring, and measurement tools. Principal bottleneck: routine perception monitoring. Enabling conditions: structured measurement, repeated feedback, and documented decision criteria.
Mensah-Gourmel et al., 2024 [84]	Setting: Online survey of pediatric motor rehabilitation professionals. Design: Cross-sectional professional survey. Implementation stage: Routine snapshot; evidence unit: Planning snapshot.	Technology: DT4R: ROBTS, VR/active video gaming (VR-aVG), telehealth. Service configuration: planning snapshot.	Documented domains: training and competency, workflow and scheduling, utilization and monitoring, and cost and resource use. Principal bottleneck: staff confidence and cost/funding constraints. Enabling conditions: structured training, competency checks, and clinical champions.
Kang et al., 2016 [85]	Setting: Multi-site hospital survey (38 hospitals). Design: Multi-site cross-sectional professional survey. Implementation stage: Planning; evidence unit: Planning snapshot.	Technology: Gait-training robots (treadmill-type and overground). Service configuration: planning snapshot.	Documented domains: workflow and scheduling, eligibility and patient selection, and cost and resource use. Principal bottleneck: cost/funding constraints and candidate selection. Enabling conditions: locally defined procedures, assigned responsibilities, and routine feedback.
Patarini et al., 2024 [86]	Setting: Spinal Cord Unit rehabilitation ward. Design: Within-participant non-randomized experimental study. Implementation stage: Routine use; evidence unit: Device pathway.	Technology: Treadmill-based robotic-assisted gait training. Service configuration: device pathway.	Documented domains: staffing and supervision, workflow and scheduling, and utilization and monitoring. Principal bottleneck: staffing intensity. Enabling conditions: patient-centred goal setting, motivation, and therapist support.
Reichl et al., 2020 [87]	Setting: Inpatient neurological rehabilitation (gait center training). Design: Retrospective cohort study. Implementation stage: Early rollout; evidence unit: Dedicated robotic area.	Technology: Gait center training programme. Service configuration: dedicated robotic area.	Documented domains: workflow and scheduling, and utilization and monitoring. Principal bottleneck: capacity and workflow demands. Enabling conditions: centralized delivery, coordinated scheduling, and standardized procedures.
Huang et al., 2018 [88]	Setting: Clinical translation of EMG-driven robot-hand training in routine rehabilitation. Design: Non-randomized comparative intervention study. Implementation stage: Routine use; evidence unit: Device pathway.	Technology: EMG-driven robot hand training system. Service configuration: device pathway.	Documented domains: staffing and supervision, training and competency, workflow and scheduling, and utilization and monitoring. Principal bottleneck: setup/workflow burden and staff confidence. Enabling conditions: structured training, written procedures, and designated clinical support.
Calabrò et al., 2020 [89]	Setting: Regional neurorehabilitation network (hub-and-spoke). Design: Regional service-model report. Implementation stage: Early rollout; evidence unit: Regional network.	Technology: Multi-device programme comprising gait robots, exoskeletons, and upper-limb systems. Service configuration: regional network.	Documented domains: staffing and supervision, training and competency, workflow and scheduling, safety governance, utilization and monitoring, cost and resource use, and network or system configuration. Principal bottleneck: space/device infrastructure and cost/funding constraints. Enabling conditions: structured training, written procedures, and designated clinical support.
De Cola et al., 2023 [90]	Setting: Regional neurorehabilitation hub-and-spoke network. Design: Retrospective controlled before-after service evaluation. Implementation stage: Scale-out; evidence unit: Regional network.	Technology: Multi-device advanced neurorehabilitation programme deployed through a hub-and-spoke network. Service configuration: regional network.	Documented domains: staffing and supervision, training and competency, workflow and scheduling, safety governance, utilization and monitoring, and network or system configuration. Principal bottleneck: space/device infrastructure and lack of standardized referral and delivery procedures. Enabling conditions: structured training, written procedures, and designated clinical support.
Ielo et al., 2024 [91]	Setting: Regional neurorehabilitation hub-and-spoke network. Design: Retrospective controlled before-after service evaluation. Implementation stage: Scale-out; evidence unit: Regional network.	Technology: Advanced neurorehabilitation technologies distributed across hub and spoke centres. Service configuration: regional network.	Documented domains: staffing and supervision, training and competency, workflow and scheduling, safety governance, utilization and monitoring, cost and resource use, and network or system configuration. Principal bottleneck: cost and funding constraints. Enabling conditions: shared network governance, referral pathways, and technical support.
Manuli et al., 2020 [92]	Setting: Inpatient neurorehabilitation. Design: Exploratory single-group pre-post study. Implementation stage: Routine use; evidence unit: Multidevice pathway.	Technology: Multi-device rehabilitation robotics combined with virtual reality. Service configuration: multidevice pathway.	Documented domains: staffing and supervision, training and competency, workflow and scheduling, safety governance, and utilization and monitoring. Principal bottleneck: space/device infrastructure and patient eligibility/fit. Enabling conditions: structured training, written procedures, and designated clinical support.
Fundarò et al., 2026 [93]	Setting: Two neurorehabilitation centres providing robot-assisted gait training. Design: Multicentre cross-sectional therapist survey. Implementation stage: Routine use; evidence unit: Device pathway.	Technology: Treadmill-based gait robot (tethered exoskeleton). Service configuration: device pathway.	Documented domains: staffing and supervision, training and competency, workflow and scheduling, eligibility and patient selection, and safety governance. Principal bottleneck: training needs, staff confidence, and supervision intensity. Enabling conditions: structured training, written procedures, and designated clinical support.
Hesse et al., 2014 [94]	Setting: Inpatient neurological rehabilitation. Design: Single-blind randomized controlled trial. Implementation stage: Routine use; evidence unit: Dedicated robotic area.	Technology: Upper-limb robotic and electromechanical devices organized within a multi-patient treatment area. Service configuration: dedicated robotic area.	Documented domains: staffing and supervision, workflow and scheduling, utilization and monitoring, and cost and resource use. Principal bottleneck: staffing intensity and cost/funding constraints. Enabling conditions: workflow redesign, protected device time, and written operating procedures.
Nedergård et al., 2023 [95]	Setting: Post-stroke gait rehabilitation intervention (chronic phase). Design: Qualitative interview study nested in a training programme. Implementation stage: Routine use; evidence unit: Device pathway.	Technology: Treadmill-based powered exoskeleton for gait training. Service configuration: device pathway.	Documented domains: staffing and supervision, training and competency, workflow and scheduling, eligibility and patient selection, and safety governance. Principal bottleneck: staffing and staff confidence. Enabling conditions: structured device-specific training and refresher support.
Ouendi et al., 2024 [96]	Setting: Clinical rehabilitation services using a gait robot. Design: Mixed-methods clinician study. Implementation stage: Routine use; evidence unit: Device pathway.	Technology: Treadmill-based gait robot (tethered exoskeleton). Service configuration: device pathway.	Documented domains: training and competency, workflow and scheduling, and safety governance. Principal bottleneck: usability/reliability and setup/workflow burden. Enabling conditions: structured training, written procedures, and designated clinical support.
Phelan et al., 2015 [97]	Setting: Paediatric neurorehabilitation (cerebral palsy). Design: Interpretivist qualitative study. Implementation stage: Routine use; evidence unit: Device pathway.	Technology: Treadmill-based gait robot (tethered exoskeleton). Service configuration: device pathway.	Documented domains: staffing and supervision, workflow and scheduling, and safety governance. Principal bottleneck: staffing intensity and setup/workflow burden. Enabling conditions: patient-centred goal setting, motivation, and therapist support.
Stephenson et al., 2018 [98]	Setting: Stroke rehabilitation centre (upper-limb rehabilitation). Design: Qualitative interview study. Implementation stage: Routine use; evidence unit: Device pathway.	Technology: Upper-limb end-effector robot for repetitive reaching. Service configuration: device pathway.	Documented domains: staffing and supervision, training and competency, workflow and scheduling, and safety governance. Principal bottleneck: staffing and staff confidence. Enabling conditions: structured device-specific training and refresher support.
Mazzoleni et al., 2017 [99]	Setting: Chronic stroke outpatient/inpatient neurorehabilitation (multi-centre). Design: Multicentre retrospective uncontrolled case series. Implementation stage: Routine use; evidence unit: Device pathway.	Technology: Lower-limb end-effector gait training robot. Service configuration: device pathway.	Documented domains: training and competency, workflow and scheduling, eligibility and patient selection, safety governance, and network or system configuration. Principal bottleneck: setup and workflow burden. Enabling conditions: structured device-specific training and refresher support.
Maggio et al., 2024 [100]	Setting: Inpatient advanced rehabilitation pathway. Design: Retrospective cohort study. Implementation stage: Sustainment; evidence unit: Multidevice pathway.	Technology: Multidisciplinary pathway combining robot-assisted therapy and conventional physiotherapy. Service configuration: multidevice pathway.	Documented domains: staffing and supervision, training and competency, workflow and scheduling, and utilization and monitoring. Principal bottleneck: staffing and infrastructure. Enabling conditions: structured training, written procedures, and designated clinical support.

Note. Each row represents one included report. Setting, design, technology, service configuration, and implementation evidence are presented at study level. Constructs were recorded as explicit when named by study authors and otherwise mapped from source-linked operational descriptions using prespecified definitions.

Abbreviations: CFIR, Consolidated Framework for Implementation Research; ERIC, Expert Recommendations for Implementing Change; IRCCS, Scientific Institute for Research, Hospitalization and Healthcare; IRF, inpatient rehabilitation facility; QI, quality improvement; RR, rehabilitation robotics; SCI, spinal cord injury; TBI, traumatic brain injury.

Supplementary Table S4. Study-level translational readiness signals

Study	Implementation stage and unit	Documented readiness signals	Principal bottleneck and enabling conditions	Translational implication
Cao et al. 2021 [46]	Early rollout; Device pathway.	Staffing and supervision, training and competency, workflow and scheduling, safety governance, utilization and monitoring, and cost and resource use.	Bottleneck: setup/workflow burden and staff confidence. Enabling conditions: structured training, written procedures, and designated clinical support.	Feasibility is supported; longer follow-up is needed for penetration, durability, maintenance, and resource use.
Flynn et al. 2025 [47]	Routine use; Device pathway.	Staffing and supervision, training and competency, workflow and scheduling, and utilization and monitoring.	Bottleneck: setup/workflow burden and staff confidence. Enabling conditions: structured training, written procedures, and designated clinical support.	Operational integration is documented in the reported setting; transferability and sustainment depend on explicit local resource reporting.
Gillespie et al. 2023 [48]	Routine use; Device pathway.	Staffing and supervision, training and competency, workflow and scheduling, eligibility and patient selection, safety governance, and utilization and monitoring.	Bottleneck: staffing and staff confidence. Enabling conditions: standardized eligibility screening, written protocols, and progression criteria.	Operational integration is documented in the reported setting; transferability and sustainment depend on explicit local resource reporting.
Heinemann et al. 2018 [49]	Early rollout; Cross-centre programme.	Staffing and supervision, training and competency, workflow and scheduling, eligibility and patient selection, safety governance, utilization and monitoring, cost and resource use, and network or system configuration.	Bottleneck: cost/funding constraints and staff confidence. Enabling conditions: structured training, written procedures, and designated clinical support.	Feasibility is supported; longer follow-up is needed for penetration, durability, maintenance, and resource use.
Pila et al. 2022 [50]	Routine use; Device pathway.	Staffing and supervision, training and competency, workflow and scheduling, utilization and monitoring, and cost and resource use.	Bottleneck: staff confidence and setup/workflow burden. Enabling conditions: routine utilization metrics, feedback, and service-level monitoring.	Operational integration is documented in the reported setting; transferability and sustainment depend on explicit local resource reporting.
Read et al. 2020 [51]	Early rollout; Continuum pathway.	Staffing and supervision, training and competency, workflow and scheduling, eligibility and patient selection, safety governance, and network or system configuration.	Bottleneck: eligibility/tolerability and staff confidence. Enabling conditions: structured training, written procedures, and designated clinical support.	Feasibility is supported; longer follow-up is needed for penetration, durability, maintenance, and resource use.
Warmbein et al. 2023 [52]	Mixed phase; Design insights.	Staffing and supervision, training and competency, workflow and scheduling, safety governance, and cost and resource use.	Bottleneck: staff confidence and cost/funding constraints. Enabling conditions: workflow redesign, protected device time, and written operating procedures.	The report informs service design; prospective evaluation is required before broader implementation.
Buschfort et al. 2010 [53]	Early rollout; Dedicated robotic area.	Staffing and supervision, training and competency, workflow and scheduling, eligibility and patient selection, safety governance, and utilization and monitoring.	Bottleneck: staffing intensity and cost/funding constraints. Enabling conditions: group or semi-supervised delivery with explicit staff responsibilities.	Feasibility is supported; longer follow-up is needed for penetration, durability, maintenance, and resource use.
Aprile et al. 2019 [54]	Early rollout; Multidevice pathway.	Staffing and supervision, training and competency, workflow and scheduling, eligibility and patient selection, safety governance, and network or system configuration.	Bottleneck: staffing and infrastructure. Enabling conditions: workflow redesign, protected device time, and written operating procedures.	Feasibility is supported; longer follow-up is needed for penetration, durability, maintenance, and resource use.
Aprile et al. 2019 [55]	Routine use; Dedicated robotic area.	Staffing and supervision, training and competency, workflow and scheduling, and utilization and monitoring.	Bottleneck: staffing and staff confidence. Enabling conditions: workflow redesign, protected device time, and written operating procedures.	Operational integration is documented in the reported setting; transferability and sustainment depend on explicit local resource reporting.
Gagnon et al. 2018 [56]	Routine use; Device pathway.	Staffing and supervision, training and competency, workflow and scheduling, eligibility and patient selection, safety governance, and utilization and monitoring.	Bottleneck: eligibility and community logistics. Enabling conditions: structured training, written procedures, and designated clinical support.	Operational integration is documented in the reported setting; transferability and sustainment depend on explicit local resource reporting.
Gower et al. 2024 [57]	Routine use; Org. cost model.	Staffing and supervision, workflow and scheduling, utilization and monitoring, and cost and resource use.	Bottleneck: staffing intensity and setup/workflow burden. Enabling conditions: group or semi-supervised delivery with explicit staff responsibilities.	Operational integration is documented in the reported setting; transferability and sustainment depend on explicit local resource reporting.
Gower et al. 2025 [58]	Scale-out; Org. cost model.	Staffing and supervision, training and competency, workflow and scheduling, utilization and monitoring, and cost and resource use.	Bottleneck: staffing intensity and cost/funding constraints. Enabling conditions: structured training, competency checks, and clinical champions.	Network expansion is documented; consistency, equity, penetration, and maintenance should be monitored across sites.
Vaughan-Graham et al. 2020 [59]	Planning; Device pathway.	Staffing and supervision, training and competency, workflow and scheduling, safety governance, and utilization and monitoring.	Bottleneck: usability and reliability and staff confidence. Enabling conditions: structured training, written procedures, and designated clinical support.	Prospective feasibility testing, resource specification, and predefined implementation outcomes are required before routine adoption.
Hotz et al. 2024 [60]	Embedded pilot; Device pathway.	Staffing and supervision, training and competency, workflow and scheduling, eligibility and patient selection, safety governance, and utilization and monitoring.	Bottleneck: staffing intensity and setup/workflow burden. Enabling conditions: workflow redesign, protected device time, and written operating procedures.	Feasibility is supported; longer follow-up is needed for penetration, durability, maintenance, and resource use.
Pinto et al. 2020 [61]	Planning; Org. cost model.	Staffing and supervision, training and competency, workflow and scheduling, utilization and monitoring, and cost and resource use.	Bottleneck: training needs, staff confidence, and supervision intensity. Enabling conditions: structured training, written procedures, and designated clinical support.	Prospective feasibility testing, resource specification, and predefined implementation outcomes are required before routine adoption.
Thomassen et al. 2019 [62]	Early rollout; Device pathway.	Staffing and supervision, training and competency, workflow and scheduling, eligibility and patient selection, safety governance, and utilization and monitoring.	Bottleneck: staffing and staff confidence. Enabling conditions: structured device-specific training and refresher support.	Feasibility is supported; longer follow-up is needed for penetration, durability, maintenance, and resource use.
Bustamante Valles et al. 2016 [63]	Embedded pilot; Dedicated robotic area.	Staffing and supervision, training and competency, workflow and scheduling, utilization and monitoring, and cost and resource use.	Bottleneck: cost/funding constraints and staff confidence. Enabling conditions: structured training, written procedures, and designated clinical support.	Feasibility is supported; longer follow-up is needed for penetration, durability, maintenance, and resource use.
Masiero et al. 2014 [64]	Routine use; Device pathway.	Staffing and supervision, training and competency, workflow and scheduling, utilization and monitoring, and cost and resource use.	Bottleneck: cost/funding constraints and supervision intensity. Enabling conditions: structured training, written procedures, and designated clinical support.	Operational integration is documented in the reported setting; transferability and sustainment depend on explicit local resource reporting.
Aguirre-Ollinger et al. 2024 [65]	Embedded pilot; Clinic-to-home.	Staffing and supervision, training and competency, workflow and scheduling, eligibility and patient selection, safety governance, utilization and monitoring, cost and resource use, and home or hybrid delivery.	Bottleneck: community/home logistics and space/device availability. Enabling conditions: structured training, written procedures, and designated clinical support.	Feasibility is supported; longer follow-up is needed for penetration, durability, maintenance, and resource use.
Maggio et al. 2024 [66]	Routine use; Cross-setting pathway.	Staffing and supervision, training and competency, workflow and scheduling, eligibility and patient selection, safety governance, utilization and monitoring, and home or hybrid delivery.	Bottleneck: staffing and staff confidence. Enabling conditions: structured training, written procedures, and designated clinical support.	Operational integration is documented in the reported setting; transferability and sustainment depend on explicit local resource reporting.
Olimb Hillkirk et al. 2025 [67]	Routine use; Device pathway.	Staffing and supervision, training and competency, workflow and scheduling, and eligibility and patient selection.	Bottleneck: staffing and usability. Enabling conditions: structured training, competency checks, and clinical champions.	Operational integration is documented in the reported setting; transferability and sustainment depend on explicit local resource reporting.
Klaic et al. 2024 [68]	Embedded pilot; Device pathway.	Staffing and supervision, training and competency, and workflow and scheduling.	Bottleneck: training needs, staff confidence, and technical support. Enabling conditions: structured training, written procedures, and designated clinical support.	Feasibility is supported; longer follow-up is needed for penetration, durability, maintenance, and resource use.
Li et al. 2021 [69]	Planning; Planning snapshot.	Staffing and supervision, training and competency, workflow and scheduling, safety governance, and cost and resource use.	Bottleneck: cost/funding constraints and staff confidence. Enabling conditions: structured training, written procedures, and designated clinical support.	Prospective feasibility testing, resource specification, and predefined implementation outcomes are required before routine adoption.
Luciani et al. 2023 [70]	Planning; Planning snapshot.	Staffing and supervision, training and competency, workflow and scheduling, eligibility and patient selection, safety governance, and utilization and monitoring.	Bottleneck: technical reliability/usability and supervision intensity. Enabling conditions: structured training, written procedures, and designated clinical support.	Prospective feasibility testing, resource specification, and predefined implementation outcomes are required before routine adoption.
Nicora et al. 2025 [71]	Planning; Planning snapshot.	Training and competency, workflow and scheduling, utilization and monitoring, and cost and resource use.	Bottleneck: training needs, staff confidence, and space/device availability. Enabling conditions: structured training, written procedures, and designated clinical support.	Prospective feasibility testing, resource specification, and predefined implementation outcomes are required before routine adoption.
Flynn et al. 2019 [72]	Planning; Device pathway.	Staffing and supervision, training and competency, workflow and scheduling, and utilization and monitoring.	Bottleneck: training needs, staff confidence, and technical support. Enabling conditions: structured training, written procedures, and designated clinical support.	Prospective feasibility testing, resource specification, and predefined implementation outcomes are required before routine adoption.
Flynn et al. 2022 [73]	Planning; Device pathway.	Staffing and supervision, training and competency, workflow and scheduling, and utilization and monitoring.	Bottleneck: setup/workflow burden and staff confidence. Enabling conditions: structured training, written procedures, and designated clinical support.	Prospective feasibility testing, resource specification, and predefined implementation outcomes are required before routine adoption.
Høyer et al. 2022 [74]	Early rollout; Device pathway.	Staffing and supervision, training and competency, workflow and scheduling, eligibility and patient selection, safety governance, and utilization and monitoring.	Bottleneck: eligibility/tolerability and staff confidence. Enabling conditions: structured training, written procedures, and designated clinical support.	Feasibility is supported; longer follow-up is needed for penetration, durability, maintenance, and resource use.
Pavan et al. 2024 [75]	Routine use; Multidevice pathway.	Staffing and supervision, training and competency, workflow and scheduling, eligibility and patient selection, and utilization and monitoring.	Bottleneck: guidance gap and staff confidence. Enabling conditions: workflow redesign, protected device time, and written operating procedures.	Operational integration is documented in the reported setting; transferability and sustainment depend on explicit local resource reporting.
Swank et al. 2020 [76]	Early rollout; Device pathway.	Staffing and supervision, training and competency, workflow and scheduling, eligibility and patient selection, safety governance, and utilization and monitoring.	Bottleneck: staffing intensity and setup/workflow burden. Enabling conditions: structured training, competency checks, and clinical champions.	Feasibility is supported; longer follow-up is needed for penetration, durability, maintenance, and resource use.
Swank et al. 2020 [77]	Routine use; Device pathway.	Staffing and supervision, training and competency, workflow and scheduling, eligibility and patient selection, safety governance, and utilization and monitoring.	Bottleneck: training needs, staff confidence, and supervision intensity. Enabling conditions: structured training, written procedures, and designated clinical support.	Operational integration is documented in the reported setting; transferability and sustainment depend on explicit local resource reporting.
Schuster-Amft et al. 2022 [78]	Routine use; Device pathway.	Staffing and supervision, training and competency, workflow and scheduling, safety governance, utilization and monitoring, and network or system configuration.	Bottleneck: resource intensity and minor adverse events. Enabling conditions: structured training, written procedures, and designated clinical support.	Operational integration is documented in the reported setting; transferability and sustainment depend on explicit local resource reporting.
Devittori et al. 2024 [79]	Early rollout; Device pathway.	Staffing and supervision, training and competency, workflow and scheduling, safety governance, and utilization and monitoring.	Bottleneck: training before unsupervised use. Enabling conditions: competency-based training followed by staged clinical autonomy.	Feasibility is supported; longer follow-up is needed for penetration, durability, maintenance, and resource use.
Johnson et al. 2017 [80]	Scale-out; Low-resource model.	Staffing and supervision, training and competency, workflow and scheduling, utilization and monitoring, and cost and resource use.	Bottleneck: training needs, staff confidence, and space/device availability. Enabling conditions: structured training, written procedures, and designated clinical support.	Network expansion is documented; consistency, equity, penetration, and maintenance should be monitored across sites.
Arnold et al. 2024 [81]	Routine use; Continuum pathway.	Staffing and supervision, workflow and scheduling, eligibility and patient selection, safety governance, utilization and monitoring, and network or system configuration.	Bottleneck: cross-setting coordination. Enabling conditions: routine utilization metrics, feedback, and service-level monitoring.	Operational integration is documented in the reported setting; transferability and sustainment depend on explicit local resource reporting.
van Dellen et al. 2025 [82]	Early rollout; Measurement tool.	Workflow and scheduling, eligibility and patient selection, and measurement tools.	Bottleneck: lack of standardised guidance. Enabling conditions: structured measurement, repeated feedback, and documented decision criteria.	Feasibility is supported; longer follow-up is needed for penetration, durability, maintenance, and resource use.
Fundarò et al. 2023 [83]	Early rollout; Measurement tool.	Workflow and scheduling, utilization and monitoring, and measurement tools.	Bottleneck: routine perception monitoring. Enabling conditions: structured measurement, repeated feedback, and documented decision criteria.	Feasibility is supported; longer follow-up is needed for penetration, durability, maintenance, and resource use.
Mensah-Gourmel et al. 2024 [84]	Routine snapshot; Planning snapshot.	Training and competency, workflow and scheduling, utilization and monitoring, and cost and resource use.	Bottleneck: staff confidence and cost/funding constraints. Enabling conditions: structured training, competency checks, and clinical champions.	The report informs service design; prospective evaluation is required before broader implementation.

Study	Implementation stage and unit	Documented readiness signals	Principal bottleneck and enabling conditions	Translational implication
Kang et al. 2016 [85]	Planning; Planning snapshot.	Workflow and scheduling, eligibility and patient selection, and cost and resource use.	Bottleneck: cost/funding constraints and candidate selection. Enabling conditions: locally defined procedures, assigned responsibilities, and routine feedback.	Prospective feasibility testing, resource specification, and predefined implementation outcomes are required before routine adoption.
Patarini et al. 2024 [86]	Routine use; Device pathway.	Staffing and supervision, workflow and scheduling, and utilization and monitoring.	Bottleneck: staffing intensity. Enabling conditions: patient-centred goal setting, motivation, and therapist support.	Operational integration is documented in the reported setting; transferability and sustainment depend on explicit local resource reporting.
Reichl et al. 2020 [87]	Early rollout; Dedicated robotic area.	Workflow and scheduling, and utilization and monitoring.	Bottleneck: capacity and workflow demands. Enabling conditions: centralized delivery, coordinated scheduling, and standardized procedures.	Feasibility is supported; longer follow-up is needed for penetration, durability, maintenance, and resource use.
Huang et al. 2018 [88]	Routine use; Device pathway.	Staffing and supervision, training and competency, workflow and scheduling, and utilization and monitoring.	Bottleneck: setup/workflow burden and staff confidence. Enabling conditions: structured training, written procedures, and designated clinical support.	Operational integration is documented in the reported setting; transferability and sustainment depend on explicit local resource reporting.
Calabrò et al. 2020 [89]	Early rollout; Regional network.	Staffing and supervision, training and competency, workflow and scheduling, safety governance, utilization and monitoring, cost and resource use, and network or system configuration.	Bottleneck: space/device infrastructure and cost/funding constraints. Enabling conditions: structured training, written procedures, and designated clinical support.	Feasibility is supported; longer follow-up is needed for penetration, durability, maintenance, and resource use.
De Cola et al. 2023 [90]	Scale-out; Regional network.	Staffing and supervision, training and competency, workflow and scheduling, safety governance, utilization and monitoring, and network or system configuration.	Bottleneck: space/device infrastructure and lack of standardized referral and delivery procedures. Enabling conditions: structured training, written procedures, and designated clinical support.	Network expansion is documented; consistency, equity, penetration, and maintenance should be monitored across sites.
Ielo et al. 2024 [91]	Scale-out; Regional network.	Staffing and supervision, training and competency, workflow and scheduling, safety governance, utilization and monitoring, cost and resource use, and network or system configuration.	Bottleneck: cost and funding constraints. Enabling conditions: shared network governance, referral pathways, and technical support.	Network expansion is documented; consistency, equity, penetration, and maintenance should be monitored across sites.
Manuli et al. 2020 [92]	Routine use; Multidevice pathway.	Staffing and supervision, training and competency, workflow and scheduling, safety governance, and utilization and monitoring.	Bottleneck: space/device infrastructure and patient eligibility/fit. Enabling conditions: structured training, written procedures, and designated clinical support.	Operational integration is documented in the reported setting; transferability and sustainment depend on explicit local resource reporting.
Fundarò et al. 2026 [93]	Routine use; Device pathway.	Staffing and supervision, training and competency, workflow and scheduling, eligibility and patient selection, and safety governance.	Bottleneck: training needs, staff confidence, and supervision intensity. Enabling conditions: structured training, written procedures, and designated clinical support.	Operational integration is documented in the reported setting; transferability and sustainment depend on explicit local resource reporting.
Hesse et al. 2014 [94]	Routine use; Dedicated robotic area.	Staffing and supervision, workflow and scheduling, utilization and monitoring, and cost and resource use.	Bottleneck: staffing intensity and cost/funding constraints. Enabling conditions: workflow redesign, protected device time, and written operating procedures.	Operational integration is documented in the reported setting; transferability and sustainment depend on explicit local resource reporting.
Nedergård et al. 2023 [95]	Routine use; Device pathway.	Staffing and supervision, training and competency, workflow and scheduling, eligibility and patient selection, and safety governance.	Bottleneck: staffing and staff confidence. Enabling conditions: structured device-specific training and refresher support.	Operational integration is documented in the reported setting; transferability and sustainment depend on explicit local resource reporting.
Ouendi et al. 2024 [96]	Routine use; Device pathway.	Training and competency, workflow and scheduling, and safety governance.	Bottleneck: usability/reliability and setup/workflow burden. Enabling conditions: structured training, written procedures, and designated clinical support.	Operational integration is documented in the reported setting; transferability and sustainment depend on explicit local resource reporting.
Phelan et al. 2015 [97]	Routine use; Device pathway.	Staffing and supervision, workflow and scheduling, and safety governance.	Bottleneck: staffing intensity and setup/workflow burden. Enabling conditions: patient-centred goal setting, motivation, and therapist support.	Operational integration is documented in the reported setting; transferability and sustainment depend on explicit local resource reporting.
Stephenson et al. 2018 [98]	Routine use; Device pathway.	Staffing and supervision, training and competency, workflow and scheduling, and safety governance.	Bottleneck: staffing and staff confidence. Enabling conditions: structured device-specific training and refresher support.	Operational integration is documented in the reported setting; transferability and sustainment depend on explicit local resource reporting.
Mazzoleni et al. 2017 [99]	Routine use; Device pathway.	Training and competency, workflow and scheduling, eligibility and patient selection, safety governance, and network or system configuration.	Bottleneck: setup and workflow burden. Enabling conditions: structured device-specific training and refresher support.	Operational integration is documented in the reported setting; transferability and sustainment depend on explicit local resource reporting.
Maggio et al. 2024 [100]	Sustainment; Multidevice pathway.	Staffing and supervision, training and competency, workflow and scheduling, and utilization and monitoring.	Bottleneck: staffing and infrastructure. Enabling conditions: structured training, written procedures, and designated clinical support.	Mature service evidence; long-term cost, staff turnover, maintenance, and outcome stability remain priority measures.

Note. Readiness signals indicate whether a service-relevant domain was reported; they do not quantify benefit or establish causal importance. Implementation stage denotes the most advanced phase documented in each report.

Abbreviations: CT, cost and resource use; EL, eligibility and patient selection; HM, home or hybrid delivery; MT, measurement tool; NW, network or system configuration; SG, safety governance; ST, staffing and supervision; TR, training and competency; UM, utilization and monitoring; WF, workflow and scheduling.

Supplementary Table S5. Explicit versus inferred framework coding and construct-level mapping

Panel	Framework / domain	Reported or mapped finding	Operational interpretation
Explicit versus inferred coding	CFIR determinants	Explicit: 7 (12.7%); Inferred/reviewer-mapped: 48 (87.3%)	Explicit determinant language was retained when authors identified barriers/facilitators; otherwise operational descriptions were mapped to CFIR constructs.
Explicit versus inferred coding	ERIC strategies	Explicit: 9 (16.4%); Inferred/reviewer-mapped: 46 (83.6%)	Named implementation actions were retained when present; otherwise training, protocolisation, workflow redesign, monitoring, or support activities were mapped to ERIC families.
Explicit versus inferred coding	Proctor outcomes	Explicit: 35 (63.6%); Inferred/reviewer-mapped: 20 (36.4%)	Outcomes explicitly named as feasibility, acceptability, usability, adoption, cost, or related terms were coded as explicit; service metrics were mapped when terminology was absent.
Explicit versus inferred coding	All frameworks	Explicit: 55 (100%) had at least one implementation-relevant element; Inferred/reviewer-mapped: 47 (85.5%) required inference for at least one framework domain	Inference was source-linked and flagged. Overlapping constructs required distinct meanings; ambiguous passages were coded to the narrower construct or left unmapped; a second reviewer verified all fields against the source text.
CFIR construct-level summary	Inner Setting	Available resources — 46 (83.6%)	Staff time, device access, space, scheduling capacity, training resources.
CFIR construct-level summary	Inner Setting	Access to knowledge and information — 29 (52.7%)	Onboarding, competency materials, device-specific training, super-users.
CFIR construct-level summary	Inner Setting	Implementation climate/workflow compatibility — 33 (60.0%)	Fit with existing therapy sessions, slot fill, priority conflicts.
CFIR construct-level summary	Intervention Characteristics	Complexity — 43 (78.2%)	Setup, donning/doffing, calibration, safety procedures, therapist workload.
CFIR construct-level summary	Intervention Characteristics	Adaptability — 33 (60.0%)	Dose, assistance level, device parameters, pathway tailoring.
CFIR construct-level summary	Intervention Characteristics	Cost — 22 (40.0%)	Purchase, maintenance, staffing, reimbursement, utilization assumptions.
CFIR construct-level summary	Characteristics of Individuals	Knowledge and beliefs/self-efficacy — 28 (50.9%)	Clinician confidence, perceived usefulness, patient motivation.
CFIR construct-level summary	Process	Planning and engaging — 33 (60.0%)	Champions, steering groups, training plans, local protocols.
CFIR construct-level summary	Process	Reflecting and evaluating — 21 (38.2%)	Audit, device logs, service metrics, feedback loops.
CFIR construct-level summary	Outer Setting	Financing and reimbursement — 14 (25.5%)	Funding, procurement, reimbursement, out-of-region costs.
CFIR construct-level summary	Outer Setting	Patient needs and resources — 21 (38.2%)	Access, travel, caregiver support, home/community context.

Note. Percentages use 55 included reports as the denominator. Explicit coding denotes named implementation terminology in the primary report; mapped coding denotes classification from operational descriptions.
Abbreviations: CFIR, Consolidated Framework for Implementation Research; ERIC, Expert Recommendations for Implementing Change; RR, rehabilitation robotics.

Supplementary Table S6. Report-level design-to-appraisal crosswalk and instrument allocation (N = 55)

Study	Table 1 design category	Study-specific design	Primary instrument	**Instrument-selection rationale**	Appraisal unit	**Role in synthesis**
Cao et al., 2021 [46]	Quality improvement/service evaluation	Quality-improvement initiative	QI-MQCS	Quality-improvement or service-model report centred on local implementation change.	Included report; no parent or companion article substituted.	Context only; no exclusion or weighting.
Flynn et al., 2025 [47]	Qualitative	Qualitative focus-group study with embedded usability survey	CASP qualitative	Qualitative inquiry was the primary design and source of implementation evidence.	Included report; no parent or companion article substituted.	Context only; no exclusion or weighting.
Gillespie et al., 2023 [48]	Case series	Retrospective descriptive case series with therapist survey	JBI case series	Descriptive clinical series without a comparator group.	Included report; no parent or companion article substituted.	Context only; no exclusion or weighting.
Heinemann et al., 2018 [49]	Qualitative	Multicentre qualitative focus-group study	CASP qualitative	Qualitative inquiry was the primary design and source of implementation evidence.	Included report; no parent or companion article substituted.	Context only; no exclusion or weighting.
Pila et al., 2022 [50]	Non-randomized comparative intervention	Retrospective non-randomized comparative intervention study	ROBINS-I	Explicit non-randomized intervention-effect comparison between groups or defined service periods.	Included report; no parent or companion article substituted.	Context only; no exclusion or weighting.
Read et al., 2020 [51]	Qualitative	Exploratory qualitative interview study	CASP qualitative	Qualitative inquiry was the primary design and source of implementation evidence.	Included report; no parent or companion article substituted.	Context only; no exclusion or weighting.
Warmbein et al., 2023 [52]	Qualitative	Exploratory qualitative interview study	CASP qualitative	Qualitative inquiry was the primary design and source of implementation evidence.	Included report; no parent or companion article substituted.	Context only; no exclusion or weighting.
Buschfort et al., 2010 [53]	Quasi-experimental	Open single-group pre-post study	JBI quasi-experimental	Non-randomized intervention evaluation without the comparative intervention-effect structure assigned to ROBINS-I.	Included report; no parent or companion article substituted.	Context only; no exclusion or weighting.
Aprile et al., 2019 [54]	Quasi-experimental	Single-group feasibility study	JBI quasi-experimental	Non-randomized intervention evaluation without the comparative intervention-effect structure assigned to ROBINS-I.	Included report; no parent or companion article substituted.	Context only; no exclusion or weighting.
Aprile et al., 2019 [55]	Quasi-experimental	Observational single-group feasibility study	JBI quasi-experimental	Non-randomized intervention evaluation without the comparative intervention-effect structure assigned to ROBINS-I.	Included report; no parent or companion article substituted.	Context only; no exclusion or weighting.
Gagnon et al., 2018 [56]	Case series	Prospective single-group feasibility case series	JBI case series	Descriptive clinical series without a comparator group.	Included report; no parent or companion article substituted.	Context only; no exclusion or weighting.
Gower et al., 2024 [57]	Economic evaluation/modelling	Real-world cost-minimization analysis	CHEERS 2022 + ISPOR	Economic or resource-use evaluation was the report's primary analytic purpose; reporting completeness was assessed.	Included report; no parent or companion article substituted.	Reporting completeness only; no exclusion or weighting.
Gower et al., 2025 [58]	Economic evaluation/modelling	Comparative economic evaluation across service models	CHEERS 2022 + ISPOR	Economic or resource-use evaluation was the report's primary analytic purpose; reporting completeness was assessed.	Included report; no parent or companion article substituted.	Reporting completeness only; no exclusion or weighting.
Vaughan-Graham et al., 2020 [59]	Qualitative	Qualitative interview study	CASP qualitative	Qualitative inquiry was the primary design and source of implementation evidence.	Included report; no parent or companion article substituted.	Context only; no exclusion or weighting.
Hotz et al., 2024 [60]	Mixed methods	Mixed-methods feasibility study	MMAT	Qualitative and quantitative components were integrated within one mixed-methods design.	Included report; no parent or companion article substituted.	Context only; no exclusion or weighting.
Pinto et al., 2020 [61]	Economic evaluation/modelling	Budget-impact analysis	CHEERS 2022 + ISPOR	Economic or resource-use evaluation was the report's primary analytic purpose; reporting completeness was assessed.	Included report; no parent or companion article substituted.	Reporting completeness only; no exclusion or weighting.
Thomassen et al., 2019 [62]	Qualitative	Qualitative interview study	CASP qualitative	Qualitative inquiry was the primary design and source of implementation evidence.	Included report; no parent or companion article substituted.	Context only; no exclusion or weighting.
Bustamante Valles et al., 2016 [63]	Randomized trial	Pilot randomized controlled trial	RoB 2	Randomized allocation used to compare intervention conditions.	Included report; no parent or companion article substituted.	Context only; no exclusion or weighting.
Masiero et al., 2014 [64]	Economic evaluation/modelling	Trial-based treatment-cost evaluation	CHEERS 2022 + ISPOR	Economic or resource-use evaluation was the report's primary analytic purpose; reporting completeness was assessed.	Included report; no parent or companion article substituted.	Reporting completeness only; no exclusion or weighting.

Study	Table 1 design category	Study-specific design	Primary instrument	**Instrument-selection rationale**	Appraisal unit	**Role in synthesis**
Aguirre-Ollinger et al., 2024 [65]	Quasi-experimental	Single-group clinic-to-home feasibility study	JBI quasi-experimental	Non-randomized intervention evaluation without the comparative intervention-effect structure assigned to ROBINS-I.	Included report; no parent or companion article substituted.	Context only; no exclusion or weighting.
Maggio et al., 2024 [66]	Case series	Retrospective pathway case series	JBI case series	Descriptive clinical series without a comparator group.	Included report; no parent or companion article substituted.	Context only; no exclusion or weighting.
Olimb Hillkirk et al., 2025 [67]	Qualitative	Qualitative exploratory study	CASP qualitative	Qualitative inquiry was the primary design and source of implementation evidence.	Included report; no parent or companion article substituted.	Context only; no exclusion or weighting.
Klaic et al., 2024 [68]	Analytical cross-sectional	Cross-sectional clinician survey	JBI analytical cross-sectional	Cross-sectional survey or analysis of practitioner, patient or service characteristics.	Included report; no parent or companion article substituted.	Context only; no exclusion or weighting.
Li et al., 2021 [69]	Mixed methods	Mixed-methods survey	MMAT	Qualitative and quantitative components were integrated within one mixed-methods design.	Included report; no parent or companion article substituted.	Context only; no exclusion or weighting.
Luciani et al., 2023 [70]	Analytical cross-sectional	Cross-sectional therapist survey	JBI analytical cross-sectional	Cross-sectional survey or analysis of practitioner, patient or service characteristics.	Included report; no parent or companion article substituted.	Context only; no exclusion or weighting.
Nicora et al., 2025 [71]	Analytical cross-sectional	National cross-sectional practitioner survey	JBI analytical cross-sectional	Cross-sectional survey or analysis of practitioner, patient or service characteristics.	Included report; no parent or companion article substituted.	Context only; no exclusion or weighting.
Flynn et al., 2019 [72]	Qualitative	Qualitative interview study	CASP qualitative	Qualitative inquiry was the primary design and source of implementation evidence.	Included report; no parent or companion article substituted.	Context only; no exclusion or weighting.
Flynn et al., 2022 [73]	Quasi-experimental	Pre-post observational implementation study	JBI quasi-experimental	Non-randomized intervention evaluation without the comparative intervention-effect structure assigned to ROBINS-I.	Included report; no parent or companion article substituted.	Context only; no exclusion or weighting.
Høyer et al., 2022 [74]	Quasi-experimental	Exploratory single-group feasibility study	JBI quasi-experimental	Non-randomized intervention evaluation without the comparative intervention-effect structure assigned to ROBINS-I.	Included report; no parent or companion article substituted.	Context only; no exclusion or weighting.
Pavan et al., 2024 [75]	Case series	Retrospective clinical case series	JBI case series	Descriptive clinical series without a comparator group.	Included report; no parent or companion article substituted.	Context only; no exclusion or weighting.
Swank et al., 2020 [76]	Case series	Prospective feasibility case series	JBI case series	Descriptive clinical series without a comparator group.	Included report; no parent or companion article substituted.	Context only; no exclusion or weighting.
Swank et al., 2020 [77]	Case series	Single-centre retrospective case series	JBI case series	Descriptive clinical series without a comparator group.	Included report; no parent or companion article substituted.	Context only; no exclusion or weighting.
Schuster-Amft et al., 2022 [78]	Quasi-experimental	Multicentre single-group feasibility study	JBI quasi-experimental	Non-randomized intervention evaluation without the comparative intervention-effect structure assigned to ROBINS-I.	Included report; no parent or companion article substituted.	Context only; no exclusion or weighting.
Devittori et al., 2024 [79]	Quasi-experimental	Single-group interventional feasibility study	JBI quasi-experimental	Non-randomized intervention evaluation without the comparative intervention-effect structure assigned to ROBINS-I.	Included report; no parent or companion article substituted.	Context only; no exclusion or weighting.
Johnson et al., 2017 [80]	Quality improvement/service evaluation	Multi-site service-development report	QI-MQCS	Quality-improvement or service-model report centred on local implementation change.	Included report; no parent or companion article substituted.	Context only; no exclusion or weighting.
Arnold et al., 2024 [81]	Case series	Retrospective case series	JBI case series	Descriptive clinical series without a comparator group.	Included report; no parent or companion article substituted.	Context only; no exclusion or weighting.
van Dellen et al., 2025 [82]	Mixed methods	Mixed-methods development and feasibility study	MMAT	Qualitative and quantitative components were integrated within one mixed-methods design.	Included report; no parent or companion article substituted.	Context only; no exclusion or weighting.
Fundarò et al., 2023 [83]	Quasi-experimental	Multicentre observational pre-post study	JBI quasi-experimental	Non-randomized intervention evaluation without the comparative intervention-effect structure assigned to ROBINS-I.	Included report; no parent or companion article substituted.	Context only; no exclusion or weighting.

Study	Table 1 design category	Study-specific design	Primary instrument	**Instrument-selection rationale**	Appraisal unit	**Role in synthesis**
Mensah-Gourmel et al., 2024 [84]	Analytical cross-sectional	Cross-sectional professional survey	JBI analytical cross-sectional	Cross-sectional survey or analysis of practitioner, patient or service characteristics.	Included report; no parent or companion article substituted.	Context only; no exclusion or weighting.
Kang et al., 2016 [85]	Analytical cross-sectional	Multi-site cross-sectional professional survey	JBI analytical cross-sectional	Cross-sectional survey or analysis of practitioner, patient or service characteristics.	Included report; no parent or companion article substituted.	Context only; no exclusion or weighting.
Patarini et al., 2024 [86]	Quasi-experimental	Within-participant non-randomized experimental study	JBI quasi-experimental	Non-randomized intervention evaluation without the comparative intervention-effect structure assigned to ROBINS-I.	Included report; no parent or companion article substituted.	Context only; no exclusion or weighting.
Reichl et al., 2020 [87]	Cohort	Retrospective cohort study	JBI cohort	Longitudinal observational cohort reporting programme exposure and subsequent outcomes.	Included report; no parent or companion article substituted.	Context only; no exclusion or weighting.
Huang et al., 2018 [88]	Non-randomized comparative intervention	Non-randomized comparative intervention study	ROBINS-I	Explicit non-randomized intervention-effect comparison between groups or defined service periods.	Included report; no parent or companion article substituted.	Context only; no exclusion or weighting.
Calabrò et al., 2020 [89]	Quality improvement/service evaluation	Regional service-model report	QI-MQCS	Quality-improvement or service-model report centred on local implementation change.	Included report; no parent or companion article substituted.	Context only; no exclusion or weighting.
De Cola et al., 2023 [90]	Non-randomized comparative intervention	Retrospective controlled before-after service evaluation	ROBINS-I	Explicit non-randomized intervention-effect comparison between groups or defined service periods.	Included report; no parent or companion article substituted.	Context only; no exclusion or weighting.
Ielo et al., 2024 [91]	Non-randomized comparative intervention	Retrospective controlled before-after service evaluation	ROBINS-I	Explicit non-randomized intervention-effect comparison between groups or defined service periods.	Included report; no parent or companion article substituted.	Context only; no exclusion or weighting.
Manuli et al., 2020 [92]	Quasi-experimental	Exploratory single-group pre-post study	JBI quasi-experimental	Non-randomized intervention evaluation without the comparative intervention-effect structure assigned to ROBINS-I.	Included report; no parent or companion article substituted.	Context only; no exclusion or weighting.
Fundarò et al., 2026 [93]	Analytical cross-sectional	Multicentre cross-sectional therapist survey	JBI analytical cross-sectional	Cross-sectional survey or analysis of practitioner, patient or service characteristics.	Included report; no parent or companion article substituted.	Context only; no exclusion or weighting.
Hesse et al., 2014 [94]	Randomized trial	Single-blind randomized controlled trial	RoB 2	Randomized allocation used to compare intervention conditions.	Included report; no parent or companion article substituted.	Context only; no exclusion or weighting.
Nedergård et al., 2023 [95]	Qualitative	Qualitative interview study nested in a training programme	CASP qualitative	Qualitative inquiry was the primary design and source of implementation evidence.	Included report; no parent or companion article substituted.	Context only; no exclusion or weighting.
Ouendi et al., 2024 [96]	Mixed methods	Mixed-methods clinician study	MMAT	Qualitative and quantitative components were integrated within one mixed-methods design.	Included report; no parent or companion article substituted.	Context only; no exclusion or weighting.
Phelan et al., 2015 [97]	Qualitative	Interpretivist qualitative study	CASP qualitative	Qualitative inquiry was the primary design and source of implementation evidence.	Included report; no parent or companion article substituted.	Context only; no exclusion or weighting.
Stephenson et al., 2018 [98]	Qualitative	Qualitative interview study	CASP qualitative	Qualitative inquiry was the primary design and source of implementation evidence.	Included report; no parent or companion article substituted.	Context only; no exclusion or weighting.
Mazzoleni et al., 2017 [99]	Case series	Multicentre retrospective uncontrolled case series	JBI case series	Descriptive clinical series without a comparator group.	Included report; no parent or companion article substituted.	Context only; no exclusion or weighting.
Maggio et al., 2024 [100]	Cohort	Retrospective cohort study	JBI cohort	Longitudinal observational cohort reporting programme exposure and subsequent outcomes.	Included report; no parent or companion article substituted.	Context only; no exclusion or weighting.

Note. The table contains one row for each of the 55 included reports. Every row corresponds to the same report in Supplementary Tables S3 and S7; no parent or companion article was used as the appraisal unit, and no report was appraised twice. Tool-specific judgments retained their original scales and were not converted to a common score.

Abbreviations: CASP, Critical Appraisal Skills Programme; CHEERS, Consolidated Health Economic Evaluation Reporting Standards; ISPOR, International Society for Pharmacoeconomics and Outcomes Research; JBI, Joanna Briggs Institute; MMAT, Mixed Methods Appraisal Tool; QI-MQCS, Quality Improvement Minimum Quality Criteria Set; RoB 2, Risk of Bias 2; ROBINS-I, Risk Of Bias In Non-randomized Studies of Interventions.

Supplementary Table S7. Study-level design-appropriate appraisal results

Study	Design	Instrument	Domain-level judgments	Overall appraisal	Study-specific rationale for the overall judgment
Cao et al., 2021 [46]	Quality-improvement initiative	QI-MQCS	Organizational motivation/context: Reported; Intervention description: Reported; Study design/comparator: No concurrent comparator; Data source/denominator: Reported; Timeline/adherence: Partial; Health outcomes/penetration: Partial; Limitations: Reported	Some limitations	The initiative defined its local aim, intervention, time series and routine data source, but lacked a concurrent comparator and was vulnerable to secular change.
Flynn et al., 2025 [47]	Qualitative focus-group study with embedded usability survey	CASP qualitative	Aims/methodology: Yes; Design/recruitment: Mostly; Data collection: Yes; Researcher-participant relationship/reflexivity: Unclear; Ethics: Yes; Analysis/findings: Mostly; Value/transferability: Context dependent	Some limitations	Focus groups addressed the implementation question directly; the small discipline-specific sample and limited reflexivity constrained transferability.
Gillespie et al., 2023 [48]	Retrospective descriptive case series with therapist survey	JBI case series	Inclusion criteria: Yes; Condition/measurement: Yes; Consecutive/complete inclusion: Unclear; Participant/setting reporting: Yes; Outcome/follow-up reporting: Mostly; Statistical analysis: Descriptive/appropriate	Some limitations	The clinical records and therapist survey described routine use, but retrospective inclusion of treated patients and incomplete standardization limited inference.
Heinemann et al., 2018 [49]	Multicentre qualitative focus-group study	CASP qualitative	Aims/methodology: Yes; Design/recruitment: Yes; Data collection: Yes; Researcher-participant relationship/reflexivity: Unclear; Ethics: Yes; Analysis/findings: Yes; Value/transferability: Context dependent	Meets most criteria	Multicentre focus groups, verbatim capture and systematic thematic analysis supported credibility; local early-adopter contexts remained relevant.
Pila et al., 2022 [50]	Retrospective non-randomized comparative intervention study	ROBINS-I	D1 Bias due to confounding: Serious; D2 Bias in selection of participants: Serious; D3 Bias in classification of interventions: Low; D4 Bias due to deviations from intended interventions: Moderate; D5 Bias due to missing data: Low; D6 Bias in measurement of outcomes: Moderate; D7 Bias in selection of the reported result: Moderate	Serious	The two dose groups came from different care contexts, including a previous trial cohort, creating serious confounding and selection concerns.
Read et al., 2020 [51]	Exploratory qualitative interview study	CASP qualitative	Aims/methodology: Yes; Design/recruitment: Mostly; Data collection: Yes; Researcher-participant relationship/reflexivity: Unclear; Ethics: Yes; Analysis/findings: Mostly; Value/transferability: Context dependent	Some limitations	Interviews provided relevant experiential data, but the three-participant single-centre sample and limited reflexive reporting reduced transferability.
Warmbein et al., 2023 [52]	Exploratory qualitative interview study	CASP qualitative	Aims/methodology: Yes; Design/recruitment: Yes; Data collection: Yes; Researcher-participant relationship/reflexivity: Unclear; Ethics: Yes; Analysis/findings: Yes; Value/transferability: Context dependent	Meets most criteria	The study used a stated qualitative design, purposive expert sampling, semi-structured interviews and COREQ-informed reporting; transferability remained contextual.
Buschfort et al., 2010 [53]	Open single-group pre-post study	JBI quasi-experimental	Cause/effect and temporality: Yes; Comparable participants/control: No concurrent control; Multiple measurements: Yes; Follow-up completeness: Mostly; Outcome measurement: Yes; Statistical analysis: Mostly	Some limitations	The open pre-post design documented dose, utilization and acceptance, but lacked a concurrent control and included only subsets in outcome analyses.
Aprile et al., 2019 [54]	Single-group feasibility study	JBI quasi-experimental	Cause/effect and temporality: Yes; Comparable participants/control: No concurrent control; Multiple measurements: Yes; Follow-up completeness: Mostly; Outcome measurement: Yes; Statistical analysis: Mostly	Some limitations	The multidisciplinary device-selection process and feasibility procedures were described, but the small uncontrolled pilot could not separate intervention from context.
Aprile et al., 2019 [55]	Observational single-group feasibility study	JBI quasi-experimental	Cause/effect and temporality: Yes; Comparable participants/control: No concurrent control; Multiple measurements: Yes; Follow-up completeness: Mostly; Outcome measurement: Yes; Statistical analysis: Yes	Meets most criteria	The organizational model, denominator, observation window and service metrics were clearly reported; lack of a comparator limited causal interpretation.
Gagnon et al., 2018 [56]	Prospective single-group feasibility case series	JBI case series	Inclusion criteria: Yes; Condition/measurement: Yes; Consecutive/complete inclusion: Unclear; Participant/setting reporting: Yes; Outcome/follow-up reporting: Mostly; Statistical analysis: Descriptive/appropriate	Some limitations	Recruitment, attendance, learnability and safety were prospectively described, but the small selected sample limited precision and generalizability.
Gower et al., 2024 [57]	Real-world cost-minimization analysis	CHEERS 2022 + ISPOR	Perspective/comparator: Reported; Time horizon/discounting: Reported or not applicable; Resource use/unit costs: Reported; Outcomes/valuation: Reported; Uncertainty/sensitivity: Reported; Distributional/transferability issues: Partial	Substantially reported	Perspective, alternatives, resource inputs and staffing assumptions were reported, with scenario analysis; transferability depended on Italian prices and utilization.
Gower et al., 2025 [58]	Comparative economic evaluation across service models	CHEERS 2022 + ISPOR	Perspective/comparator: Reported; Time horizon/discounting: Reported or not applicable; Resource use/unit costs: Reported; Outcomes/valuation: Reported; Uncertainty/sensitivity: Reported; Distributional/transferability issues: Partial	Substantially reported	The comparative costing made organizational assumptions explicit and explored multiple healthcare models; model inputs remained context dependent.
Vaughan-Graham et al., 2020 [59]	Qualitative interview study	CASP qualitative	Aims/methodology: Yes; Design/recruitment: Yes; Data collection: Yes; Researcher-participant relationship/reflexivity: Unclear; Ethics: Yes; Analysis/findings: Yes; Value/transferability: Context dependent	Meets most criteria	The qualitative design aligned with stakeholder-experience questions and supported themes with participant accounts; the single exposure context limited transferability.
Hotz et al., 2024 [60]	Mixed-methods feasibility study	MMAT	Clear mixed-methods rationale: Yes; Qualitative component: Adequate; Quantitative component: Adequate; Integration: Adequate; Divergence/design adherence: Addressed	Meets most criteria	Quantitative feasibility data and qualitative experience data were both reported and integrated, although the single-centre sample limited external validity.
Pinto et al., 2020 [61]	Budget-impact analysis	CHEERS 2022 + ISPOR	Perspective/comparator: Reported; Time horizon/discounting: Reported or not applicable; Resource use/unit costs: Reported; Outcomes/valuation: Reported; Uncertainty/sensitivity: Reported; Distributional/transferability issues: Partial	Substantially reported	The budget-impact perspective, population, resource categories and utilization scenarios were stated; estimates depended on local implementation assumptions.
Thomassen et al., 2019 [62]	Qualitative interview study	CASP qualitative	Aims/methodology: Yes; Design/recruitment: Mostly; Data collection: Yes; Researcher-participant relationship/reflexivity: Unclear; Ethics: Yes; Analysis/findings: Mostly; Value/transferability: Context dependent	Some limitations	Participant perspectives were appropriate to the question, but the small setting-specific sample and limited reflexivity restricted transferability.
Bustamante Valles et al., 2016 [63]	Pilot randomized controlled trial	RoB 2	D1 Randomization process: Some concerns; D2 Deviations from intended interventions: Some concerns; D3 Missing outcome data: High; D4 Measurement of the outcome: Some concerns; D5 Selection of the reported result: Some concerns	High	Sequence generation was described, but allocation concealment was unclear, baseline age differed, seven recruited participants did not complete, assessors were not clearly blinded, and no prespecified analysis plan was available.
Masiero et al., 2014 [64]	Trial-based treatment-cost evaluation	CHEERS 2022 + ISPOR	Perspective/comparator: Partial; Time horizon/discounting: Unclear; Resource use/unit costs: Reported; Outcomes/valuation: Partial; Uncertainty/sensitivity: Limited; Distributional/transferability issues: Partial	Partially reported	Treatment-cost inputs and the service comparison were reported, but perspective, uncertainty analysis and longer-term consequences were incompletely specified.
Aguirre-Ollinger et al., 2024 [65]	Single-group clinic-to-home feasibility study	JBI quasi-experimental	Cause/effect and temporality: Yes; Comparable participants/control: No concurrent control; Multiple measurements: Yes; Follow-up completeness: Mostly; Outcome measurement: Yes; Statistical analysis: Mostly	Some limitations	The clinic-to-home pathway, adherence and feasibility outcomes were reported, but the uncontrolled selected sample limited causal and comparative inference.
Maggio et al., 2024 [66]	Retrospective pathway case series	JBI case series	Inclusion criteria: Yes; Condition/measurement: Yes; Consecutive/complete inclusion: Unclear; Participant/setting reporting: Yes; Outcome/follow-up reporting: Mostly; Statistical analysis: Descriptive/appropriate	Some limitations	The pathway and multidisciplinary service were described retrospectively; patient selection and absence of a comparator limited inference.
Olimb Hillkirk et al., 2025 [67]	Qualitative exploratory study	CASP qualitative	Aims/methodology: Yes; Design/recruitment: Yes; Data collection: Yes; Researcher-participant relationship/reflexivity: Unclear; Ethics: Yes; Analysis/findings: Yes; Value/transferability: Context dependent	Meets most criteria	The exploratory qualitative approach, sampling and analysis were aligned with user-acceptance questions, with remaining limitations in setting-specific transferability.
Klaic et al., 2024 [68]	Cross-sectional clinician survey	JBI analytical cross-sectional	Inclusion criteria/sample: Yes; Setting/participants: Yes; Exposure/outcome measurement: Yes; Confounding: Partial; Statistical analysis: Yes; Response/non-response: Unclear	Meets most criteria	The sampling frame, technology-acceptance constructs and analyses were clearly described; response and self-selection bias remained possible.
Li et al., 2021 [69]	Mixed-methods survey	MMAT	Clear mixed-methods rationale: Partial; Qualitative component: Adequate; Quantitative component: Some limitations; Integration: Partial; Divergence/design adherence: Unclear	Some limitations	Quantitative and qualitative survey components addressed the same implementation question, but response representativeness and depth of integration were limited.
Luciani et al., 2023 [70]	Cross-sectional therapist survey	JBI analytical cross-sectional	Inclusion criteria/sample: Partial; Setting/participants: Yes; Exposure/outcome measurement: Mostly; Confounding: Partial; Statistical analysis: Yes; Response/non-response: Unclear	Some limitations	The survey used a defined acceptance model, but convenience sampling and hypothetical judgments about device use limited representativeness.
Nicora et al., 2025 [71]	National cross-sectional practitioner survey	JBI analytical cross-sectional	Inclusion criteria/sample: Yes; Setting/participants: Yes; Exposure/outcome measurement: Yes; Confounding: Partial; Statistical analysis: Yes; Response/non-response: Unclear	Meets most criteria	The national sampling frame, instrument development and reporting were comparatively strong; voluntary response may still have selected technology-interested practitioners.
Flynn et al., 2019 [72]	Qualitative interview study	CASP qualitative	Aims/methodology: Yes; Design/recruitment: Yes; Data collection: Yes; Researcher-participant relationship/reflexivity: Unclear; Ethics: Yes; Analysis/findings: Yes; Value/transferability: Context dependent	Meets most criteria	The interview study directly examined routine-practice introduction and used systematic analysis; views were drawn from one service context.
Flynn et al., 2022 [73]	Pre-post observational implementation study	JBI quasi-experimental	Cause/effect and temporality: Yes; Comparable participants/control: No concurrent control; Multiple measurements: Yes; Follow-up completeness: Mostly; Outcome measurement: Yes; Statistical analysis: Mostly	Some limitations	Routine practice before-after data quantified dose and duration, but the uncontrolled design and clinical selection limited attribution.
Høyer et al., 2022 [74]	Exploratory single-group feasibility study	JBI quasi-experimental	Cause/effect and temporality: Yes; Comparable participants/control: No concurrent control; Multiple measurements: Yes; Follow-up completeness: Mostly; Outcome measurement: Yes; Statistical analysis: Mostly	Some limitations	Feasibility, functional outcomes and patient experiences were reported prospectively; no concurrent comparator was used.
Pavan et al., 2024 [75]	Retrospective clinical case series	JBI case series	Inclusion criteria: Yes; Condition/measurement: Yes; Consecutive/complete inclusion: Unclear; Participant/setting reporting: Yes; Outcome/follow-up reporting: Mostly; Statistical analysis: Descriptive/appropriate	Some limitations	The retrospective sample and protocol were described, but selection of treated patients and lack of a comparator limited inference.
Swank et al., 2020 [76]	Prospective feasibility case series	JBI case series	Inclusion criteria: Yes; Condition/measurement: Yes; Consecutive/complete inclusion: Unclear; Participant/setting reporting: Yes; Outcome/follow-up reporting: Mostly; Statistical analysis: Descriptive/appropriate	Some limitations	The feasibility study reported screening, delivery and safety, but the small selected sample and absence of a comparator limited precision.
Swank et al., 2020 [77]	Single-centre retrospective case series	JBI case series	Inclusion criteria: Yes; Condition/measurement: Yes; Consecutive/complete inclusion: Unclear; Participant/setting reporting: Yes; Outcome/follow-up reporting: Mostly; Statistical analysis: Descriptive/appropriate	Some limitations	Routine exoskeleton utilization was described from records, but retrospective inclusion and incomplete control of case mix limited inference.
Schuster-Amft et al., 2022 [78]	Multicentre single-group feasibility study	JBI quasi-experimental	Cause/effect and temporality: Yes; Comparable participants/control: No concurrent control; Multiple measurements: Yes; Follow-up completeness: Mostly; Outcome measurement: Yes; Statistical analysis: Mostly	Some limitations	The multicentre intensive programme reported feasibility and costs, but the single-group design and site heterogeneity limited causal interpretation.
Devittori et al., 2024 [79]	Single-group interventional feasibility study	JBI quasi-experimental	Cause/effect and temporality: Yes; Comparable participants/control: No concurrent control; Multiple measurements: Yes; Follow-up completeness: Mostly; Outcome measurement: Yes; Statistical analysis: Yes	Meets most criteria	The staged autonomy protocol, feasibility outcomes, dose and user experience were clearly reported; the single-group design limited comparative inference.
Johnson et al., 2017 [80]	Multi-site service-development report	QI-MQCS	Organizational motivation/context: Reported; Intervention description: Partial; Study design/comparator: No concurrent comparator; Data source/denominator: Partial; Timeline/adherence: Partial; Health outcomes/penetration: Partial; Limitations: Limited	Major limitations	The report described programme evolution across settings but did not provide a uniform evaluative design, sampling frame or complete outcome dataset.
Arnold et al., 2024 [81]	Retrospective case series	JBI case series	Inclusion criteria: Yes; Condition/measurement: Yes; Consecutive/complete inclusion: Unclear; Participant/setting reporting: Yes; Outcome/follow-up reporting: Mostly; Statistical analysis: Descriptive/appropriate	Some limitations	The retrospective continuum-of-care sample described delivery patterns, but treated-patient selection and incomplete adjustment limited transferability.
van Dellen et al., 2025 [82]	Mixed-methods development and feasibility study	MMAT	Clear mixed-methods rationale: Partial; Qualitative component: Adequate; Quantitative component: Some limitations; Integration: Partial; Divergence/design adherence: Unclear	Some limitations	The study combined app development, clinician input and patient feasibility data; component integration and sampling were only partly specified.
Fundarò et al., 2023 [83]	Multicentre observational pre-post study	JBI quasi-experimental	Cause/effect and temporality: Yes; Comparable participants/control: No concurrent control; Multiple measurements: Yes; Follow-up completeness: Mostly; Outcome measurement: Yes; Statistical analysis: Mostly	Some limitations	Instrument development and multicentre implementation were described, but the pilot sample and uncontrolled pre-post structure limited inference.
Mensah-Gourmel et al., 2024 [84]	Cross-sectional professional survey	JBI analytical cross-sectional	Inclusion criteria/sample: Partial; Setting/participants: Yes; Exposure/outcome measurement: Mostly; Confounding: Partial; Statistical analysis: Yes; Response/non-response: Unclear	Some limitations	The multinational survey documented access and use, but voluntary participation, heterogeneous respondents and self-report limited representativeness.
Kang et al., 2016 [85]	Multi-site cross-sectional professional survey	JBI analytical cross-sectional	Inclusion criteria/sample: Partial; Setting/participants: Yes; Exposure/outcome measurement: Mostly; Confounding: Partial; Statistical analysis: Yes; Response/non-response: Unclear	Some limitations	The multi-hospital survey addressed clinician views with a defined questionnaire; sampling and non-response information were incomplete.

Study	Design	Instrument	Domain-level judgments	Overall appraisal	Study-specific rationale for the overall judgment
Patarini et al., 2024 [86]	Within-participant non-randomized experimental study	JBI quasi-experimental	Cause/effect and temporality: Yes; Comparable participants/control: No concurrent control; Multiple measurements: Yes; Follow-up completeness: Mostly; Outcome measurement: Yes; Statistical analysis: Mostly	Some limitations	Within-participant measurement supported the mechanistic comparison, but the small selected sample, non-randomized conditions and setting limited implementation inference.
Reichl et al., 2020 [87]	Retrospective cohort study	JBI cohort	Group definition: Yes; Exposure measurement: Yes; Confounding identification/control: Partial; Outcome measurement: Yes; Follow-up/completeness: Mostly; Statistical analysis: Mostly	Some limitations	The cohort and routine programme were clearly described, but retrospective analysis and absence of a concurrent comparator limited attribution.
Huang et al., 2018 [88]	Non-randomized comparative intervention study	ROBINS-I	D1 Bias due to confounding: Serious; D2 Bias in selection of participants: Serious; D3 Bias in classification of interventions: Low; D4 Bias due to deviations from intended interventions: Moderate; D5 Bias due to missing data: Moderate; D6 Bias in measurement of outcomes: Moderate; D7 Bias in selection of the reported result: Moderate	Serious	Clinical-service and laboratory-trial groups differed in setting and scheduling, with limited adjustment for baseline and contextual confounding.
Calabrò et al., 2020 [89]	Regional service-model report	QI-MQCS	Organizational motivation/context: Reported; Intervention description: Partial; Study design/comparator: No concurrent comparator; Data source/denominator: Partial; Timeline/adherence: Partial; Health outcomes/penetration: Partial; Limitations: Limited	Major limitations	The hub-and-spoke model and rationale were described, but the report did not use a complete evaluative design or provide a comparative outcome dataset.
De Cola et al., 2023 [90]	Retrospective controlled before-after service evaluation	ROBINS-I	D1 Bias due to confounding: Serious; D2 Bias in selection of participants: Moderate; D3 Bias in classification of interventions: Low; D4 Bias due to deviations from intended interventions: Moderate; D5 Bias due to missing data: Moderate; D6 Bias in measurement of outcomes: Low; D7 Bias in selection of the reported result: Moderate	Serious	Administrative outcomes before and after network activation were measured consistently, but secular changes and other system interventions could not be separated.
Ielo et al., 2024 [91]	Retrospective controlled before-after service evaluation	ROBINS-I	D1 Bias due to confounding: Serious; D2 Bias in selection of participants: Moderate; D3 Bias in classification of interventions: Low; D4 Bias due to deviations from intended interventions: Moderate; D5 Bias due to missing data: Moderate; D6 Bias in measurement of outcomes: Low; D7 Bias in selection of the reported result: Moderate	Serious	Before-after administrative indicators were clearly defined, but residual confounding, selection and concurrent regional changes limited causal attribution.
Manuli et al., 2020 [92]	Exploratory single-group pre-post study	JBI quasi-experimental	Cause/effect and temporality: Yes; Comparable participants/control: No concurrent control; Multiple measurements: Yes; Follow-up completeness: Mostly; Outcome measurement: Yes; Statistical analysis: Mostly	Some limitations	Feasibility and usability were measured in a defined pathway, but the selected single-group pre-post sample lacked a comparator.
Fundarò et al., 2026 [93]	Multicentre cross-sectional therapist survey	JBI analytical cross-sectional	Inclusion criteria/sample: Partial; Setting/participants: Yes; Exposure/outcome measurement: Mostly; Confounding: Partial; Statistical analysis: Yes; Response/non-response: Unclear	Some limitations	The multicentre survey and questionnaire were described, but voluntary response, instrument validation and non-response handling remained incomplete.
Hesse et al., 2014 [94]	Single-blind randomized controlled trial	RoB 2	D1 Randomization process: Some concerns; D2 Deviations from intended interventions: Low; D3 Missing outcome data: Low; D4 Measurement of the outcome: Low; D5 Selection of the reported result: Some concerns	Some concerns	Restricted randomization and blinded primary-outcome assessment were reported; concealment and availability of a prespecified analysis plan were insufficiently detailed.
Nedergård et al., 2023 [95]	Qualitative interview study nested in a training programme	CASP qualitative	Aims/methodology: Yes; Design/recruitment: Yes; Data collection: Yes; Researcher-participant relationship/reflexivity: Unclear; Ethics: Yes; Analysis/findings: Yes; Value/transferability: Context dependent	Meets most criteria	The qualitative study used semi-structured interviews and content analysis to capture participant experience; transferability was bounded by the intervention context.
Ouendi et al., 2024 [96]	Mixed-methods clinician study	MMAT	Clear mixed-methods rationale: Yes; Qualitative component: Adequate; Quantitative component: Adequate; Integration: Adequate; Divergence/design adherence: Addressed	Meets most criteria	Interview findings and SUS data were collected in parallel and interpreted together; sampling remained limited to users at participating services.
Phelan et al., 2015 [97]	Interpretivist qualitative study	CASP qualitative	Aims/methodology: Yes; Design/recruitment: Yes; Data collection: Yes; Researcher-participant relationship/reflexivity: Unclear; Ethics: Yes; Analysis/findings: Yes; Value/transferability: Context dependent	Meets most criteria	The interpretivist design and child-centred methods matched the question and supported analytic depth; the small purposive sample limited transferability.
Stephenson et al., 2018 [98]	Qualitative interview study	CASP qualitative	Aims/methodology: Yes; Design/recruitment: Mostly; Data collection: Yes; Researcher-participant relationship/reflexivity: Unclear; Ethics: Yes; Analysis/findings: Mostly; Value/transferability: Context dependent	Some limitations	Interviews addressed therapist experience and generated relevant themes, but the small single-centre sample and reflexive detail were limited.
Mazzoleni et al., 2017 [99]	Multicentre retrospective uncontrolled case series	JBI case series	Inclusion criteria: Yes; Condition/measurement: Yes; Consecutive/complete inclusion: Unclear; Participant/setting reporting: Yes; Outcome/follow-up reporting: Mostly; Statistical analysis: Descriptive/appropriate	Some limitations	The multicentre retrospective series described intervention and outcomes, but uncontrolled treated-patient selection and missing confounder adjustment limited inference.
Maggio et al., 2024 [100]	Retrospective cohort study	JBI cohort	Group definition: Yes; Exposure measurement: Yes; Confounding identification/control: Partial; Outcome measurement: Yes; Follow-up/completeness: Mostly; Statistical analysis: Mostly	Some limitations	The retrospective pathway cohort reported pre-post outcomes and service features, but selection, confounding and lack of a comparator limited attribution.

Note. One row is presented for each of the 55 included reports, with a corresponding entry in Supplementary Table S3. No parent or companion article was substituted as an appraisal unit, and no report was assessed twice. RoB 2 and ROBINS-I entries represent risk-of-bias judgments; JBI, CASP, MMAT and QI-MQCS entries describe design-specific methodological appraisal; CHEERS/ISPOR entries describe reporting completeness. Categories are not interchangeable across tools and were not used for exclusion or quantitative weighting.

Abbreviations: CASP, Critical Appraisal Skills Programme; CHEERS, Consolidated Health Economic Evaluation Reporting Standards; ISPOR, International Society for Pharmacoeconomics and Outcomes Research; JBI, Joanna Briggs Institute; MMAT, Mixed Methods Appraisal Tool; QI-MQCS, Quality Improvement Minimum Quality Criteria Set; RoB 2, Risk of Bias 2; ROBINS-I, Risk Of Bias In Non-randomized Studies of Interventions.

Supplementary Table S8. Subgroup mapping by robot type, setting, and implementation phase

Subgroup	Main implementation emphasis	Common bottleneck	Interpretive note
Upper-limb robotics	Practice dose, group supervision, usability, workflow efficiency	Device access, training, single-device scheduling, documentation	Often more compatible with semi-supervised or circuit models.
Gait robotics	Dose, walking practice intensity, progression metrics	Setup, patient tolerance, therapist time, fixed session durations	Requires careful integration into routine physiotherapy schedules.
Overground exoskeletons	Screening, safety, staffing, patient fit, device metrics	High supervision intensity, eligibility constraints, certification	Implementation strongly depends on staff competency and safety governance.
Multi-technology pathways	Personalisation, multi-device allocation, pathway coordination	Infrastructure, device availability, attribution of effects	Requires programme-level governance and reporting.
Inpatient setting	Fixed therapy schedules, medically complex patients, staffing	Setup time and competing rehabilitation priorities	Workflow compatibility is central.
Community/home or hybrid	Adherence, telemonitoring, caregiver involvement	Logistics, home environment, technical support	Requires transition criteria and remote monitoring.
Network or system-level	Access, spread, patient mobility, regional costs	Governance, reimbursement, staff training across sites	Implementation outcomes should include penetration and sustainability.

Note. Subgroup patterns describe the distribution of reported implementation content and should not be interpreted as comparative effect estimates.
Abbreviations: OEGT, overground exoskeleton gait training; RAGT, robot-assisted gait training; RR, rehabilitation robotics; t-RAGT, treadmill-based robot-assisted gait training.

Supplementary Table S9. Determinant-strategy-outcome matrix

Determinant cluster	CFIR interpretation	Common ERIC-mapped strategies	Co-reported implementation and service outcomes	Implementation hypothesis for future testing
Staffing and supervision constraints	Inner Setting: available resources; structural characteristics	Revise roles; train staff; introduce assistants or group supervision; create device schedules	Feasibility, adoption, implementation cost	Alternative supervision ratios may improve adoption and cost if safety and fidelity are preserved.
Setup, calibration, and workflow burden	Intervention Characteristics: complexity; Inner Setting: compatibility	Workflow redesign; standard operating procedures; co-location; audit setup time	Feasibility, fidelity, adoption	Reducing setup burden may increase delivered dose within fixed therapy time.
Clinician confidence and competency	Characteristics of Individuals; Inner Setting: access to knowledge	Training, competency checks, super-users, refresher sessions, technical assistance	Acceptability, appropriateness, adoption	Competency-based training may improve confidence and routine uptake.
Eligibility and patient fit	Characteristics of Individuals; Intervention Characteristics: adaptability	Screening protocols, progression rules, patient selection criteria	Appropriateness, feasibility, safety	Explicit eligibility criteria may improve safe targeting and reduce inefficient use.
Safety governance requirements	Process: planning/executing; Inner Setting: readiness	Adverse-event monitoring, stop rules, escalation pathways, physiological monitoring	Safety, fidelity, feasibility	Standardized safety governance may improve transferability across sites.
Data capture and documentation gaps	Process: reflecting/evaluating; Inner Setting: information systems	Device logs, EHR fields, audit feedback, decision-support tools	Fidelity, adoption, sustainability	Routine metrics may support audit, learning, and service refinement.
Technical support and maintenance	Intervention Characteristics: complexity; Inner Setting: resources	Vendor/biomedical support, maintenance pathway, troubleshooting escalation	Feasibility, sustainability	Downtime and repair pathways should be measured as implementation outcomes.
Funding, reimbursement, and procurement	Outer Setting: financing; Intervention Characteristics: cost	Budget-impact analysis, service model redesign, utilization monitoring, network planning	Implementation cost, penetration, sustainability	Economic viability depends on utilization, staffing ratios, and reimbursement context.
Regional access and service configuration	Outer Setting: patient needs; Process: planning	Hub-and-spoke models, shared protocols, training coverage, network governance	Penetration, sustainability, equity/access	Networked models may reduce access inequities if governance and workforce capacity are sustained.

Note. The matrix summarizes co-reported determinants, strategies, implementation outcomes and service outcomes and states propositions for prospective evaluation. Safety and equity/access are treated as service outcomes rather than Proctor implementation outcomes.
Abbreviations: CFIR, Consolidated Framework for Implementation Research; ERIC, Expert Recommendations for Implementing Change; Proctor outcomes, implementation outcomes taxonomy comprising acceptability, adoption, appropriateness, feasibility, fidelity, penetration, sustainability, and implementation cost.

Supplementary Table S10. Minimum reporting set for routine-care rehabilitation robotics implementation

Reporting element	What to report	Suggested operational metrics	Transferability question	Reporting value
Staffing and supervision	Staff roles, supervision model, staff-to-patient ratio, use of assistants, and responsibility for monitoring.	Staff minutes/session; staff-to-patient ratio; denominator = delivered sessions; collect during rollout and quarterly.	Can another service reproduce the staffing model?	Separates device effects from service-delivery intensity.
Training and competency	Initial onboarding, manufacturer training, competency checks, refresher sessions, and champion/super-user structure.	Training hours; certified users/eligible staff; competency completion; refresher frequency; reassess after turnover.	Can staff maintain competency after turnover?	Supports sustainment beyond early enthusiasm or vendor support.
Workflow and scheduling	Session structure, setup/donning/doffing, slot allocation, circuit design, throughput, and competing therapy priorities.	Setup and active minutes; sessions/week; slot fill; cancellations; throughput; report per session and monthly.	Does the pathway fit ordinary schedules?	Makes feasibility measurable rather than anecdotal.
Screening and eligibility	Inclusion/exclusion criteria, contraindications, patient fit, cognitive/behavioural requirements, and reasons for non-use.	Screened, eligible and treated counts; exclusion/withdrawal reasons; report reach by age, sex, diagnosis, geography and access needs.	Who can actually access the robotic pathway?	Allows interpretation of reach, appropriateness, and equity.
Safety governance	Monitoring, adverse-event definitions, stop rules, emergency procedures, and escalation pathways.	Adverse events and near misses per 1,000 sessions; stop-rule activations; assessor and escalation pathway; report continuously.	Can safety procedures be replicated safely?	Essential for high-staffing gait/exoskeleton services.
Documentation and data capture	Device logs, EHR fields, session templates, dose metrics, adverse-event logs, and audit trail.	Required fields completed/expected fields; device-log/EHR source; missingness; audit monthly or quarterly.	Can the service audit dose and use over time?	Links adoption, fidelity, penetration, and sustainment.
Technical support and maintenance	Troubleshooting pathway, vendor/biomedical support, maintenance schedule, software updates, and downtime handling.	Downtime hours/device-month; response and repair time; planned maintenance completion; responsible service/vendor.	Can the device remain usable after implementation?	Addresses a frequent sustainment blind spot.
Infrastructure and space	Location, storage, floor space, transport, accessibility, and co-location with therapy areas.	Area, storage, accessibility, network and power requirements; energy use/device-hour where feasible; reassess after scale-up.	Can local infrastructure support routine use?	Clarifies why models transfer differently across settings.
Costs and resource assumptions	Capital cost, depreciation, maintenance, training, staff time, reimbursement, opportunity cost, and utilization assumptions.	Capital, depreciation, maintenance, training, staff time, reimbursement, opportunity cost, utilization and sensitivity analysis.	Is the pathway financially viable locally?	Prevents adoption based only on perceived usefulness.
Sustainment and audit	Long-term ownership, competency retention, protocol updates, utilization review, feedback loops, and planned continuation.	Acceptability, adoption, appropriateness, feasibility, fidelity, penetration, sustainability and implementation cost at prespecified time points, including 6–12 months.	Will the pathway persist after pilot funding?	Distinguishes early adoption from durable implementation.
Measurement specification	For every implementation metric, define unit, denominator, data source, responsible assessor, collection time point and follow-up period.	Dictionary completeness; prespecified source and owner; collection schedule; missing-data rule.	Can another service calculate the metric in the same way?	Prevents nominally similar outcomes from using incomparable definitions.

Note. This minimum reporting set is an evidence-informed proposal derived from the included literature. Prospective validation and stakeholder consensus are required before use as a formal standard.
Abbreviations: EHR, electronic health record; RAGT, robot-assisted gait training; RR, rehabilitation robotics; t-RAGT, treadmill-based robot-assisted gait training.
